# Supplementary material for: The Role of Neutral Sphingomyelinase-2 (NSM2) in the Control of Neutral Lipid Storage in T Cells
Source: Int J Mol Sci. 2024 Mar 13;25(6):3247. doi: 10.3390/ijms25063247 (PMC10970209; doi:10.3390/ijms25063247)
Supplement: Supplementary file 1 [file ijms-25-03247-s001.zip › MDPI Schempp et al Supplementary Tables.pdf]

Supplementary Tables

The role of Neutral sphingomyelinase-2 (NSM2) in the control of neutral lipid storage in T cells

Rebekka Schempp [1], Janna Eilts [2], Marie Schöl [1], Maria Fernanda Grijalva Yépez [1], Agnes Fekete [3], Dominik Wigger [4], Fabian Schumacher [4], Burkhard Kleuser [4], Marco van Ham [5], Lothar Jänsch [5], Markus Sauer [2], Elita Avota [1]*

[1] Institute for Virology and Immunobiology, University of Würzburg, 97078 Würzburg, Germany
[2] Department of Biotechnology and Biophysics, Biocenter, University of Würzburg, 97074 Würzburg, Germany
[3] Pharmaceutical Biology, Julius-von-Sachs-Institute, Biocenter, University of Würzburg, 97082 Würzburg, Germany
[4] Institute of Pharmacy, Department of Pharmacology and Toxicology, Freie Universität Berlin, 14195 Berlin, Germany
[5] Cellular Proteome Research Group, Helmholtz Centre for Infection Research, 38124 Braunschweig, Germany
* Correspondence: elita.avota@uni-wuerzburg.de

Table S1. 1314 significantly enriched proteins detected in nano-environment of NSM2 and identified by protein mass spectrometry. 34 significantly and highly enriched proteins are marked in bold.

Gene Symbol	Protein Accession ID	Description	log2 (+H2O2/-H2O2)	-log10 (p value)	Cut Off
**HGS**	O14964	hepatocyte growth factor-regulated tyrosine kinase substrate	5,71	3,87	PASS
**CARS1**	B4DKY1	cysteinyl-tRNA synthetase 1	5,16	4,37	PASS
**SUZ12**	Q15022	SUZ12 polycomb repressive complex 2 subunit	4,78	4,19	PASS
**AMPD2**	Q01433	adenosine monophosphate deaminase 2	4,60	5,45	PASS
**JPT2**	H3BMV3	Jupiter microtubule associated homolog 2	4,51	4,36	PASS
**TFG**	Q92734	trafficking from ER to golgi regulator	4,32	6,11	PASS
**BOLA1**	Q9Y3E2	bolA family member 1	4,29	4,07	PASS
**LCP1**	P13796	lymphocyte cytosolic protein 1	4,28	6,76	PASS
**ANXA6**	P08133	annexin A6	4,19	3,79	PASS
**CAST**	A0A0C4DGB5	calpastatin	4,17	4,79	PASS
**PLIN3**	O60664	perilipin 3	4,17	4,02	PASS
**SMN1**	Q16637	survival of motor neuron 1, telomeric	4,12	5,46	PASS
**MOCS2**	O96007	molybdenum cofactor synthesis 2	4,09	4,52	PASS
**CARHSP1**	Q9Y2V2	calcium regulated heat stable protein 1	4,04	5,17	PASS
**APRT**	P07741	adenine phosphoribosyltransferase	4,04	4,78	PASS
**SNX9**	Q9Y5X1	sorting nexin 9	3,99	4,51	PASS
**CNN2**	B4DDF4	calponin 2	3,99	4,05	PASS
**APIP**	Q96GX9	APAF1 interacting protein	3,96	4,43	PASS
**TAGLN2**	P37802	transgelin 2	3,89	4,71	PASS
**FYB1**	O15117	FYN binding protein 1	3,85	4,59	PASS
**IST1**	H3BMU1	IST1 factor associated with ESCRT-III	3,80	5,55	PASS
**HAUS5**	O94927	HAUS augmin like complex subunit 5	3,74	4,72	PASS
**CHAMP1**	Q96JM3	chromosome alignment maintaining phosphoprotein 1	3,73	4,38	PASS
**GBP1**	P32455	guanylate binding protein 1	3,73	4,27	PASS
**PGP**	A6NDG6	phosphoglycolate phosphatase	3,73	4,74	PASS
**CCDC88C**	Q9P219	coiled-coil domain containing 88C	3,71	4,35	PASS
**TBC1D15**	Q8TC07	TBC1 domain family member 15	3,69	3,71	PASS

**STAM**	Q92783	signal transducing adaptor molecule	3,65	5,38	PASS
**NEDD1**	Q8NHV4	NEDD1 gamma-tubulin ring complex targeting factor	3,58	4,03	PASS
**IMPDH1**	A0A3B3IRL5	inosine monophosphate dehydrogenase 1	3,55	4,31	PASS
**INPP4A**	Q96PE3	inositol polyphosphate-4-phosphatase type I A	3,51	4,71	PASS
**ERAP1**	Q9NZ08	endoplasmic reticulum aminopeptidase 1	3,51	4,16	PASS
**RRAS2**	P62070	RAS related 2	3,68	4,16	PASS
**SESN2**	P58004	sestrin 2	3,75	4,02	PASS
SLC39A14	Q15043	solute carrier family 39 member 14	7,17	1,95	FAIL
MTR	Q99707	5-methyltetrahydrofolate-homocysteine methyltransfer-ase	6,36	2,06	FAIL
PFDN4	E9PQY2	prefoldin subunit 4	6,08	1,76	FAIL
HCLS1	P14317	hematopoietic cell-specific Lyn substrate 1	6,05	2,57	FAIL
ADSS2	P30520	adenylosuccinate synthase 2	5,99	2,40	FAIL
TMOD3	Q9NYL9	tropomodulin 3	5,74	1,85	FAIL
VPS37B	Q9H9H4	VPS37B subunit of ESCRT-I	5,70	2,55	FAIL
ACTN4	O43707	actinin alpha 4	5,67	2,13	FAIL
AARSD1	Q9BTE6	alanyl-tRNA synthetase domain containing 1	5,66	1,81	FAIL
SASH3	O75995	SAM and SH3 domain containing 3	5,60	2,06	FAIL
LUZP1	Q86V48	leucine zipper protein 1	5,54	2,39	FAIL
IQSEC1	A0A3B3IRZ4	IQ motif and Sec7 domain ArfGEF 1	5,47	2,26	FAIL
FRMD4A	Q9P2Q2	FERM domain containing 4A	5,46	1,63	FAIL
CCDC6	Q16204	coiled-coil domain containing 6	5,45	2,26	FAIL
STXBP2	Q15833	syntaxin binding protein 2	5,42	3,50	FAIL
SRGAP2	O75044	SLIT-ROBO Rho GTPase activating protein 2	5,36	2,41	FAIL
LCP2	Q13094	lymphocyte cytosolic protein 2	5,35	1,40	FAIL
NSFL1C	Q9UNZ2	NSFL1 cofactor	5,34	2,43	FAIL
GPHN	Q9NQX3	gephyrin	5,33	2,84	FAIL
APBB1IP	Q7Z5R6	amyloid beta precursor protein binding family B mem-ber 1 interacting protein	5,30	2,80	FAIL
LASP1	Q14847	LIM and SH3 protein 1	5,28	1,99	FAIL
PLPBP	O94903	pyridoxal phosphate binding protein	5,22	1,81	FAIL
NCOR1	O75376	nuclear receptor corepressor 1	5,20	2,34	FAIL
TRPV2	Q9Y5S1	transient receptor potential cation channel subfamily V member 2	5,19	1,47	FAIL
ADD1	E7ENY0	adducin 1	5,17	2,80	FAIL
SBF1	O95248	SET binding factor 1	5,16	1,46	FAIL
PFDN6	O15212	prefoldin subunit 6	5,15	2,09	FAIL
ARFGAP3	Q9NP61	ADP ribosylation factor GTPase activating protein 3	5,14	1,95	FAIL
PTPN11	Q06124	protein tyrosine phosphatase non-receptor type 11	5,12	2,77	FAIL
UBE2R2	Q712K3	ubiquitin conjugating enzyme E2 R2	5,09	2,67	FAIL
EFHD1	Q9BUP0	EF-hand domain family member D1	5,08	2,62	FAIL
DCTN2	Q13561	dynactin subunit 2	5,06	2,59	FAIL
MAPRE1	Q15691	microtubule associated protein RP/EB family member 1	5,04	3,52	FAIL
CEP55	Q53EZ4	centrosomal protein 55	5,03	2,17	FAIL
RIPK1	Q13546	receptor interacting serine/threonine kinase 1	5,03	2,72	FAIL
TPT1	Q5W0H4	tumor protein, translationally-controlled 1	5,00	2,51	FAIL
CACYBP	Q9HB71	calcyclin binding protein	4,94	2,94	FAIL

SRI	P30626	sorcin	4,93	2,80	FAIL
TPM3	A0A087WWU8	tropomyosin 3	4,93	1,87	FAIL
PAWR	Q96IZ0	pro-apoptotic WT1 regulator	4,91	2,10	FAIL
PRPF8	Q6P2Q9	pre-mRNA processing factor 8	4,90	1,41	FAIL
PSMD9	F5H5V4	proteasome 26S subunit, non-ATPase 9	4,89	1,52	FAIL
STAT5B	P51692	signal transducer and activator of transcription 5B	4,87	2,87	FAIL
PSMG1	O95456	proteasome assembly chaperone 1	4,85	2,61	FAIL
RANBP9	Q96S59	RAN binding protein 9	4,85	2,55	FAIL
GOPC	Q9HD26	golgi associated PDZ and coiled-coil motif containing	4,84	2,15	FAIL
MED24	F5H5K2	mediator complex subunit 24	4,83	2,85	FAIL
DPYSL2	A0A1C7CYX9	dihydropyrimidinase like 2	4,80	2,12	FAIL
TNKS1BP1	Q9C0C2	tankyrase 1 binding protein 1	4,79	2,21	FAIL
	F6TLX2		4,78	2,45	FAIL
ATP7A	Q04656	ATPase copper transporting alpha	4,78	3,31	FAIL
NFATC3	H3BRS1	nuclear factor of activated T cells 3	4,76	2,60	FAIL
WBP2	Q969T9	WW domain binding protein 2	4,76	1,89	FAIL
SMYD5	Q6GMV2	SMYD family member 5	4,76	2,32	FAIL
DSTN	F6RFD5	destrin, actin depolymerizing factor	4,75	2,94	FAIL
UXT	Q9UBK9	ubiquitously expressed prefoldin like chaperone	4,74	1,35	FAIL
PBK	Q96KB5	PDZ binding kinase	4,72	2,49	FAIL
PLCB3	Q01970	phospholipase C beta 3	4,70	1,50	FAIL
EIF4B	E7EX17	eukaryotic translation initiation factor 4B	4,69	2,10	FAIL
CASP2	P42575	caspase 2	4,66	1,65	FAIL
KCNAB2	A0A5F9UN28	potassium voltage-gated channel subfamily A regulatory beta subunit 2	4,64	1,87	FAIL
DNMBP	Q6XZF7	dynamin binding protein	4,64	2,34	FAIL
PJA2	O43164	praja ring finger ubiquitin ligase 2	4,62	1,99	FAIL
CIAPIN1	Q6FI81	cytokine induced apoptosis inhibitor 1	4,61	2,66	FAIL
RABGAP1L	Q5R372	RAB GTPase activating protein 1 like	4,60	2,35	FAIL
PNPO	J3QQZ9	pyridoxamine 5'-phosphate oxidase	4,60	2,04	FAIL
NSMCE1	Q8WV22	NSE1 homolog, SMC5-SMC6 complex component	4,59	2,79	FAIL
TPM3	J3KN67	tropomyosin 3	4,59	2,03	FAIL
TNIP1	Q15025	TNFAIP3 interacting protein 1	4,58	3,01	FAIL
PMF1-BGLAP	A0A087WT04	PMF1-BGLAP readthrough	4,57	1,87	FAIL
MVB12A	Q96EY5	multivesicular body subunit 12A	4,57	2,84	FAIL
CLSPN	Q9HAW4	claspin	4,54	1,80	FAIL
NT5DC1	Q5TFE4	5'-nucleotidase domain containing 1	4,52	1,94	FAIL
WWP2	O00308	WW domain containing E3 ubiquitin protein ligase 2	4,51	1,97	FAIL
	E9PL57		4,51	1,47	FAIL
PDLIM2	Q96JY6	PDZ and LIM domain 2	4,47	2,57	FAIL
MITD1	H7C3Q6	microtubule interacting and trafficking domain containing 1	4,47	2,64	FAIL
TBCB	A0A494C0X0	tubulin folding cofactor B	4,46	2,14	FAIL
ALDOC	P09972	aldolase, fructose-bisphosphate C	4,45	2,23	FAIL
NDUFS1	P28331	NADH:ubiquinone oxidoreductase core subunit S1	4,44	1,78	FAIL
TANGO6	Q9C0B7	transport and golgi organization 6 homolog	4,44	1,96	FAIL
GIMAP7	Q8NHV1	GTPase, IMAP family member 7	4,43	2,23	FAIL

NSUN6	Q8TEA1	NOP2/Sun RNA methyltransferase 6	4,43	1,43	FAIL
FERMT2	H0YJ34	FERM domain containing kindlin 2	4,43	2,46	FAIL
DHX16	O60231	DEAH-box helicase 16	4,43	2,95	FAIL
ACTN1	P12814	actinin alpha 1	4,42	2,54	FAIL
ADD3	Q9UEY8	adducin 3	4,42	2,45	FAIL
CORO1C	Q9ULV4	coronin 1C	4,40	1,97	FAIL
FTSJ3	Q8IY81	FtsJ RNA 2'-O-methyltransferase 3	4,40	2,51	FAIL
MACO1	Q8N5G2	macoilin 1	4,39	1,80	FAIL
PDCD5	O14737	programmed cell death 5	4,39	1,94	FAIL
TBCE	Q15813	tubulin folding cofactor E	4,38	2,90	FAIL
PLCG1	P19174	phospholipase C gamma 1	4,37	2,20	FAIL
FKBP15	Q5T1M5	FKBP prolyl isomerase family member 15	4,37	2,01	FAIL
EIF4A2	Q14240	eukaryotic translation initiation factor 4A2	4,37	1,73	FAIL
ANKRD52	Q8NB46	ankyrin repeat domain 52	4,36	2,27	FAIL
	L0R819		4,36	3,01	FAIL
ASMTL	O95671	acetylserotonin O-methyltransferase like	4,36	1,51	FAIL
TBC1D17	Q9HA65	TBC1 domain family member 17	4,35	3,09	FAIL
TSN	Q15631	translin	4,34	2,01	FAIL
CTU2	H3BSW6	cytosolic thiouridylase subunit 2	4,31	1,39	FAIL
FHIP2A	Q5W0V3	FHF complex subunit HOOK interacting protein 2A	4,30	2,56	FAIL
DBNL	Q9UJU6	drebrin like	4,29	2,60	FAIL
FAT1	Q14517	FAT atypical cadherin 1	4,28	2,46	FAIL
FAF1	Q9UNN5	Fas associated factor 1	4,27	2,41	FAIL
GYPC	P04921	glycophorin C (Gerbich blood group)	4,26	1,55	FAIL
DUT	P33316	deoxyuridine triphosphatase	4,25	2,34	FAIL
PM20D2	Q8IYS1	peptidase M20 domain containing 2	4,23	2,28	FAIL
SLC4A7	H7C3C4	solute carrier family 4 member 7	4,20	3,52	FAIL
UCHL5	Q5LJA5	ubiquitin C-terminal hydrolase L5	4,20	3,25	FAIL
VASP	P50552	vasodilator stimulated phosphoprotein	4,20	2,72	FAIL
PPP4R3A	Q6IN85	protein phosphatase 4 regulatory subunit 3A	4,20	1,57	FAIL
ATG4B	Q9Y4P1	autophagy related 4B cysteine peptidase	4,18	1,42	FAIL
	A0A087WZK9		4,18	2,55	FAIL
TTC1	Q99614	tetratricopeptide repeat domain 1	4,18	2,04	FAIL
SPC25	Q9HBM1	SPC25 component of NDC80 kinetochore complex	4,18	3,52	FAIL
ADO	Q96SZ5	2-aminoethanethiol dioxygenase	4,17	2,28	FAIL
CBX5	P45973	chromobox 5	4,16	2,27	FAIL
SASS6	Q6UVJ0	SAS-6 centriolar assembly protein	4,16	1,92	FAIL
PPP1R9B	Q96SB3	protein phosphatase 1 regulatory subunit 9B	4,16	1,92	FAIL
HDGF	P51858	heparin binding growth factor	4,16	1,44	FAIL
DCP1B	Q8IZD4	decapping mRNA 1B	4,15	2,78	FAIL
IKBKB	O14920	inhibitor of nuclear factor kappa B kinase subunit beta	4,15	2,04	FAIL
CASKIN2	Q8WXE0	CASK interacting protein 2	4,15	2,78	FAIL
TSG101	F5H442	tumor susceptibility 101	4,14	3,03	FAIL
DLG1	Q12959	discs large MAGUK scaffold protein 1	4,14	2,03	FAIL
RAPGEF1	Q13905	Rap guanine nucleotide exchange factor 1	4,13	3,20	FAIL
VBP1	P61758	VHL binding protein 1	4,13	2,37	FAIL
SPTAN1	Q13813	spectrin alpha, non-erythrocytic 1	4,12	2,90	FAIL
LRSAM1	A0A6Q8PFL5	leucine rich repeat and sterile alpha motif containing 1	4,12	1,82	FAIL

MSL1	Q68DK7	MSL complex subunit 1	4,11	2,37	FAIL
SULF2	Q8IWU5	sulfatase 2	4,11	2,12	FAIL
CDC73	Q6P1J9	cell division cycle 73	4,11	1,56	FAIL
SPART	Q8N0X7	spartin	4,11	2,70	FAIL
PTPN23	Q9H3S7	protein tyrosine phosphatase non-receptor type 23	4,08	3,40	FAIL
MTPN	P58546	myotrophin	4,08	2,24	FAIL
HECTD3	Q5T447	HECT domain E3 ubiquitin protein ligase 3	4,06	2,07	FAIL
TSPYL5	Q86VY4	TSPY like 5	4,05	2,07	FAIL
STIM2	Q9P246	stromal interaction molecule 2	4,04	1,43	FAIL
SLC39A10	Q9ULF5	solute carrier family 39 member 10	4,04	2,12	FAIL
CAP1	Q01518	cyclase associated actin cytoskeleton regulatory protein 1	4,04	2,15	FAIL
ZC3HC1	Q86WB0	zinc finger C3HC-type containing 1	4,03	3,46	FAIL
GAPDH	P04406	glyceraldehyde-3-phosphate dehydrogenase	4,02	2,31	FAIL
PRAG1	Q86YV5	PEAK1 related, kinase-activating pseudokinase 1	4,02	2,65	FAIL
MTMR12	Q9C0I1	myotubularin related protein 12	4,02	2,99	FAIL
DNAJC2	Q99543	DnaJ heat shock protein family (Hsp40) member C2	4,01	2,39	FAIL
CSTB	P04080	cystatin B	4,01	1,32	FAIL
LARP7	Q4G0J3	La ribonucleoprotein 7, transcriptional regulator	4,01	3,11	FAIL
RNF31	Q96EP0	ring finger protein 31	4,00	2,24	FAIL
VAMP5	O95183	vesicle associated membrane protein 5	3,99	2,24	FAIL
OBI1	Q5W0B1	ORC ubiquitin ligase 1	3,99	2,15	FAIL
NISCH	Q9Y2I1	nischarin	3,99	3,29	FAIL
JMJD6	B2WTI3	jumonji domain containing 6, arginine demethylase and lysine hydroxylase	3,99	1,37	FAIL
PPA1	Q15181	inorganic pyrophosphatase 1	3,98	1,99	FAIL
NAA30	Q147X3	N-alpha-acetyltransferase 30, NatC catalytic subunit	3,97	1,86	FAIL
SMPD3	Q9NY59	sphingomyelin phosphodiesterase 3	3,97	2,08	FAIL
SLC25A40	Q8TBP6	solute carrier family 25 member 40	3,96	2,09	FAIL
FASTKD5	Q7L8L6	FAST kinase domains 5	3,96	1,45	FAIL
FNBP1	B7ZL14	formin binding protein 1	3,96	2,85	FAIL
CSRP1	E9PP21	cysteine and glycine rich protein 1	3,95	2,48	FAIL
ECSIT	Q9BQ95	ECSIT signaling integrator	3,94	1,56	FAIL
SNAP23	O00161	synaptosome associated protein 23	3,94	2,60	FAIL
UBR7	Q8N806	ubiquitin protein ligase E3 component n-recognin 7	3,93	1,94	FAIL
AFG2B	Q9BVQ7	AFG2 AAA ATPase homolog B	3,93	2,17	FAIL
GLE1	Q53GS7	GLE1 RNA export mediator	3,92	2,82	FAIL
PCYT2	Q99447	phosphate cytidylyltransferase 2, ethanolamine	3,92	2,81	FAIL
PPIP5K2	O43314	diphosphoinositol pentakisphosphate kinase 2	3,92	2,43	FAIL
RNPEP	A6NKB8	arginyl aminopeptidase	3,92	1,88	FAIL
ERBIN	Q96RT1	erbb2 interacting protein	3,92	2,66	FAIL
EIF2B2	P49770	eukaryotic translation initiation factor 2B subunit beta	3,92	1,91	FAIL
EPHB2	B1AKC9	EPH receptor B2	3,91	2,04	FAIL
RPAP3	Q9H6T3	RNA polymerase II associated protein 3	3,90	2,10	FAIL
ARHGAP25	P42331	Rho GTPase activating protein 25	3,89	1,49	FAIL
DOK2	O60496	docking protein 2	3,89	2,83	FAIL
GEMIN8	Q9NWZ8	gem nuclear organelle associated protein 8	3,88	2,50	FAIL

STK10	O94804	serine/threonine kinase 10	3,87	2,06	FAIL
PRMT1	E9PKG1	protein arginine methyltransferase 1	3,87	2,93	FAIL
SH3GL1	Q99961	SH3 domain containing GRB2 like 1, endophilin A2	3,86	3,48	FAIL
BIRC6	Q9NR09	baculoviral IAP repeat containing 6	3,86	2,65	FAIL
EPB41L2	H0Y5B0	erythrocyte membrane protein band 4.1 like 2	3,86	1,96	FAIL
SPTBN2	O15020	spectrin beta, non-erythrocytic 2	3,85	2,09	FAIL
WDR26	A0A499FIZ0	WD repeat domain 26	3,84	2,36	FAIL
HIRA	P54198	histone cell cycle regulator	3,84	2,05	FAIL
MON2	A0A286YFF8	MON2 homolog, regulator of endosome-to-Golgi trafficking	3,83	2,86	FAIL
PAICS	P22234	phosphoribosylaminoimidazole carboxylase and phosphoribosylaminoimidazolesuccinocarboxamide synthase	3,82	3,23	FAIL
BRK1	Q8WUW1	BRICK1 subunit of SCAR/WAVE actin nucleating complex	3,81	2,80	FAIL
PFN1	P07737	profilin 1	3,81	2,34	FAIL
PACSIN2	Q9UNF0	protein kinase C and casein kinase substrate in neurons 2	3,81	2,72	FAIL
UFC1	Q9Y3C8	ubiquitin-fold modifier conjugating enzyme 1	3,80	2,82	FAIL
RTF2	A0A0A0MQR2	replication termination factor 2	3,80	1,70	FAIL
COG4	J3KNI1	component of oligomeric golgi complex 4	3,80	2,07	FAIL
HSD17B7	P56937	hydroxysteroid 17-beta dehydrogenase 7	3,80	2,41	FAIL
PPP5C	H0YDU8	protein phosphatase 5 catalytic subunit	3,79	2,43	FAIL
HPF1	Q9NWY4	histone PARylation factor 1	3,79	1,61	FAIL
PFDN2	Q9UHV9	prefoldin subunit 2	3,78	2,39	FAIL
FCHO1	O14526	FCH and mu domain containing endocytic adaptor 1	3,78	1,70	FAIL
EPB41	P11171	erythrocyte membrane protein band 4.1	3,78	2,84	FAIL
CASP8	Q14790	caspase 8	3,78	2,30	FAIL
CBLB	Q13191	Cbl proto-oncogene B	3,77	1,31	FAIL
ABHD12	Q8N2K0	abhydrolase domain containing 12, lysophospholipase	3,77	1,88	FAIL
REL	Q04864	REL proto-oncogene, NF-kB subunit	3,76	3,32	FAIL
C5	P01031	complement C5	3,76	1,41	FAIL
LPXN	O60711	leupaxin	3,76	2,02	FAIL
MAT2A	P31153	methionine adenosyltransferase 2A	3,76	1,94	FAIL
DYNC1I2	Q13409	dynein cytoplasmic 1 intermediate chain 2	3,75	2,75	FAIL
RRM1	P23921	ribonucleotide reductase catalytic subunit M1	3,74	1,90	FAIL
EEF1B2	P24534	eukaryotic translation elongation factor 1 beta 2	3,74	2,48	FAIL
WASF1	Q92558	WASP family member 1	3,74	1,90	FAIL
RPP38	P78345	ribonuclease P/MRP subunit p38	3,73	1,84	FAIL
TTI2	E5RIH5	TELO2 interacting protein 2	3,73	2,29	FAIL
CNNM4	Q6P4Q7	cyclin and CBS domain divalent metal cation transport mediator 4	3,73	3,59	FAIL
UACA	H0YNH8	uveal autoantigen with coiled-coil domains and ankyrin repeats	3,72	1,76	FAIL
PPFIA1	Q13136	PTPRF interacting protein alpha 1	3,72	1,82	FAIL
HNRNPDL	O14979	heterogeneous nuclear ribonucleoprotein D like	3,72	2,93	FAIL
MORF4L2	Q5JXX2	mortality factor 4 like 2	3,72	1,51	FAIL

XPNPEP1	Q9NQW7	X-prolyl aminopeptidase 1	3,71	2,31	FAIL
CHORDC1	Q9UHD1	cysteine and histidine rich domain containing 1	3,70	2,29	FAIL
STAU1	O95793	staufen double-stranded RNA binding protein 1	3,70	3,50	FAIL
KNL1	Q8NG31	kinetochore scaffold 1	3,69	1,75	FAIL
ARHGEF18	A0A087WZG4	Rho/Rac guanine nucleotide exchange factor 18	3,69	2,39	FAIL
EMSY	Q7Z589	EMSY transcriptional repressor, BRCA2 interacting	3,68	2,02	FAIL
EZR	P15311	ezrin	3,68	2,57	FAIL
ATP6V0D1	F5GYQ1	ATPase H+ transporting V0 subunit d1	3,68	3,53	FAIL
MAP3K7	O43318	mitogen-activated protein kinase kinase kinase 7	3,68	1,75	FAIL
CHURC1-FNTB	P49356	CHURC1-FNTB readthrough	3,67	3,36	FAIL
CCP110	O43303	centriolar coiled-coil protein 110	3,67	2,38	FAIL
ARHGDIA	P52565	Rho GDP dissociation inhibitor alpha	3,67	1,49	FAIL
ECHDC1	Q9NTX5	ethylmalonyl-CoA decarboxylase 1	3,66	2,32	FAIL
RANBP3	Q9H6Z4	RAN binding protein 3	3,65	2,16	FAIL
SGPP1	Q9BX95	sphingosine-1-phosphate phosphatase 1	3,64	1,56	FAIL
PRUNE1	Q86TP1	prune exopolyphosphatase 1	3,63	1,37	FAIL
NDUFB8	O95169	NADH:ubiquinone oxidoreductase subunit B8	3,63	2,19	FAIL
USP9X	Q93008	ubiquitin specific peptidase 9 X-linked	3,62	2,08	FAIL
TMF1	P82094	TATA element modulatory factor 1	3,62	1,69	FAIL
DRG2	P55039	developmentally regulated GTP binding protein 2	3,61	2,08	FAIL
TLN1	Q9Y490	talin 1	3,60	2,42	FAIL
GOLGA3	Q08378	golgin A3	3,60	2,09	FAIL
KLC2	Q9H0B6	kinesin light chain 2	3,60	2,35	FAIL
DCK	P27707	deoxycytidine kinase	3,60	1,61	FAIL
FAM162A	F8W7Q4	family with sequence similarity 162 member A	3,60	1,85	FAIL
NFX1	Q12986	nuclear transcription factor, X-box binding 1	3,59	2,28	FAIL
ADI1	Q9BV57	acireductone dioxygenase 1	3,59	1,69	FAIL
RNGTT	O60942	RNA guanylyltransferase and 5'-phosphatase	3,58	1,58	FAIL
CASP3	C9JXR7	caspase 3	3,58	2,69	FAIL
OIP5	O43482	Opa interacting protein 5	3,58	3,06	FAIL
YWHAB	P31946	tyrosine 3-monooxygenase/tryptophan 5-monooxygen-ase activation protein beta	3,58	2,03	FAIL
RABGGTB	Q5T4U8	Rab geranylgeranyltransferase subunit beta	3,57	2,15	FAIL
NF1	P21359	neurofibromin 1	3,57	1,62	FAIL
AGFG1	P52594	ArfGAP with FG repeats 1	3,57	2,13	FAIL
TAPBP	A0A0G2JH37	TAP binding protein	3,56	1,53	FAIL
STK3	Q13188	serine/threonine kinase 3	3,56	2,81	FAIL
EIF4G3	A0A0U1RQK7	eukaryotic translation initiation factor 4 gamma 3	3,56	1,92	FAIL
TRIP12	A0A6Q8PHK0	thyroid hormone receptor interactor 12	3,55	2,56	FAIL
SKA3	Q8IX90	spindle and kinetochore associated complex subunit 3	3,54	2,37	FAIL
NFKB1	P19838	nuclear factor kappa B subunit 1	3,54	3,11	FAIL
BID	P55957	BH3 interacting domain death agonist	3,54	2,01	FAIL
ANKS1A	A0A3B3ISP1	ankyrin repeat and sterile alpha motif domain contain-ing 1A	3,54	1,78	FAIL
PPAT	Q06203	phosphoribosyl pyrophosphate amidotransferase	3,54	1,68	FAIL
CNOT10	Q9H9A5	CCR4-NOT transcription complex subunit 10	3,54	2,08	FAIL

RRAGA	Q7L523	Ras related GTP binding A	3,53	2,00	FAIL
EVL	Q9UI08	Enah/Vasp-like	3,53	2,39	FAIL
MED27	A0A494C0P0	mediator complex subunit 27	3,53	1,83	FAIL
INTS8	Q75QN2	integrator complex subunit 8	3,53	1,41	FAIL
EFHD2	Q96C19	EF-hand domain family member D2	3,53	3,06	FAIL
GRAMD4	Q6IC98	GRAM domain containing 4	3,53	1,75	FAIL
CEP43	O95684	centrosomal protein 43	3,53	2,10	FAIL
IRF2BPL	Q9H1B7	interferon regulatory factor 2 binding protein like	3,52	3,31	FAIL
EPB41L2	O43491	erythrocyte membrane protein band 4.1 like 2	3,52	2,54	FAIL
RNASEH2C	E9PN81	ribonuclease H2 subunit C	3,52	3,07	FAIL
AIP	O00170	aryl hydrocarbon receptor interacting protein	3,52	2,84	FAIL
CALCOCO1	Q9P1Z2	calcium binding and coiled-coil domain 1	3,51	1,87	FAIL
CRACR2A	Q9BSW2	calcium release activated channel regulator 2A	3,51	2,28	FAIL
UBE2Q1	Q7Z7E8	ubiquitin conjugating enzyme E2 Q1	3,51	1,57	FAIL
ZBED4	O75132	zinc finger BED-type containing 4	3,51	2,87	FAIL
COPS6	E7EM64	COP9 signalosome subunit 6	3,51	1,91	FAIL
ANXA11	P50995	annexin A11	3,51	2,53	FAIL
GTF3C6	Q969F1	general transcription factor IIIC subunit 6	3,50	1,36	FAIL
GAB2	Q9UQC2	GRB2 associated binding protein 2	3,50	2,98	FAIL
PFKFB2	O60825	6-phosphofructo-2-kinase/fructose-2,6-biphosphatase 2	3,50	1,78	FAIL
DGKQ	P52824	diacylglycerol kinase theta	3,50	2,50	FAIL
CDK13	A0A2R8Y7W5	cyclin dependent kinase 13	3,49	2,14	FAIL
ANXA7	P20073	annexin A7	3,49	5,37	FAIL
HSPH1	Q92598	heat shock protein family H (Hsp110) member 1	3,49	2,07	FAIL
RFX7	Q2KHR2	regulatory factor X7	3,49	2,85	FAIL
LRBA	A0A494C1L5	LPS responsive beige-like anchor protein	3,49	2,07	FAIL
TACC3	Q9Y6A5	transforming acidic coiled-coil containing protein 3	3,49	3,28	FAIL
PALM2AKAP2	Q9Y2D5	PALM2 and AKAP2 fusion	3,48	5,64	FAIL
YWHAE	P62258	tyrosine 3-monooxygenase/tryptophan 5-monooxygenase activation protein epsilon	3,48	5,19	FAIL
ARFGAP1	Q8N6T3	ADP ribosylation factor GTPase activating protein 1	3,48	3,04	FAIL
PTDSS1	P48651	phosphatidylserine synthase 1	3,48	1,69	FAIL
PEX19	Q5QNY5	peroxisomal biogenesis factor 19	3,47	2,08	FAIL
NUP43	Q8NFH3	nucleoporin 43	3,47	2,03	FAIL
ARHGEF6	Q15052	Rac/Cdc42 guanine nucleotide exchange factor 6	3,47	2,68	FAIL
ARID5B	Q14865	AT-rich interaction domain 5B	3,46	1,76	FAIL
MTSS2	Q765P7	MTSS I-BAR domain containing 2	3,46	2,84	FAIL
ARID4B	Q4LE39	AT-rich interaction domain 4B	3,46	4,05	FAIL
GGNBP2	Q9H3C7	gametogenetin binding protein 2	3,45	1,78	FAIL
	A0A087WUC6		3,45	3,22	FAIL
SSBP3	Q9BWW4	single stranded DNA binding protein 3	3,45	2,53	FAIL
MFN1	Q8IWA4	mitofusin 1	3,45	1,42	FAIL
PRPSAP1	Q14558	phosphoribosyl pyrophosphate synthetase associated protein 1	3,44	1,80	FAIL
PARN	A0A494C0W0	poly(A)-specific ribonuclease	3,44	2,66	FAIL
KIF14	Q15058	kinesin family member 14	3,44	1,59	FAIL

PIP4K2B	P78356	phosphatidylinositol-5-phosphate 4-kinase type 2 beta	3,44	2,46	FAIL
PSME3IP1	H3BTP8	proteasome activator subunit 3 interacting protein 1	3,43	1,84	FAIL
ATG3	Q9NT62	autophagy related 3	3,43	2,53	FAIL
CD5	P06127	CD5 molecule	3,43	4,87	FAIL
CYTH4	Q9UIA0	cytohesin 4	3,43	2,22	FAIL
UBA5	Q9GZZ9	ubiquitin like modifier activating enzyme 5	3,43	2,66	FAIL
BAP1	F8W6N3	BRCA1 associated protein 1	3,43	3,01	FAIL
PEF1	Q9UBV8	penta-EF-hand domain containing 1	3,43	2,41	FAIL
MAGED1	Q9Y5V3	MAGE family member D1	3,42	3,50	FAIL
SEPTIN6	Q14141	septin 6	3,42	2,83	FAIL
CEP85	Q6P2H3	centrosomal protein 85	3,42	1,50	FAIL
XRCC4	Q13426	X-ray repair cross complementing 4	3,42	2,10	FAIL
ANKRD44	Q8N8A2	ankyrin repeat domain 44	3,42	1,81	FAIL
DSN1	Q9H410	DSN1 component of MIS12 kinetochore complex	3,42	2,15	FAIL
TFCP2	Q12800	transcription factor CP2	3,41	1,80	FAIL
ITPRID2	E9PHV5	ITPR interacting domain containing 2	3,41	3,43	FAIL
BPNT1	O95861	3'(2'), 5'-bisphosphate nucleotidase 1	3,41	3,64	FAIL
RASSF5	Q8TEK8	Ras association domain family member 5	3,40	3,36	FAIL
UBA6	A0AVT1	ubiquitin like modifier activating enzyme 6	3,40	2,62	FAIL
POP4	O95707	POP4 homolog, ribonuclease P/MRP subunit	3,40	1,50	FAIL
VPS28	Q9UK41	VPS28 subunit of ESCRT-I	3,40	2,27	FAIL
TRAF2	Q12933	TNF receptor associated factor 2	3,40	2,54	FAIL
HAUS7	Q99871	HAUS augmin like complex subunit 7	3,39	2,15	FAIL
USP32	Q8NFA0	ubiquitin specific peptidase 32	3,39	1,95	FAIL
STRAP	Q9Y3F4	serine/threonine kinase receptor associated protein	3,39	2,69	FAIL
ELP6	Q0PNE2	elongator acetyltransferase complex subunit 6	3,39	4,46	FAIL
CMTR1	Q8N1G2	cap methyltransferase 1	3,38	2,18	FAIL
ZNF444	Q8N0Y2	zinc finger protein 444	3,38	2,84	FAIL
ELP5	Q8TE02	elongator acetyltransferase complex subunit 5	3,37	1,98	FAIL
GPN3	Q9UHW5	GPN-loop GTPase 3	3,37	1,33	FAIL
PNMA2	Q9UL42	PNMA family member 2	3,37	1,60	FAIL
C10orf88	Q9H8K7	chromosome 10 open reading frame 88	3,36	2,47	FAIL
CDCA5	B5MBX0	cell division cycle associated 5	3,36	2,03	FAIL
BRD3	Q15059	bromodomain containing 3	3,35	1,65	FAIL
CLUH	O75153	clustered mitochondria homolog	3,34	3,37	FAIL
DOCK7	Q96N67	dedicator of cytokinesis 7	3,34	1,73	FAIL
MED12	Q93074	mediator complex subunit 12	3,34	2,57	FAIL
PKN1	Q16512	protein kinase N1	3,34	2,61	FAIL
ARHGAP30	Q7Z6I6	Rho GTPase activating protein 30	3,34	3,28	FAIL
MKLN1	Q9UL63	muskelin 1	3,34	2,39	FAIL
DNAJC21	Q5F1R6	DnaJ heat shock protein family (Hsp40) member C21	3,34	1,44	FAIL
PRDX5	P30044	peroxiredoxin 5	3,34	1,41	FAIL
RABGEF1	E9PFK9	RAB guanine nucleotide exchange factor 1	3,34	2,40	FAIL
SQSTM1	Q13501	sequestosome 1	3,34	1,94	FAIL
ZPR1	O75312	ZPR1 zinc finger	3,34	2,13	FAIL
OTUD6B	A0A087X0W9	OTU deubiquitinase 6B	3,34	1,85	FAIL
SIT1	Q9Y3P8	signaling threshold regulating transmembrane adaptor 1	3,34	2,81	FAIL

SLC38A2	Q96QD8	solute carrier family 38 member 2	3,34	1,37	FAIL
WDR5	P61964	WD repeat domain 5	3,33	2,33	FAIL
MAP1S	Q66K74	microtubule associated protein 1S	3,33	1,68	FAIL
ELP3	Q9H9T3	elongator acetyltransferase complex subunit 3	3,33	2,76	FAIL
ADSL	A0A1B0GTJ7	adenylosuccinate lyase	3,33	4,53	FAIL
GRAP2	O75791	GRB2 related adaptor protein 2	3,33	2,91	FAIL
RFX1	P22670	regulatory factor X1	3,33	2,32	FAIL
CISD2	Q8N5K1	CDGSH iron sulfur domain 2	3,32	1,48	FAIL
HMGCS1	Q01581	3-hydroxy-3-methylglutaryl-CoA synthase 1	3,32	2,63	FAIL
MCTS1	Q9ULC4	MCTS1 re-initiation and release factor	3,32	1,99	FAIL
PTPRCAP	Q14761	protein tyrosine phosphatase receptor type C associated protein	3,31	1,78	FAIL
ADD2	P35612	adducin 2	3,31	1,35	FAIL
TBK1	A0A494C045	TANK binding kinase 1	3,30	1,95	FAIL
SDE2	Q6IQ49	SDE2 telomere maintenance homolog	3,30	1,67	FAIL
CTDP1	Q9Y5B0	CTD phosphatase subunit 1	3,30	2,34	FAIL
HEATR6	Q6AI08	HEAT repeat containing 6	3,30	1,85	FAIL
EMC1	Q8N766	ER membrane protein complex subunit 1	3,29	1,44	FAIL
ELP4	A0A1W2PNY5	elongator acetyltransferase complex subunit 4	3,29	1,77	FAIL
EXOSC3	Q9NQT5	exosome component 3	3,29	1,81	FAIL
USP16	Q9Y5T5	ubiquitin specific peptidase 16	3,29	1,38	FAIL
COG7	P83436	component of oligomeric golgi complex 7	3,29	2,22	FAIL
PPP1R12A	O14974	protein phosphatase 1 regulatory subunit 12A	3,28	2,80	FAIL
AATF	Q9NY61	apoptosis antagonizing transcription factor	3,28	1,79	FAIL
PDE12	F6T1Q0	phosphodiesterase 12	3,28	2,51	FAIL
EIF3L	Q9Y262	eukaryotic translation initiation factor 3 subunit L	3,28	1,52	FAIL
TARBP2	Q15633	TARBP2 subunit of RISC loading complex	3,28	2,34	FAIL
EIF4E2	O60573	eukaryotic translation initiation factor 4E family member 2	3,28	1,94	FAIL
MCAT	Q8IVS2	malonyl-CoA-acyl carrier protein transacylase	3,28	2,03	FAIL
GOLGA1	Q92805	golgin A1	3,27	1,80	FAIL
FDPS	P14324	farnesyl diphosphate synthase	3,26	3,63	FAIL
GMDS	O60547	GDP-mannose 4,6-dehydratase	3,26	2,82	FAIL
WDR41	Q9HAD4	WD repeat domain 41	3,26	1,64	FAIL
MED15	G3V1P5	mediator complex subunit 15	3,25	2,64	FAIL
PRRC1	Q96M27	proline rich coiled-coil 1	3,25	1,62	FAIL
ALDOA	P04075	aldolase, fructose-bisphosphate A	3,25	4,49	FAIL
AARS1	A0A6Q8PFY2	alanyl-tRNA synthetase 1	3,24	2,39	FAIL
ATRX	P46100	ATRX chromatin remodeler	3,24	2,22	FAIL
FAM114A2	Q9NRY5	family with sequence similarity 114 member A2	3,24	1,85	FAIL
GRK3	P35626	G protein-coupled receptor kinase 3	3,24	1,49	FAIL
CCNYL1	Q8N7R7	cyclin Y like 1	3,23	1,60	FAIL
USP34	Q70CQ2	ubiquitin specific peptidase 34	3,23	2,74	FAIL
HDAC6	Q9UBN7	histone deacetylase 6	3,23	1,49	FAIL
TNFRSF8	P28908	TNF receptor superfamily member 8	3,23	2,76	FAIL
SCRIB	A0A669KB89	scribble planar cell polarity protein	3,20	4,60	FAIL
NCKIPSD	Q9NZQ3	NCK interacting protein with SH3 domain	3,20	2,17	FAIL

METTL17	Q9H7H0	methyltransferase like 17	3,20	2,03	FAIL
C9orf72	A0A2R8Y5K2	C9orf72-SMCR8 complex subunit	3,20	1,94	FAIL
STX5	Q13190	syntaxin 5	3,19	3,07	FAIL
TSNAX	Q99598	translin associated factor X	3,19	2,77	FAIL
UPRT	E9PSD7	uracil phosphoribosyltransferase homolog	3,19	3,97	FAIL
CNNM3	Q8NE01	cyclin and CBS domain divalent metal cation transport mediator 3	3,19	4,80	FAIL
ACP1	P24666	acid phosphatase 1	3,18	2,29	FAIL
KNSTRN	Q9Y448	kinetochore localized astrin (SPAG5) binding protein	3,18	2,20	FAIL
SMARCC2	Q8TAQ2	SWI/SNF related, matrix associated, actin dependent regulator of chromatin subfamily c member 2	3,18	1,70	FAIL
NUP54	Q7Z3B4	nucleoporin 54	3,18	2,15	FAIL
BOLA2	Q9H3K6	bolA family member 2	3,17	3,75	FAIL
TBC1D1	H0Y8P0	TBC1 domain family member 1	3,17	5,17	FAIL
EXOC1	Q9NV70	exocyst complex component 1	3,17	3,14	FAIL
HERC5	Q9UII4	HECT and RLD domain containing E3 ubiquitin protein ligase 5	3,17	1,96	FAIL
GDI2	P50395	GDP dissociation inhibitor 2	3,16	1,53	FAIL
EXOC8	Q8IYI6	exocyst complex component 8	3,16	1,97	FAIL
BORCS6	Q96GS4	BLOC-1 related complex subunit 6	3,15	2,31	FAIL
ATP6V1B2	P21281	ATPase H+ transporting V1 subunit B2	3,15	1,84	FAIL
RAD54L2	Q9Y4B4	RAD54 like 2	3,15	3,54	FAIL
CCDC88A	A0A2R8Y7B1	coiled-coil domain containing 88A	3,15	2,03	FAIL
TBCA	O75347	tubulin folding cofactor A	3,15	1,72	FAIL
LAT	O43561	linker for activation of T cells	3,15	3,85	FAIL
DYNC1LI1	Q9Y6G9	dynein cytoplasmic 1 light intermediate chain 1	3,14	4,07	FAIL
PAFAH1B1	P43034	platelet activating factor acetylhydrolase 1b regulatory subunit 1	3,14	2,76	FAIL
FBXO22	Q8NEZ5	F-box protein 22	3,14	2,75	FAIL
PAPSS1	O43252	3'-phosphoadenosine 5'-phosphosulfate synthase 1	3,14	3,76	FAIL
HMMR	O75330	hyaluronan mediated motility receptor	3,14	2,81	FAIL
PIK3C3	A8MYT4	phosphatidylinositol 3-kinase catalytic subunit type 3	3,13	1,46	FAIL
SKA1	Q96BD8	spindle and kinetochore associated complex subunit 1	3,13	2,14	FAIL
VTA1	A0A087WY55	vesicle trafficking 1	3,13	1,30	FAIL
	A0A3F2YNW7		3,13	3,10	FAIL
WDR48	Q8TAF3	WD repeat domain 48	3,13	3,91	FAIL
EPS15L1	Q9UBC2	epidermal growth factor receptor pathway substrate 15 like 1	3,12	1,61	FAIL
ANXA6	E5RK69	annexin A6	3,12	1,97	FAIL
MAP2K1	Q02750	mitogen-activated protein kinase kinase 1	3,12	2,16	FAIL
PIP4K2A	P48426	phosphatidylinositol-5-phosphate 4-kinase type 2 alpha	3,12	1,88	FAIL
DNAJC10	Q8IXB1	DnaJ heat shock protein family (Hsp40) member C10	3,12	2,07	FAIL
NUP58	Q9BVL2	nucleoporin 58	3,11	1,99	FAIL
UBE2L3	P68036	ubiquitin conjugating enzyme E2 L3	3,11	5,06	FAIL

LAMTOR1	Q6IAA8	late endosomal/lysosomal adaptor, MAPK and MTOR activator 1	3,11	1,65	FAIL
PFAS	O15067	phosphoribosylformylglycinamidine synthase	3,11	2,91	FAIL
MACF1	H3BPE1	microtubule actin crosslinking factor 1	3,11	2,59	FAIL
ITPK1	Q13572	inositol-tetrakisphosphate 1-kinase	3,10	3,37	FAIL
CD81	E9PRJ8	CD81 molecule	3,10	1,68	FAIL
PACS1	Q6VY07	phosphofurin acidic cluster sorting protein 1	3,10	2,24	FAIL
MAPK3	E9PQW4	mitogen-activated protein kinase 3	3,09	3,40	FAIL
PISD	Q9UG56	phosphatidylserine decarboxylase	3,09	1,88	FAIL
STK38	Q15208	serine/threonine kinase 38	3,09	2,73	FAIL
C17orf49	F8W038	chromosome 17 open reading frame 49	3,09	1,52	FAIL
RAB7A	P51149	RAB7A, member RAS oncogene family	3,09	2,89	FAIL
ELP2	Q6IA86	elongator acetyltransferase complex subunit 2	3,09	3,81	FAIL
NEK9	Q8TD19	NIMA related kinase 9	3,09	1,90	FAIL
NME1	P15531	NME/NM23 nucleoside diphosphate kinase 1	3,09	2,63	FAIL
GSTP1	P09211	glutathione S-transferase pi 1	3,08	1,64	FAIL
AGO2	Q9UKV8	argonaute RISC catalytic component 2	3,08	2,26	FAIL
RFX5	F8W689	regulatory factor X5	3,08	4,18	FAIL
HSPA4	P34932	heat shock protein family A (Hsp70) member 4	3,08	2,89	FAIL
MPZL1	O95297	myelin protein zero like 1	3,08	1,81	FAIL
PIK3C2A	O00443	phosphatidylinositol-4-phosphate 3-kinase catalytic subunit type 2 alpha	3,07	1,63	FAIL
NACA	H0YHX9	nascent polypeptide associated complex subunit alpha	3,07	1,54	FAIL
PYGM	P11217	glycogen phosphorylase, muscle associated	3,07	3,27	FAIL
LDLR	J3KMZ9	low density lipoprotein receptor	3,07	2,54	FAIL
SNIP1	Q8TAD8	Smad nuclear interacting protein 1	3,06	1,36	FAIL
LARP1	Q6PKG0	La ribonucleoprotein 1, translational regulator	3,06	2,03	FAIL
BAZ1A	Q9NRL2	bromodomain adjacent to zinc finger domain 1A	3,06	3,10	FAIL
NRDC	O43847	nardilysin convertase	3,06	3,45	FAIL
HMG20A	Q9NP66	high mobility group 20A	3,06	1,68	FAIL
RABEP2	Q9H5N1	rabaptin, RAB GTPase binding effector protein 2	3,06	3,03	FAIL
SNUPN	O95149	snurportin 1	3,05	1,48	FAIL
YRDC	Q86U90	yrdC N6-threonylcarbamoyltransferase domain containing	3,05	4,93	FAIL
PSME1	Q06323	proteasome activator subunit 1	3,05	2,23	FAIL
TBC1D2B	Q9UPU7	TBC1 domain family member 2B	3,05	3,42	FAIL
MAP4K1	Q92918	mitogen-activated protein kinase kinase kinase kinase 1	3,04	2,56	FAIL
ATP6AP2	A0A1B0GTB0	ATPase H+ transporting accessory protein 2	3,04	1,81	FAIL
SLC39A6	Q13433	solute carrier family 39 member 6	3,04	2,56	FAIL
FECH	K7ELX4	ferrochelatase	3,04	1,82	FAIL
TTC4	O95801	tetratricopeptide repeat domain 4	3,04	2,21	FAIL
DDOST	P39656	dolichyl-diphosphooligosaccharide--protein glycosyltransferase non-catalytic subunit	3,04	2,85	FAIL
RPAP1	Q9BWH6	RNA polymerase II associated protein 1	3,03	1,70	FAIL
PDAP1	Q13442	PDGFA associated protein 1	3,03	3,50	FAIL
SMAD5	Q99717	SMAD family member 5	3,03	2,23	FAIL

TMEM165	Q9HC07	transmembrane protein 165	3,03	3,36	FAIL
HAUS4	Q9H6D7	HAUS augmin like complex subunit 4	3,03	3,67	FAIL
PARP9	Q8IXQ6	poly(ADP-ribose) polymerase family member 9	3,03	3,39	FAIL
SH3GLB1	Q9Y371	SH3 domain containing GRB2 like, endophilin B1	3,03	2,42	FAIL
EIF2B3	Q9NR50	eukaryotic translation initiation factor 2B subunit gamma	3,03	3,82	FAIL
RALGAPB	Q86X10	Ral GTPase activating protein non-catalytic subunit beta	3,03	2,05	FAIL
PAG1	Q9NWQ8	phosphoprotein membrane anchor with glycosphin-golipid microdomains 1	3,02	2,05	FAIL
FAM91A1	Q658Y4	family with sequence similarity 91 member A1	3,02	2,59	FAIL
HDAC1	Q13547	histone deacetylase 1	3,02	3,12	FAIL
LRCH4	O75427	leucine rich repeats and calponin homology domain containing 4	3,02	2,54	FAIL
DNPEP	E5RIA4	aspartyl aminopeptidase	3,02	2,76	FAIL
ARMC10	Q8N2F6	armadillo repeat containing 10	3,02	2,17	FAIL
ANLN	Q9NQW6	anillin, actin binding protein	3,02	1,41	FAIL
FNBP4	Q8N3X1	formin binding protein 4	3,01	2,24	FAIL
CNOT7	Q9UIV1	CCR4-NOT transcription complex subunit 7	3,01	1,56	FAIL
PPFIBP1	Q86W92	PPFIA binding protein 1	3,01	1,64	FAIL
ERI1	Q8IV48	exoribonuclease 1	3,00	1,85	FAIL
ZBTB1	Q9Y2K1	zinc finger and BTB domain containing 1	3,00	1,89	FAIL
CAPZA1	P52907	capping actin protein of muscle Z-line subunit alpha 1	3,00	2,78	FAIL
UFM1	P61960	ubiquitin fold modifier 1	3,00	4,43	FAIL
GSPT1	P15170	G1 to S phase transition 1	3,00	2,21	FAIL
GSTCD	D6R9W2	glutathione S-transferase C-terminal domain containing	3,00	1,62	FAIL
RNASEL	Q05823	ribonuclease L	3,00	1,71	FAIL
FAM117B	Q6P1L5	family with sequence similarity 117 member B	3,00	1,48	FAIL
RTCA	O00442	RNA 3'-terminal phosphate cyclase	2,99	3,59	FAIL
STRIP1	Q5VSL9	striatin interacting protein 1	2,99	3,00	FAIL
DIAPH1	O60610	diaphanous related formin 1	2,99	4,59	FAIL
CCNB2	O95067	cyclin B2	2,99	1,99	FAIL
YWHAQ	P27348	tyrosine 3-monooxygenase/tryptophan 5-monooxygen-ase activation protein theta	2,98	2,18	FAIL
THEMIS	Q8N1K5	thymocyte selection associated	2,98	1,58	FAIL
CDK5RAP3	Q96JB5	CDK5 regulatory subunit associated protein 3	2,98	1,31	FAIL
MYD88	Q99836	MYD88 innate immune signal transduction adaptor	2,98	1,44	FAIL
RB1CC1	Q8TDY2	RB1 inducible coiled-coil 1	2,98	3,83	FAIL
CAPRIN1	Q14444	cell cycle associated protein 1	2,97	2,44	FAIL
TAF4	O00268	TATA-box binding protein associated factor 4	2,97	1,46	FAIL
CAMK4	Q16566	calcium/calmodulin dependent protein kinase IV	2,96	2,09	FAIL
MAPK14	B4E0K5	mitogen-activated protein kinase 14	2,96	2,04	FAIL
EXOC6B	Q9Y2D4	exocyst complex component 6B	2,96	1,40	FAIL
VPS72	Q15906	vacuolar protein sorting 72 homolog	2,96	2,48	FAIL
CDV3	Q9UKY7	CDV3 homolog	2,96	2,50	FAIL
DOP1B	Q9Y3R5	DOP1 leucine zipper like protein B	2,96	1,70	FAIL

RRM2	P31350	ribonucleotide reductase regulatory subunit M2	2,95	4,52	FAIL
KPNA4	O00629	karyopherin subunit alpha 4	2,95	2,05	FAIL
GFPT1	Q06210	glutamine--fructose-6-phosphate transaminase 1	2,95	2,93	FAIL
DHX40	Q8IX18	DEAH-box helicase 40	2,95	1,61	FAIL
AP4M1	O00189	adaptor related protein complex 4 subunit mu 1	2,94	2,85	FAIL
PALS2	Q9NZW5	protein associated with LIN7 2, MAGUK p55 family member	2,94	3,32	FAIL
CAPG	P40121	capping actin protein, gelsolin like	2,94	1,93	FAIL
RUFY3	Q7L099	RUN and FYVE domain containing 3	2,94	3,47	FAIL
CFAP410	O43822	cilia and flagella associated protein 410	2,94	1,59	FAIL
TLK2	Q86UE8	tousled like kinase 2	2,93	3,43	FAIL
WNK1	F5GWT4	WNK lysine deficient protein kinase 1	2,93	1,98	FAIL
NAP1L4	Q99733	nucleosome assembly protein 1 like 4	2,93	3,45	FAIL
DCTN4	Q9UJW0	dynactin subunit 4	2,92	4,23	FAIL
CDC37	Q16543	cell division cycle 37, HSP90 cochaperone	2,92	4,74	FAIL
PHLDB3	Q6NSJ2	pleckstrin homology like domain family B member 3	2,92	1,86	FAIL
RAB23	Q9ULC3	RAB23, member RAS oncogene family	2,92	3,51	FAIL
	F5GYQ2		2,92	1,62	FAIL
NMRAL1	Q9HBL8	NmrA like redox sensor 1	2,92	2,32	FAIL
AFAP1	Q8N556	actin filament associated protein 1	2,91	1,71	FAIL
SLC4A7	E9PFN4	solute carrier family 4 member 7	2,91	2,30	FAIL
PFN2	P35080	profilin 2	2,90	2,15	FAIL
SLC35E1	H7C1I0	solute carrier family 35 member E1	2,90	1,64	FAIL
LIMA1	F8VQE1	LIM domain and actin binding 1	2,90	4,50	FAIL
PCBP1	Q15365	poly(rC) binding protein 1	2,90	2,19	FAIL
NDUFB7	P17568	NADH:ubiquinone oxidoreductase subunit B7	2,90	2,94	FAIL
HOMER1	Q86YM7	homer scaffold protein 1	2,90	3,12	FAIL
COPS2	P61201	COP9 signalosome subunit 2	2,90	2,08	FAIL
R3HDM1	Q15032	R3H domain containing 1	2,90	1,98	FAIL
NIPBL	A0A590UJS4	NIPBL cohesin loading factor	2,89	3,78	FAIL
CMAS	Q8NFW8	cytidine monophosphate N-acetylneuraminic acid synthetase	2,89	2,66	FAIL
RAC1	P63000	Rac family small GTPase 1	2,89	2,14	FAIL
USP4	Q13107	ubiquitin specific peptidase 4	2,89	3,75	FAIL
GNA13	Q14344	G protein subunit alpha 13	2,89	2,47	FAIL
SEC23A	F5H365	SEC23 homolog A, COPII coat complex component	2,88	1,50	FAIL
SLC12A2	P55011	solute carrier family 12 member 2	2,88	2,96	FAIL
TMEM33	D6RAA6	transmembrane protein 33	2,88	1,90	FAIL
HAT1	O14929	histone acetyltransferase 1	2,88	1,68	FAIL
WDR7	Q9Y4E6	WD repeat domain 7	2,88	1,35	FAIL
C7orf50	Q9BRJ6	chromosome 7 open reading frame 50	2,88	1,72	FAIL
TRIO	O75962	trio Rho guanine nucleotide exchange factor	2,87	2,54	FAIL
CD7	J3QLM0	CD7 molecule	2,87	1,49	FAIL
WDR75	Q8IWA0	WD repeat domain 75	2,87	1,43	FAIL
PRKAR2B	P31323	protein kinase cAMP-dependent type II regulatory subunit beta	2,87	2,53	FAIL

GIMAP6	Q6P9H5	GTPase, IMAP family member 6	2,87	1,39	FAIL
TMX3	Q96JJ7	thioredoxin related transmembrane protein 3	2,86	1,88	FAIL
ZFC3H1	O60293	zinc finger C3H1-type containing	2,86	1,67	FAIL
GRK2	P25098	G protein-coupled receptor kinase 2	2,86	3,72	FAIL
DDB1	Q16531	damage specific DNA binding protein 1	2,86	2,39	FAIL
LDHA	P00338	lactate dehydrogenase A	2,86	2,97	FAIL
PML	H3BT57	PML nuclear body scaffold	2,86	1,90	FAIL
CYCS	C9JFR7	cytochrome c, somatic	2,86	3,34	FAIL
CD82	P27701	CD82 molecule	2,86	1,77	FAIL
RALA	P11233	RAS like proto-oncogene A	2,86	1,79	FAIL
CSNK1G1	A0A0U1RQY7	casein kinase 1 gamma 1	2,85	1,85	FAIL
ZNF281	Q9Y2X9	zinc finger protein 281	2,85	2,19	FAIL
MRPS6	P82932	mitochondrial ribosomal protein S6	2,85	3,38	FAIL
PTPRA	P18433	protein tyrosine phosphatase receptor type A	2,85	2,83	FAIL
HAUS2	Q9NVX0	HAUS augmin like complex subunit 2	2,85	2,88	FAIL
EPHA3	C9JXA2	EPH receptor A3	2,85	2,45	FAIL
USP19	A0A0A0MR08	ubiquitin specific peptidase 19	2,85	4,47	FAIL
VANGL1	Q8TAA9	VANGL planar cell polarity protein 1	2,85	3,54	FAIL
NUDT11	Q96G61	nudix hydrolase 11	2,84	1,89	FAIL
TWF2	Q6IBS0	twinfilin actin binding protein 2	2,84	1,72	FAIL
RHOT1	Q8IXI2	ras homolog family member T1	2,84	1,57	FAIL
ACTR1A	P61163	actin related protein 1A	2,84	4,55	FAIL
UBR3	Q6ZT12	ubiquitin protein ligase E3 component n-recognin 3	2,84	2,19	FAIL
MTA3	E7EQY4	metastasis associated 1 family member 3	2,84	2,34	FAIL
KPNA3	O00505	karyopherin subunit alpha 3	2,83	2,12	FAIL
PLEKHA5	Q9HAU0	pleckstrin homology domain containing A5	2,83	2,13	FAIL
HEATR3	Q7Z4Q2	HEAT repeat containing 3	2,83	3,21	FAIL
RAB5C	P51148	RAB5C, member RAS oncogene family	2,83	2,90	FAIL
PRDX6	P30041	peroxiredoxin 6	2,83	3,11	FAIL
RCOR3	Q9P2K3	REST corepressor 3	2,83	1,63	FAIL
NAPA	P54920	NSF attachment protein alpha	2,83	1,47	FAIL
LANCL1	E9PHS0	LanC like glutathione S-transferase 1	2,82	2,95	FAIL
PDZD11	Q5EBL8	PDZ domain containing 11	2,82	1,54	FAIL
HCCS	P53701	holocytochrome c synthase	2,82	5,20	FAIL
LIG3	P49916	DNA ligase 3	2,82	4,25	FAIL
EXOSC6	Q5RKV6	exosome component 6	2,82	1,50	FAIL
TRAT1	Q6PIZ9	T cell receptor associated transmembrane adaptor 1	2,82	2,49	FAIL
VEZT	Q9HBM0	vezatin, adherens junctions transmembrane protein	2,82	1,77	FAIL
ARHGAP35	Q9NRY4	Rho GTPase activating protein 35	2,82	4,45	FAIL
LIMD1	C9JRJ5	LIM domain containing 1	2,82	2,53	FAIL
AGPAT1	Q99943	1-acylglycerol-3-phosphate O-acyltransferase 1	2,82	1,95	FAIL
DMXL2	H0YLM8	Dmx like 2	2,81	3,58	FAIL
ZNF318	Q5VUA4	zinc finger protein 318	2,81	1,67	FAIL
CHMP5	Q9NZZ3	charged multivesicular body protein 5	2,81	2,91	FAIL
MAGED2	Q9UNF1	MAGE family member D2	2,81	1,56	FAIL
PSME2	A0A087X1Z3	proteasome activator subunit 2	2,81	2,11	FAIL
RAB3GAP2	Q9H2M9	RAB3 GTPase activating non-catalytic protein subunit 2	2,80	3,06	FAIL

NOMO2	Q5JPE7	NODAL modulator 2	2,80	2,96	FAIL
MKI67	P46013	marker of proliferation Ki-67	2,80	2,96	FAIL
DYRK1A	A0A2R8Y6I6	dual specificity tyrosine phosphorylation regulated kinase 1A	2,80	3,97	FAIL
RUFY1	Q96T51	RUN and FYVE domain containing 1	2,80	3,11	FAIL
CNIH4	Q9P003	cornichon family AMPA receptor auxiliary protein 4	2,79	1,59	FAIL
SLC5A6	Q9Y289	solute carrier family 5 member 6	2,79	1,66	FAIL
PEX14	O75381	peroxisomal biogenesis factor 14	2,79	2,05	FAIL
H2AC6	Q93077	H2A clustered histone 6	2,79	1,53	FAIL
GMPS	P49915	guanine monophosphate synthase	2,79	2,72	FAIL
TFB1M	Q8WVM0	transcription factor B1, mitochondrial	2,78	1,42	FAIL
SEPTIN7	Q16181	septin 7	2,78	3,28	FAIL
MPRIP	A0A494BZV2	myosin phosphatase Rho interacting protein	2,78	2,22	FAIL
MRPL15	E5RIZ4	mitochondrial ribosomal protein L15	2,78	2,01	FAIL
APAF1	C9JLV4	apoptotic peptidase activating factor 1	2,78	4,15	FAIL
ROCK1	Q13464	Rho associated coiled-coil containing protein kinase 1	2,78	2,06	FAIL
CLNS1A	P54105	chloride nucleotide-sensitive channel 1A	2,77	1,63	FAIL
NUF2	Q9BZD4	NUF2 component of NDC80 kinetochore complex	2,77	1,61	FAIL
TUBA4A	P68366	tubulin alpha 4a	2,77	3,26	FAIL
CDK17	Q00537	cyclin dependent kinase 17	2,77	1,43	FAIL
ZNF598	Q86UK7	zinc finger protein 598, E3 ubiquitin ligase	2,77	2,84	FAIL
TOPBP1	Q92547	DNA topoisomerase II binding protein 1	2,77	2,02	FAIL
DHX30	H7BXY3	DExH-box helicase 30	2,77	2,47	FAIL
PPP6C	O00743	protein phosphatase 6 catalytic subunit	2,77	2,49	FAIL
MRPL44	Q9H9J2	mitochondrial ribosomal protein L44	2,77	1,37	FAIL
COPS3	H7C3P9	COP9 signalosome subunit 3	2,77	3,69	FAIL
RNF138	Q8WVD3	ring finger protein 138	2,76	1,60	FAIL
FLVCR1	Q9Y5Y0	FLVCR choline and heme transporter 1	2,76	2,00	FAIL
BRIP1	Q9BX63	BRCA1 interacting helicase 1	2,76	1,42	FAIL
PLCG2	P16885	phospholipase C gamma 2	2,76	2,11	FAIL
FARP2	O94887	FERM, ARH/RhoGEF and pleckstrin domain protein 2	2,76	2,34	FAIL
STK4	Q13043	serine/threonine kinase 4	2,76	4,34	FAIL
NDUFA13	Q9P0J0	NADH:ubiquinone oxidoreductase subunit A13	2,76	2,54	FAIL
TARS3	A2RTX5	threonyl-tRNA synthetase 3	2,76	1,44	FAIL
CBL	P22681	Cbl proto-oncogene	2,76	2,02	FAIL
DTL	Q9NZJ0	denticleless E3 ubiquitin protein ligase homolog	2,76	3,19	FAIL
ZEB1	P37275	zinc finger E-box binding homeobox 1	2,76	1,96	FAIL
CASP8AP2	Q9UKL3	caspase 8 associated protein 2	2,76	1,34	FAIL
PPP4R3B	Q5MIZ7	protein phosphatase 4 regulatory subunit 3B	2,76	1,52	FAIL
HOOK3	Q86VS8	hook microtubule tethering protein 3	2,75	1,45	FAIL
MED4	Q9NPJ6	mediator complex subunit 4	2,75	1,87	FAIL
NBEAL2	A0A494C1V1	neurobeachin like 2	2,75	3,33	FAIL
CSK	P41240	C-terminal Src kinase	2,75	3,08	FAIL
PATL1	Q86TB9	PAT1 homolog 1, processing body mRNA decay factor	2,75	2,78	FAIL
DAXX	Q9UER7	death domain associated protein	2,75	2,12	FAIL
CARM1	Q86X55	coactivator associated arginine methyltransferase 1	2,75	2,62	FAIL
SMG7	E9PD50	SMG7 nonsense mediated mRNA decay factor	2,75	2,58	FAIL

RUSF1	H3BR29	RUS family member 1	2,75	1,69	FAIL
SPAG9	O60271	sperm associated antigen 9	2,75	3,42	FAIL
CYTH1	Q15438	cytohesin 1	2,75	2,50	FAIL
UHRF2	Q96PU4	ubiquitin like with PHD and ring finger domains 2	2,74	3,25	FAIL
NCOA3	Q9Y6Q9	nuclear receptor coactivator 3	2,74	3,78	FAIL
	A6NML8		2,74	1,83	FAIL
NAA16	Q6N069	N-alpha-acetyltransferase 16, NatA auxiliary subunit	2,74	1,90	FAIL
DYNC1LI2	O43237	dynein cytoplasmic 1 light intermediate chain 2	2,74	1,37	FAIL
MSN	P26038	moesin	2,74	2,57	FAIL
CCNY	Q8ND76	cyclin Y	2,73	3,59	FAIL
PRDX1	Q06830	peroxiredoxin 1	2,73	2,10	FAIL
IRF3	Q14653	interferon regulatory factor 3	2,73	1,70	FAIL
CAMLG	P49069	calcium modulating ligand	2,73	3,78	FAIL
SPPL2C	Q8IUH8	signal peptide peptidase like 2C	2,73	1,49	FAIL
MTF2	Q9Y483	metal response element binding transcription factor 2	2,73	2,31	FAIL
PPP1R12A	F8VZN8	protein phosphatase 1 regulatory subunit 12A	2,73	1,60	FAIL
GET4	Q7L5D6	guided entry of tail-anchored proteins factor 4	2,73	1,48	FAIL
NDUFB6	A0A087WZX2	NADH:ubiquinone oxidoreductase subunit B6	2,73	1,34	FAIL
ANAPC1	Q9H1A4	anaphase promoting complex subunit 1	2,73	1,69	FAIL
RAE1	P78406	ribonucleic acid export 1	2,73	1,68	FAIL
TIPRL	O75663	TOR signaling pathway regulator	2,73	3,21	FAIL
POLA1	A6NMQ1	DNA polymerase alpha 1, catalytic subunit	2,73	1,72	FAIL
PIH1D1	Q9NWS0	PIH1 domain containing 1	2,73	2,81	FAIL
	Q9ULM6		2,72	2,73	FAIL
ARHGEF7	A0A2R8YG42	Rho guanine nucleotide exchange factor 7	2,72	4,65	FAIL
KIF15	Q9NS87	kinesin family member 15	2,72	1,68	FAIL
WDR1	O75083	WD repeat domain 1	2,72	3,82	FAIL
NR2C2	P49116	nuclear receptor subfamily 2 group C member 2	2,72	2,15	FAIL
TRAPPC2L	A0A0B4J286	trafficking protein particle complex subunit 2L	2,72	1,59	FAIL
PLEKHA1	Q5RGS4	pleckstrin homology domain containing A1	2,72	2,23	FAIL
LSM3	P62310	LSM3 homolog, U6 small nuclear RNA and mRNA degradation associated	2,71	1,64	FAIL
JAM3	Q9BX67	junctional adhesion molecule 3	2,71	2,65	FAIL
RAB3GAP1	Q15042	RAB3 GTPase activating protein catalytic subunit 1	2,71	1,56	FAIL
TSR1	Q2NL82	TSR1 ribosome maturation factor	2,71	1,59	FAIL
GADD45GIP1	Q8TAE8	GADD45G interacting protein 1	2,71	1,76	FAIL
CTNNB1	A0A2R8YCH5	catenin beta 1	2,71	2,20	FAIL
REEP5	Q00765	receptor accessory protein 5	2,71	2,54	FAIL
AMMECR1L	Q6DCA0	AMMECR1 like	2,71	2,89	FAIL
TPP2	A0A494C0U1	tripeptidyl peptidase 2	2,71	2,18	FAIL
CNOT11	Q9UKZ1	CCR4-NOT transcription complex subunit 11	2,71	1,70	FAIL
ACAP2	Q15057	ArfGAP with coiled-coil, ankyrin repeat and PH domains 2	2,71	2,80	FAIL
RAB18	Q9NP72	RAB18, member RAS oncogene family	2,71	2,20	FAIL
VCP	P55072	valosin containing protein	2,71	1,98	FAIL
NT5C3A	X6RM59	5'-nucleotidase, cytosolic IIIA	2,71	4,27	FAIL
GCLM	P48507	glutamate-cysteine ligase modifier subunit	2,71	2,83	FAIL

RFXAP	O00287	regulatory factor X associated protein	2,70	2,61	FAIL
PPP2R5D	E9PFR3	protein phosphatase 2 regulatory subunit B'delta	2,70	2,69	FAIL
ITGB2	P05107	integrin subunit beta 2	2,70	1,52	FAIL
COG1	Q8WTW3	component of oligomeric golgi complex 1	2,70	2,96	FAIL
GIT2	Q14161	GIT ArfGAP 2	2,70	2,38	FAIL
ELP1	O95163	elongator acetyltransferase complex subunit 1	2,70	3,37	FAIL
	A0A1W2PNZ9		2,70	5,48	FAIL
SUDS3	Q9H7L9	SDS3 homolog, SIN3A corepressor complex component	2,70	2,11	FAIL
AGAP2	F8VVT9	ArfGAP with GTPase domain, ankyrin repeat and PH domain 2	2,70	4,53	FAIL
MAT2B	Q9NZL9	methionine adenosyltransferase 2 non-catalytic beta subunit	2,69	1,97	FAIL
ETS1	P14921	ETS proto-oncogene 1, transcription factor	2,69	6,10	FAIL
LYPLAL1	Q5VWZ2	lysophospholipase like 1	2,69	2,16	FAIL
NAF1	Q96HR8	nuclear assembly factor 1 ribonucleoprotein	2,69	1,94	FAIL
BUB1B	O60566	BUB1 mitotic checkpoint serine/threonine kinase B	2,69	1,82	FAIL
SQLE	Q14534	squalene epoxidase	2,69	1,33	FAIL
AGPAT2	O15120	1-acylglycerol-3-phosphate O-acyltransferase 2	2,69	1,73	FAIL
TAPT1	Q6NXT6	transmembrane anterior posterior transformation 1	2,68	3,18	FAIL
UBE2I	H3BPC4	ubiquitin conjugating enzyme E2 I	2,68	1,49	FAIL
THEM6	Q8WUY1	thioesterase superfamily member 6	2,68	4,30	FAIL
ITPR2	Q14571	inositol 1,4,5-trisphosphate receptor type 2	2,68	3,73	FAIL
TPD52L2	O43399	TPD52 like 2	2,67	1,98	FAIL
IRAG2	F8W9L6	inositol 1,4,5-triphosphate receptor associated 2	2,67	2,23	FAIL
KANSL3	Q9P2N6	KAT8 regulatory NSL complex subunit 3	2,67	1,74	FAIL
RNF40	O75150	ring finger protein 40	2,67	5,61	FAIL
DEPDC1B	Q8WUY9	DEP domain containing 1B	2,67	4,67	FAIL
CD2AP	Q9Y5K6	CD2 associated protein	2,67	1,88	FAIL
PSMD2	Q13200	proteasome 26S subunit ubiquitin receptor, non-ATPase 2	2,67	2,37	FAIL
KARS1	Q15046	lysyl-tRNA synthetase 1	2,67	3,34	FAIL
CMPK1	P30085	cytidine/uridine monophosphate kinase 1	2,67	2,30	FAIL
ANKHD1	D6RHC4	ankyrin repeat and KH domain containing 1	2,67	1,77	FAIL
ARL6IP6	Q8N6S5	ADP ribosylation factor like GTPase 6 interacting protein 6	2,67	1,64	FAIL
NCAPG2	Q86XI2	non-SMC condensin II complex subunit G2	2,66	1,89	FAIL
EIF1	P41567	eukaryotic translation initiation factor 1	2,66	1,52	FAIL
SHCBP1	Q8NEM2	SHC binding and spindle associated 1	2,66	1,41	FAIL
GIMAP4	Q9NUV9	GTPase, IMAP family member 4	2,66	1,61	FAIL
EXOSC7	Q15024	exosome component 7	2,66	2,07	FAIL
AHDC1	Q5TGY3	AT-hook DNA binding motif containing 1	2,66	1,58	FAIL
DBN1	Q16643	drebrin 1	2,66	4,29	FAIL
PRRC2B	Q5JSZ5	proline rich coiled-coil 2B	2,66	1,76	FAIL
SLC4A2	P04920	solute carrier family 4 member 2	2,66	2,08	FAIL
GIT1	A0A0C4DGN6	GIT ArfGAP 1	2,66	4,42	FAIL
MTHFD2	P13995	methylenetetrahydrofolate dehydrogenase (NADP+ dependent) 2, methenyltetrahydrofolate cyclohydrolase	2,66	1,39	FAIL

EIF3G	O75821	eukaryotic translation initiation factor 3 subunit G	2,65	3,88	FAIL
NGLY1	A0A0C4DFP4	N-glycanase 1	2,65	1,56	FAIL
MEN1	A0A5F9ZHS3	menin 1	2,65	3,98	FAIL
LARP1B	A0A3B3ISF0	La ribonucleoprotein 1B	2,65	1,87	FAIL
ARRB2	P32121	arrestin beta 2	2,65	2,56	FAIL
STK38L	Q9Y2H1	serine/threonine kinase 38 like	2,65	2,91	FAIL
GNAQ	P50148	G protein subunit alpha q	2,65	4,23	FAIL
MTMR1	F8WA39	myotubularin related protein 1	2,64	1,89	FAIL
PPM1A	P35813	protein phosphatase, Mg2+/Mn2+ dependent 1A	2,64	1,80	FAIL
LTA4H	P09960	leukotriene A4 hydrolase	2,64	2,65	FAIL
PRRC2C	Q9Y520	proline rich coiled-coil 2C	2,64	4,87	FAIL
LRRFIP1	Q32MZ4	LRR binding FLII interacting protein 1	2,64	2,52	FAIL
TCP1	P17987	t-complex 1	2,64	3,00	FAIL
UBXN1	Q04323	UBX domain protein 1	2,64	2,47	FAIL
ESYT1	Q9BSJ8	extended synaptotagmin 1	2,63	3,22	FAIL
CNOT9	Q92600	CCR4-NOT transcription complex subunit 9	2,63	2,64	FAIL
ATP6V1A	P38606	ATPase H+ transporting V1 subunit A	2,63	2,87	FAIL
EIF3J	O75822	eukaryotic translation initiation factor 3 subunit J	2,63	3,48	FAIL
DICER1	Q9UPY3	dicer 1, ribonuclease III	2,63	2,07	FAIL
NR3C1	P04150	nuclear receptor subfamily 3 group C member 1	2,63	2,14	FAIL
SLFN5	Q08AF3	schlafen family member 5	2,63	1,47	FAIL
PPP3CB	P16298	protein phosphatase 3 catalytic subunit beta	2,63	6,03	FAIL
CHMP4B	Q9H444	charged multivesicular body protein 4B	2,62	1,76	FAIL
ATP2A3	Q93084	ATPase sarcoplasmic/endoplasmic reticulum Ca2+ transporting 3	2,62	2,29	FAIL
USP47	Q96K76	ubiquitin specific peptidase 47	2,62	2,19	FAIL
SHOC2	Q9UQ13	SHOC2 leucine rich repeat scaffold protein	2,62	1,74	FAIL
UBASH3B	Q8TF42	ubiquitin associated and SH3 domain containing B	2,61	1,50	FAIL
SKP1	P63208	S-phase kinase associated protein 1	2,61	1,50	FAIL
KANSL1	Q7Z3B3	KAT8 regulatory NSL complex subunit 1	2,61	1,51	FAIL
BEND3	Q5T5X7	BEN domain containing 3	2,61	1,50	FAIL
PPP6R1	Q9UPN7	protein phosphatase 6 regulatory subunit 1	2,61	2,33	FAIL
TBL2	Q9Y4P3	transducin beta like 2	2,61	2,30	FAIL
VPS11	Q9H270	VPS11 core subunit of CORVET and HOPS complexes	2,61	2,46	FAIL
SEC63	Q9UGP8	SEC63 homolog, protein translocation regulator	2,60	2,98	FAIL
CHD7	Q9P2D1	chromodomain helicase DNA binding protein 7	2,60	2,92	FAIL
ARL6IP5	O75915	ADP ribosylation factor like GTPase 6 interacting protein 5	2,60	1,67	FAIL
STAM2	O75886	signal transducing adaptor molecule 2	2,60	3,55	FAIL
PPP1CA	P62136	protein phosphatase 1 catalytic subunit alpha	2,60	3,09	FAIL
RNH1	P13489	ribonuclease/angiogenin inhibitor 1	2,60	2,74	FAIL
RBM19	Q9Y4C8	RNA binding motif protein 19	2,60	1,69	FAIL
STX4	Q12846	syntaxin 4	2,60	1,58	FAIL
DCAF5	Q96JK2	DDB1 and CUL4 associated factor 5	2,60	1,47	FAIL
GSTO1	P78417	glutathione S-transferase omega 1	2,60	2,74	FAIL
DEGS1	O15121	delta 4-desaturase, sphingolipid 1	2,59	2,62	FAIL

BMP2K	Q9NSY1	BMP2 inducible kinase	2,59	3,72	FAIL
PYGB	P11216	glycogen phosphorylase B	2,59	2,31	FAIL
RAI1	Q7Z5J4	retinoic acid induced 1	2,59	2,74	FAIL
UPF3B	Q9BZI7	UPF3B regulator of nonsense mediated mRNA decay	2,59	1,93	FAIL
ETV6	P41212	ETS variant transcription factor 6	2,59	2,14	FAIL
NME7	Q9Y5B8	NME/NM23 family member 7	2,59	2,43	FAIL
MOB1B	Q7L9L4	MOB kinase activator 1B	2,59	1,87	FAIL
GPN1	Q9HCN4	GPN-loop GTPase 1	2,59	1,45	FAIL
SRC	P12931	SRC proto-oncogene, non-receptor tyrosine kinase	2,59	2,39	FAIL
WDR18	Q9BV38	WD repeat domain 18	2,59	2,81	FAIL
TNRC6B	Q9UPQ9	trinucleotide repeat containing adaptor 6B	2,58	2,43	FAIL
ZNF106	Q9H2Y7	zinc finger protein 106	2,58	4,37	FAIL
UBE4B	O95155	ubiquitination factor E4B	2,58	5,05	FAIL
PHC3	Q8NDX5	polyhomeotic homolog 3	2,58	3,56	FAIL
UBFD1	O14562	ubiquitin family domain containing 1	2,58	3,63	FAIL
SPTLC2	O15270	serine palmitoyltransferase long chain base subunit 2	2,58	1,93	FAIL
KHSRP	Q92945	KH-type splicing regulatory protein	2,58	2,50	FAIL
ACBD6	Q9BR61	acyl-CoA binding domain containing 6	2,58	1,78	FAIL
PRKAR2A	P13861	protein kinase cAMP-dependent type II regulatory sub-unit alpha	2,57	3,83	FAIL
ORC3	Q9UBD5	origin recognition complex subunit 3	2,57	3,39	FAIL
XPO4	Q9C0E2	exportin 4	2,57	1,35	FAIL
CYFIP1	Q7L576	cytoplasmic FMR1 interacting protein 1	2,57	2,24	FAIL
SLC19A1	P41440	solute carrier family 19 member 1	2,57	2,19	FAIL
PSMD5	Q16401	proteasome 26S subunit, non-ATPase 5	2,57	1,70	FAIL
ILK	A0A0A0MTH3	integrin linked kinase	2,57	1,74	FAIL
BTN3A2	S4R3N0	butyrophilin subfamily 3 member A2	2,56	1,39	FAIL
PSMC1	P62191	proteasome 26S subunit, ATPase 1	2,56	2,89	FAIL
EXOC7	A0A0A0MSB8	exocyst complex component 7	2,56	3,08	FAIL
SCD5	Q86SK9	stearoyl-CoA desaturase 5	2,56	1,74	FAIL
PPM1L	Q5SGD2	protein phosphatase, Mg2+/Mn2+ dependent 1L	2,56	1,96	FAIL
GIPC1	O14908	GIPC PDZ domain containing family member 1	2,56	3,02	FAIL
HTATSF1	O43719	HIV-1 Tat specific factor 1	2,56	3,95	FAIL
PITPNB	P48739	phosphatidylinositol transfer protein beta	2,56	2,04	FAIL
UBE2O	Q9C0C9	ubiquitin conjugating enzyme E2 O	2,55	2,37	FAIL
SLC1A4	P43007	solute carrier family 1 member 4	2,55	2,63	FAIL
FPGS	Q05932	folylpolyglutamate synthase	2,55	1,58	FAIL
CCDC61	Q9Y6R9	coiled-coil domain containing 61	2,55	5,22	FAIL
SCD	O00767	stearoyl-CoA desaturase	2,55	1,72	FAIL
NUDT1	P36639	nudix hydrolase 1	2,55	2,16	FAIL
TYMS	P04818	thymidylate synthetase	2,55	1,69	FAIL
ELL	P55199	elongation factor for RNA polymerase II	2,55	1,35	FAIL
AFF4	Q9UHB7	ALF transcription elongation factor 4	2,54	2,02	FAIL
ZNF217	O75362	zinc finger protein 217	2,54	2,31	FAIL
PRKCI	P41743	protein kinase C iota	2,54	1,42	FAIL
ATP6V1F	Q16864	ATPase H+ transporting V1 subunit F	2,54	4,69	FAIL

EIF4EBP2	Q13542	eukaryotic translation initiation factor 4E binding protein 2	2,54	1,64	FAIL
ZNF592	H0YM74	zinc finger protein 592	2,54	1,81	FAIL
SGO2	Q562F6	shugoshin 2	2,54	1,40	FAIL
SORT1	Q99523	sortilin 1	2,54	2,45	FAIL
STK33	Q9BYT3	serine/threonine kinase 33	2,54	1,53	FAIL
ELMOD2	Q8IZ81	ELMO domain containing 2	2,54	4,73	FAIL
RIMOC1	A6NDU8	RAB7A interacting MON1-CCZ1 complex subunit 1	2,53	3,00	FAIL
PHF8	H0Y3N9	PHD finger protein 8	2,53	4,80	FAIL
AURKB	Q96GD4	aurora kinase B	2,53	2,72	FAIL
IQGAP1	P46940	IQ motif containing GTPase activating protein 1	2,53	2,37	FAIL
ITGB1	P05556	integrin subunit beta 1	2,53	3,60	FAIL
NOTCH1	P46531	notch receptor 1	2,53	1,56	FAIL
PYCR3	Q53H96	pyrroline-5-carboxylate reductase 3	2,52	1,33	FAIL
STING1	Q86WV6	stimulator of interferon response cGAMP interactor 1	2,52	1,43	FAIL
CDAN1	Q8IWY9	codanin 1	2,52	3,49	FAIL
PRDM9	Q9NQV7	PR/SET domain 9	2,52	2,90	FAIL
CUL5	Q93034	cullin 5	2,52	2,07	FAIL
SRP9	Q6P2S0	signal recognition particle 9	2,52	1,63	FAIL
TSFM	P43897	Ts translation elongation factor, mitochondrial	2,52	1,34	FAIL
PCM1	E7EV93	pericentriolar material 1	2,51	2,30	FAIL
GRPEL1	Q9HAV7	GrpE like 1, mitochondrial	2,51	1,34	FAIL
	B3KNS4		2,51	2,42	FAIL
SLC12A7	Q9Y666	solute carrier family 12 member 7	2,51	1,90	FAIL
CCT8	P50990	chaperonin containing TCP1 subunit 8	2,51	3,56	FAIL
PHF10	Q8WUB8	PHD finger protein 10	2,51	1,48	FAIL
IMPDH2	H0Y4R1	inosine monophosphate dehydrogenase 2	2,51	2,45	FAIL
SCML2	Q9UQR0	Scm polycomb group protein like 2	2,51	1,62	FAIL
MPHOSPH10	O00566	M-phase phosphoprotein 10	2,51	1,79	FAIL
CNOT2	F8VV52	CCR4-NOT transcription complex subunit 2	2,50	1,73	FAIL
GAPVD1	Q14C86	GTPase activating protein and VPS9 domains 1	2,50	4,05	FAIL
CPOX	P36551	coproporphyrinogen oxidase	2,50	1,67	FAIL
SMNDC1	O75940	survival motor neuron domain containing 1	2,50	2,14	FAIL
NOL8	Q76FK4	nucleolar protein 8	2,50	2,44	FAIL
RICTOR	Q6R327	RPTOR independent companion of MTOR complex 2	2,50	2,04	FAIL
PEX5	P50542	peroxisomal biogenesis factor 5	2,50	2,51	FAIL
ADRM1	Q16186	ADRM1 26S proteasome ubiquitin receptor	2,50	1,56	FAIL
ICAM3	P32942	intercellular adhesion molecule 3	2,49	4,27	FAIL
CGGBP1	Q9UFW8	CGG triplet repeat binding protein 1	2,49	1,49	FAIL
PAIP2	Q9BPZ3	poly(A) binding protein interacting protein 2	2,49	3,23	FAIL
ISG15	A0A096LNZ9	ISG15 ubiquitin like modifier	2,49	3,82	FAIL
	A0A087WUX8		2,49	2,84	FAIL
BCR	P11274	BCR activator of RhoGEF and GTPase	2,49	2,45	FAIL
CD3G	P09693	CD3 gamma subunit of T-cell receptor complex	2,48	1,97	FAIL
RAP2B	P61225	RAP2B, member of RAS oncogene family	2,48	3,13	FAIL
ZC3HAV1L	Q96H79	zinc finger CCCH-type containing, antiviral 1 like	2,48	1,82	FAIL
PXDN	Q92626	peroxidasin	2,48	1,54	FAIL
EPS15	P42566	epidermal growth factor receptor pathway substrate 15	2,48	1,72	FAIL

SHC1	P29353	SHC adaptor protein 1	2,48	6,87	FAIL
GID8	Q9NWU2	GID complex subunit 8 homolog	2,48	1,89	FAIL
CALM2	P0DP25	calmodulin 2	2,47	2,20	FAIL
M6PR	P20645	mannose-6-phosphate receptor, cation dependent	2,47	1,52	FAIL
PDXDC1	Q6P996	pyridoxal dependent decarboxylase domain containing 1	2,47	1,37	FAIL
PREB	Q9HCU5	prolactin regulatory element binding	2,46	2,22	FAIL
SAAL1	J3KND1	serum amyloid A like 1	2,46	2,98	FAIL
MUS81	H0YDE3	MUS81 structure-specific endonuclease subunit	2,46	3,40	FAIL
HSPBP1	Q9NZL4	HSPA (Hsp70) binding protein 1	2,46	2,53	FAIL
CETN2	P41208	centrin 2	2,46	3,73	FAIL
MRPL4	K7ELF1	mitochondrial ribosomal protein L4	2,46	1,86	FAIL
POLR3B	Q9NW08	RNA polymerase III subunit B	2,46	2,61	FAIL
SCFD2	Q8WU76	sec1 family domain containing 2	2,46	2,10	FAIL
TXNRD1	Q16881	thioredoxin reductase 1	2,46	1,98	FAIL
VPS37A	Q8NEZ2	VPS37A subunit of ESCRT-I	2,45	3,06	FAIL
ELAC2	Q9BQ52	elaC ribonuclease Z 2	2,45	1,78	FAIL
KIDINS220	Q9ULH0	kinase D interacting substrate 220	2,45	2,96	FAIL
NEDD4L	A0A6Q8PFI7	NEDD4 like E3 ubiquitin protein ligase	2,45	2,00	FAIL
DMAP1	Q9NPF5	DNA methyltransferase 1 associated protein 1	2,45	1,52	FAIL
RPA3	B5MC59	replication protein A3	2,45	1,67	FAIL
FYN	P06241	FYN proto-oncogene, Src family tyrosine kinase	2,45	2,89	FAIL
TNPO2	O14787	transportin 2	2,45	1,40	FAIL
PPM1G	O15355	protein phosphatase, Mg2+/Mn2+ dependent 1G	2,45	3,83	FAIL
DPP9	Q86TI2	dipeptidyl peptidase 9	2,45	3,38	FAIL
TXNL1	K7ER96	thioredoxin like 1	2,44	1,90	FAIL
YEATS4	F8W0J4	YEATS domain containing 4	2,44	4,07	FAIL
RTN4	Q9NQC3	reticulon 4	2,44	2,25	FAIL
RPIA	P49247	ribose 5-phosphate isomerase A	2,44	4,24	FAIL
DDX59	Q5T1V6	DEAD-box helicase 59	2,44	2,51	FAIL
PDCD2L	Q9BRP1	programmed cell death 2 like	2,44	1,65	FAIL
HLA-C	A0A140T9H5	major histocompatibility complex, class I, C	2,44	3,83	FAIL
PIGK	Q92643	phosphatidylinositol glycan anchor biosynthesis class K	2,44	1,99	FAIL
POLR2J	P52435	RNA polymerase II subunit J	2,44	1,90	FAIL
BRD7	Q9NPI1	bromodomain containing 7	2,44	1,67	FAIL
RNF214	Q8ND24	ring finger protein 214	2,44	3,36	FAIL
FAM98B	Q52LJ0	family with sequence similarity 98 member B	2,44	2,07	FAIL
NDUFB3	O43676	NADH:ubiquinone oxidoreductase subunit B3	2,43	1,96	FAIL
MBLAC2	Q68D91	metallo-beta-lactamase domain containing 2	2,43	2,92	FAIL
WAPL	Q7Z5K2	WAPL cohesin release factor	2,43	2,81	FAIL
LRCH3	Q96II8	leucine rich repeats and calponin homology domain containing 3	2,43	2,20	FAIL
ABI3	Q9P2A4	ABI family member 3	2,43	3,04	FAIL
OSTF1	Q92882	osteoclast stimulating factor 1	2,43	2,16	FAIL
INTS2	J3KMZ7	integrator complex subunit 2	2,43	1,59	FAIL
TNRC6C	Q9HCJ0	trinucleotide repeat containing adaptor 6C	2,43	2,22	FAIL
VPS26C	A8MTY9	VPS26 endosomal protein sorting factor C	2,43	1,39	FAIL

GLRX3	O76003	glutaredoxin 3	2,42	4,05	FAIL
EIF4H	Q15056	eukaryotic translation initiation factor 4H	2,42	2,84	FAIL
DGKH	Q86XP1	diacylglycerol kinase eta	2,42	1,91	FAIL
GNAI3	P08754	G protein subunit alpha i3	2,42	2,57	FAIL
PDCD6IP	F8WBR8	programmed cell death 6 interacting protein	2,42	2,41	FAIL
PABPC1	A0A087WTT1	poly(A) binding protein cytoplasmic 1	2,42	2,11	FAIL
SNX27	Q96L92	sorting nexin 27	2,42	2,19	FAIL
ENDOD1	O94919	endonuclease domain containing 1	2,42	1,56	FAIL
DDX20	Q9UHI6	DEAD-box helicase 20	2,42	1,97	FAIL
	Q32Q12		2,41	1,93	FAIL
CAB39	Q9Y376	calcium binding protein 39	2,41	1,47	FAIL
DLGAP5	Q15398	DLG associated protein 5	2,41	2,24	FAIL
ITPR1	A0A3F2YNW8	inositol 1,4,5-trisphosphate receptor type 1	2,41	1,49	FAIL
TRIP4	H0YL91	thyroid hormone receptor interactor 4	2,41	1,70	FAIL
TULP3	O75386	TUB like protein 3	2,41	1,99	FAIL
SEC23B	Q15437	SEC23 homolog B, COPII coat complex component	2,41	2,05	FAIL
DENR	O43583	density regulated re-initiation and release factor	2,41	2,33	FAIL
CNDP2	Q96KP4	carnosine dipeptidase 2	2,41	2,56	FAIL
KNTC1	P50748	kinetochore associated 1	2,40	2,92	FAIL
PAXIP1	Q6ZW49	PAX interacting protein 1	2,40	1,48	FAIL
HMGXB4	B0QXZ9	HMG-box containing 4	2,40	1,92	FAIL
	A0A0J9YWL0		2,40	1,91	FAIL
NDRG3	Q5TH30	NDRG family member 3	2,40	1,66	FAIL
MGST3	O14880	microsomal glutathione S-transferase 3	2,40	1,33	FAIL
GPR89B	P0CG08	G protein-coupled receptor 89B	2,40	2,13	FAIL
TOMM22	Q9NS69	translocase of outer mitochondrial membrane 22	2,40	1,42	FAIL
MDC1	Q14676	mediator of DNA damage checkpoint 1	2,40	3,30	FAIL
HNRNPLL	Q8WVV9	heterogeneous nuclear ribonucleoprotein L like	2,40	2,04	FAIL
PTPN6	P29350	protein tyrosine phosphatase non-receptor type 6	2,40	2,18	FAIL
INTS1	Q8N201	integrator complex subunit 1	2,40	1,59	FAIL
NSMCE3	Q96MG7	NSE3 homolog, SMC5-SMC6 complex component	2,40	2,39	FAIL
ZZEF1	O43149	zinc finger ZZ-type and EF-hand domain containing 1	2,40	2,41	FAIL
STARD3NL	O95772	STARD3 N-terminal like	2,40	1,76	FAIL
IGF2BP3	O00425	insulin like growth factor 2 mRNA binding protein 3	2,39	2,50	FAIL
CLASP2	E3W994	cytoplasmic linker associated protein 2	2,39	2,29	FAIL
ZGPAT	Q8N5A5	zinc finger CCCH-type and G-patch domain containing	2,39	1,49	FAIL
GON4L	Q3T8J9	gon-4 like	2,39	2,79	FAIL
ALG5	Q9Y673	ALG5 dolichyl-phosphate beta-glucosyltransferase	2,38	1,64	FAIL
FBXO6	Q9NRD1	F-box protein 6	2,38	1,93	FAIL
NCKAP1	Q9Y2A7	NCK associated protein 1	2,38	2,46	FAIL
CCT2	P78371	chaperonin containing TCP1 subunit 2	2,38	2,51	FAIL
ZNF207	X6R4W8	zinc finger protein 207	2,38	1,80	FAIL
FEN1	P39748	flap structure-specific endonuclease 1	2,37	1,84	FAIL
SMC6	Q96SB8	structural maintenance of chromosomes 6	2,37	1,57	FAIL
TTK	P33981	TTK protein kinase	2,37	1,97	FAIL
SEC23IP	Q9Y6Y8	SEC23 interacting protein	2,37	1,51	FAIL
ACTB	P60709	actin beta	2,37	2,80	FAIL
MRPS31	Q92665	mitochondrial ribosomal protein S31	2,37	1,32	FAIL

LDHB	A0A5F9ZHM4	lactate dehydrogenase B	2,37	3,73	FAIL
SMCR8	Q8TEV9	SMCR8-C9orf72 complex subunit	2,37	1,93	FAIL
MRPL39	C9JG87	mitochondrial ribosomal protein L39	2,37	1,94	FAIL
SLC25A24	Q6NUK1	solute carrier family 25 member 24	2,37	3,48	FAIL
AHCYL1	O43865	adenosylhomocysteinase like 1	2,37	2,34	FAIL
CPSF1	Q10570	cleavage and polyadenylation specific factor 1	2,37	3,13	FAIL
GBF1	Q92538	golgi brefeldin A resistant guanine nucleotide exchange factor 1	2,37	1,55	FAIL
SNAP29	O95721	synaptosome associated protein 29	2,36	2,94	FAIL
BMS1	Q14692	BMS1 ribosome biogenesis factor	2,36	1,76	FAIL
NDE1	A0A6Q8PGV6	nudE neurodevelopment protein 1	2,36	3,38	FAIL
CYB5B	H3BUX2	cytochrome b5 type B	2,36	1,48	FAIL
GOLGA4	H0Y6I0	golgin A4	2,36	2,92	FAIL
PHKB	I3L213	phosphorylase kinase regulatory subunit beta	2,36	3,81	FAIL
PPP1R12C	Q9BZL4	protein phosphatase 1 regulatory subunit 12C	2,35	1,69	FAIL
HEXIM1	O94992	HEXIM P-TEFb complex subunit 1	2,35	2,11	FAIL
TRAPPC9	Q96Q05	trafficking protein particle complex subunit 9	2,35	3,08	FAIL
NCKAP1L	P55160	NCK associated protein 1 like	2,35	4,21	FAIL
LCMT1	Q9UIC8	leucine carboxyl methyltransferase 1	2,35	1,42	FAIL
MAP4K2	Q12851	mitogen-activated protein kinase kinase kinase kinase 2	2,35	1,60	FAIL
FAM111B	Q6SJ93	FAM111 trypsin like peptidase B	2,35	1,34	FAIL
NDC1	Q9BTX1	NDC1 transmembrane nucleoporin	2,35	1,32	FAIL
HNRNPUL1	Q9BUJ2	heterogeneous nuclear ribonucleoprotein U like 1	2,35	2,73	FAIL
POLR2H	C9JLU1	RNA polymerase II, I and III subunit H	2,35	1,69	FAIL
SH3GLB2	B7ZC38	SH3 domain containing GRB2 like, endophilin B2	2,35	1,76	FAIL
CYP20A1	Q6UW02	cytochrome P450 family 20 subfamily A member 1	2,35	1,97	FAIL
PEAK1	Q9H792	pseudopodium enriched atypical kinase 1	2,34	2,72	FAIL
GTSE1	Q9NYZ3	G2 and S-phase expressed 1	2,34	1,71	FAIL
CC2D1A	Q6P1N0	coiled-coil and C2 domain containing 1A	2,34	2,62	FAIL
USP9Y	O00507	ubiquitin specific peptidase 9 Y-linked	2,34	2,16	FAIL
FBXL12	Q9NXK8	F-box and leucine rich repeat protein 12	2,34	1,86	FAIL
HAUS3	Q68CZ6	HAUS augmin like complex subunit 3	2,34	3,40	FAIL
TNPO1	Q92973	transportin 1	2,34	1,45	FAIL
RAPGEF5	A8MQ07	Rap guanine nucleotide exchange factor 5	2,34	2,62	FAIL
CIZ1	F5H2X7	CDKN1A interacting zinc finger protein 1	2,34	2,02	FAIL
BUB1	O43683	BUB1 mitotic checkpoint serine/threonine kinase	2,34	2,03	FAIL
OSBPL3	Q9H4L5	oxysterol binding protein like 3	2,34	2,30	FAIL
UBA1	P22314	ubiquitin like modifier activating enzyme 1	2,34	2,94	FAIL
YES1	J3QRU1	YES proto-oncogene 1, Src family tyrosine kinase	2,34	2,45	FAIL
KAT6A	A0A3F2YNX6	lysine acetyltransferase 6A	2,33	2,21	FAIL
MAVS	Q7Z434	mitochondrial antiviral signaling protein	2,33	3,03	FAIL
N4BP2	Q86UW6	NEDD4 binding protein 2	2,33	3,17	FAIL
SCAMP3	O14828	secretory carrier membrane protein 3	2,33	2,45	FAIL
GTF3C4	Q9UKN8	general transcription factor IIIC subunit 4	2,33	2,70	FAIL
AP5B1	Q2VPB7	adaptor related protein complex 5 subunit beta 1	2,33	1,33	FAIL
FARS2	A0A3B3ITR6	phenylalanyl-tRNA synthetase 2, mitochondrial	2,33	1,93	FAIL
DDX6	P26196	DEAD-box helicase 6	2,33	2,15	FAIL

MAP3K4	F5H538	mitogen-activated protein kinase kinase kinase 4	2,33	1,74	FAIL
TGS1	Q96RS0	trimethylguanosine synthase 1	2,33	1,61	FAIL
PREP	P48147	prolyl endopeptidase	2,33	3,17	FAIL
STXBP3	O00186	syntaxin binding protein 3	2,33	3,71	FAIL
RMC1	K7ENL9	regulator of MON1-CCZ1	2,33	2,62	FAIL
YWHAZ	P63104	tyrosine 3-monooxygenase/tryptophan 5-monooxygen-ase activation protein zeta	2,32	3,99	FAIL
PLK1	P53350	polo like kinase 1	2,32	2,90	FAIL
KIAA1217	Q5T5P2	KIAA1217	2,32	3,58	FAIL
AKT1	P31749	AKT serine/threonine kinase 1	2,32	2,27	FAIL
COMMD6	Q7Z4G1	COMM domain containing 6	2,32	1,39	FAIL
EEF1G	P26641	eukaryotic translation elongation factor 1 gamma	2,32	4,65	FAIL
VAC14	Q08AM6	VAC14 component of PIKFYVE complex	2,32	1,97	FAIL
NARS1	O43776	asparaginyl-tRNA synthetase 1	2,32	2,40	FAIL
UTP20	O75691	UTP20 small subunit processome component	2,32	1,55	FAIL
VPS18	Q9P253	VPS18 core subunit of CORVET and HOPS complexes	2,32	2,02	FAIL
TUBGCP5	Q96RT8	tubulin gamma complex component 5	2,32	3,13	FAIL
AKAP9	A0A0A0MRF6	A-kinase anchoring protein 9	2,32	1,75	FAIL
VAMP7	P51809	vesicle associated membrane protein 7	2,31	1,89	FAIL
MDM4	A0A087WZ58	MDM4 regulator of p53	2,31	2,16	FAIL
NDUFB1	O75438	NADH:ubiquinone oxidoreductase subunit B1	2,31	2,00	FAIL
KIAA0930	Q6ICG6	KIAA0930	2,31	2,73	FAIL
FASTKD1	Q53R41	FAST kinase domains 1	2,31	1,70	FAIL
KDM2B	Q8NHM5	lysine demethylase 2B	2,31	1,80	FAIL
PABPC4	Q13310	poly(A) binding protein cytoplasmic 4	2,31	1,90	FAIL
BICD2	Q8TD16	BICD cargo adaptor 2	2,31	2,93	FAIL
PHKB	Q93100	phosphorylase kinase regulatory subunit beta	2,31	3,49	FAIL
ZFYVE1	G3V5N8	zinc finger FYVE-type containing 1	2,31	1,48	FAIL
RANBP1	P43487	RAN binding protein 1	2,30	1,85	FAIL
NOL4L	A0A087X0N3	nucleolar protein 4 like	2,30	1,72	FAIL
RHOT2	Q8IXI1	ras homolog family member T2	2,30	1,45	FAIL
TJP2	Q9UDY2	tight junction protein 2	2,30	4,13	FAIL
CGN	Q9P2M7	cingulin	2,30	1,55	FAIL
TRAPPC3	O43617	trafficking protein particle complex subunit 3	2,30	1,50	FAIL
MSL2	Q9HCI7	MSL complex subunit 2	2,30	3,26	FAIL
GTPBP1	O00178	GTP binding protein 1	2,30	3,34	FAIL
AGAP3	Q96P47	ArfGAP with GTPase domain, ankyrin repeat and PH domain 3	2,30	1,37	FAIL
AP1S1	A0A2R8YGH5	adaptor related protein complex 1 subunit sigma 1	2,30	2,41	FAIL
AFTPH	Q6ULP2	aftiphilin	2,30	1,56	FAIL
ANKFY1	Q9P2R3	ankyrin repeat and FYVE domain containing 1	2,30	1,83	FAIL
PSMD7	P51665	proteasome 26S subunit, non-ATPase 7	2,29	2,29	FAIL
GPR75-ASB3	Q9Y575	GPR75-ASB3 readthrough	2,29	1,36	FAIL
VAPB	O95292	VAMP associated protein B and C	2,29	2,32	FAIL
NRAS	P01111	NRAS proto-oncogene, GTPase	2,29	1,82	FAIL
TUBGCP6	E7EQL8	tubulin gamma complex component 6	2,29	4,15	FAIL

PDE4DIP	E9PQG4	phosphodiesterase 4D interacting protein	2,29	2,31	FAIL
ZFP36L2	P47974	ZFP36 ring finger protein like 2	2,29	2,10	FAIL
THADA	Q6YHU6	THADA armadillo repeat containing	2,29	1,56	FAIL
ADA	P00813	adenosine deaminase	2,29	4,42	FAIL
G6PD	P11413	glucose-6-phosphate dehydrogenase	2,28	4,93	FAIL
EIF3I	Q13347	eukaryotic translation initiation factor 3 subunit I	2,28	3,00	FAIL
CAND1	Q86VP6	cullin associated and neddylation dissociated 1	2,28	2,75	FAIL
PPP1R7	C9JD73	protein phosphatase 1 regulatory subunit 7	2,28	2,07	FAIL
LZTS1	Q9Y250	leucine zipper tumor suppressor 1	2,28	2,55	FAIL
MED11	Q9P086	mediator complex subunit 11	2,28	3,14	FAIL
LANCL2	Q9NS86	LanC like glutathione S-transferase 2	2,28	2,30	FAIL
ZNHIT6	Q9NWK9	zinc finger HIT-type containing 6	2,28	2,61	FAIL
METTL15	A6NJ78	methyltransferase 15, mitochondrial 12S rRNA N4-cytidine	2,28	1,59	FAIL
GALK1	P51570	galactokinase 1	2,27	1,74	FAIL
	A0A0G2JMG9		2,27	2,26	FAIL
SLBP	F8W8D3	stem-loop histone mRNA binding protein	2,27	1,46	FAIL
BLTP3A	Q6BDS2	bridge-like lipid transfer protein family member 3A	2,27	2,08	FAIL
TRIM32	Q13049	tripartite motif containing 32	2,27	1,55	FAIL
NDUFV1	G3V0I5	NADH:ubiquinone oxidoreductase core subunit V1	2,27	1,72	FAIL
PDCD10	C9JND6	programmed cell death 10	2,27	1,83	FAIL
CCNK	O75909	cyclin K	2,27	2,46	FAIL
WDR59	Q6PJI9	WD repeat domain 59	2,27	2,02	FAIL
HNRNPD	Q14103	heterogeneous nuclear ribonucleoprotein D	2,27	1,90	FAIL
ALG6	S4R350	ALG6 alpha-1,3-glucosyltransferase	2,27	2,24	FAIL
PDE3B	Q13370	phosphodiesterase 3B	2,27	1,83	FAIL
TERF2IP	Q9NYB0	TERF2 interacting protein	2,26	1,66	FAIL
ILKAP	Q9H0C8	ILK associated serine/threonine phosphatase	2,26	2,02	FAIL
PGD	P52209	phosphogluconate dehydrogenase	2,26	3,62	FAIL
BECN1	Q14457	beclin 1	2,26	2,13	FAIL
RPP25L	Q8N5L8	ribonuclease P/MRP subunit p25 like	2,26	3,50	FAIL
	J3QTR3		2,26	1,57	FAIL
ITGA4	P13612	integrin subunit alpha 4	2,25	2,53	FAIL
METTL2B	Q6P1Q9	methyltransferase 2B, tRNA N3-cytidine	2,25	2,93	FAIL
RCN2	Q14257	reticulocalbin 2	2,25	1,63	FAIL
PHKG2	J3KNN3	phosphorylase kinase catalytic subunit gamma 2	2,25	2,12	FAIL
EDRF1	Q3B7T1	erythroid differentiation regulatory factor 1	2,25	1,36	FAIL
SEPTIN9	Q9UHD8	septin 9	2,25	2,61	FAIL
KIF13B	Q9NQT8	kinesin family member 13B	2,25	2,27	FAIL
URGCP	Q8TCY9	upregulator of cell proliferation	2,25	1,93	FAIL
CYFIP2	E7EVJ5	cytoplasmic FMR1 interacting protein 2	2,24	3,44	FAIL
ASF1A	Q9Y294	anti-silencing function 1A histone chaperone	2,24	2,56	FAIL
HAUS8	Q9BT25	HAUS augmin like complex subunit 8	2,24	3,05	FAIL
MARCHF5	Q9NX47	membrane associated ring-CH-type finger 5	2,24	2,24	FAIL
	A0A2R8Y611		2,24	1,73	FAIL
STYXL2	Q5VZP5	serine/threonine/tyrosine interacting like 2	2,24	2,45	FAIL
MAPKAPK3	Q16644	MAPK activated protein kinase 3	2,24	2,44	FAIL
BIN2	Q9UBW5	bridging integrator 2	2,24	2,67	FAIL

PAK4	O96013	p21 (RAC1) activated kinase 4	2,24	2,22	FAIL
TMEM209	Q96SK2	transmembrane protein 209	2,24	2,21	FAIL
NELFE	P18615	negative elongation factor complex member E	2,24	1,78	FAIL
LLGL1	Q15334	LLGL scribble cell polarity complex component 1	2,24	1,88	FAIL
POLR1D	P0DPB6	RNA polymerase I and III subunit D	2,24	2,76	FAIL
SLC25A46	Q96AG3	solute carrier family 25 member 46	2,23	1,89	FAIL
KIF4A	O95239	kinesin family member 4A	2,23	3,30	FAIL
LSM14B	Q9BX40	LSM family member 14B	2,23	1,98	FAIL
CDC16	Q13042	cell division cycle 16	2,23	2,01	FAIL
PRAF2	O60831	PRA1 domain family member 2	2,23	2,02	FAIL
NRAP	A0A0A0MRM2	nebulin related anchoring protein	2,23	4,35	FAIL
STX16	O14662	syntaxin 16	2,23	2,00	FAIL
PTPRC	P08575	protein tyrosine phosphatase receptor type C	2,23	1,82	FAIL
GNAS	Q5JWF2	GNAS complex locus	2,23	2,19	FAIL
TPR	P12270	translocated promoter region, nuclear basket protein	2,23	2,30	FAIL
PCM1	A0A5H1ZRS1	pericentriolar material 1	2,23	4,88	FAIL
EYA3	Q99504	EYA transcriptional coactivator and phosphatase 3	2,22	3,36	FAIL
MTOR	P42345	mechanistic target of rapamycin kinase	2,22	2,22	FAIL
EXOSC10	Q01780	exosome component 10	2,22	3,19	FAIL
ORC4	O43929	origin recognition complex subunit 4	2,22	1,59	FAIL
FANCD2	Q9BXW9	FA complementation group D2	2,22	1,31	FAIL
POLD2	P49005	DNA polymerase delta 2, accessory subunit	2,22	1,59	FAIL
REXO1	Q8N1G1	RNA exonuclease 1 homolog	2,22	1,51	FAIL
YWHAG	P61981	tyrosine 3-monooxygenase/tryptophan 5-monooxygenase activation protein gamma	2,22	4,43	FAIL
CAPNS1	P04632	calpain small subunit 1	2,22	1,31	FAIL
GATB	D6REA0	glutamyl-tRNA amidotransferase subunit B	2,21	2,31	FAIL
TRAFD1	O14545	TRAF-type zinc finger domain containing 1	2,21	4,09	FAIL
TMPO	P42166	thymopoietin	2,21	4,01	FAIL
MIER1	Q8N108	MIER1 transcriptional regulator	2,21	1,61	FAIL
TMUB1	Q9BVT8	transmembrane and ubiquitin like domain containing 1	2,21	1,41	FAIL
TRIM56	Q9BRZ2	tripartite motif containing 56	2,21	3,19	FAIL
ADAM10	O14672	ADAM metallopeptidase domain 10	2,21	2,63	FAIL
DCTN3	X6RCK5	dynactin subunit 3	2,21	4,31	FAIL
SEPTIN1	Q8WYJ6	septin 1	2,21	4,03	FAIL
AP1G2	G3V2V9	adaptor related protein complex 1 subunit gamma 2	2,21	3,08	FAIL
RASAL3	Q86YV0	RAS protein activator like 3	2,21	2,19	FAIL
TBC1D9B	Q66K14	TBC1 domain family member 9B	2,20	2,14	FAIL
NAP1L1	H0YIV4	nucleosome assembly protein 1 like 1	2,20	3,13	FAIL
EPB41L5	Q9HCM4	erythrocyte membrane protein band 4.1 like 5	2,20	2,58	FAIL
ZMYM4	Q5VZL5	zinc finger MYM-type containing 4	2,20	2,01	FAIL
SYNCRIP	O60506	synaptotagmin binding cytoplasmic RNA interacting protein	2,20	2,08	FAIL
PPP2R2A	P63151	protein phosphatase 2 regulatory subunit Balpha	2,20	2,14	FAIL
SART3	Q15020	spliceosome associated factor 3, U4/U6 recycling protein	2,20	1,31	FAIL
SLC35B2	Q8TB61	solute carrier family 35 member B2	2,20	2,05	FAIL

EIF5	P55010	eukaryotic translation initiation factor 5	2,20	1,89	FAIL
KEAP1	Q14145	kelch like ECH associated protein 1	2,20	2,51	FAIL
VAV2	P52735	vav guanine nucleotide exchange factor 2	2,20	1,40	FAIL
GTF3C5	Q9Y5Q8	general transcription factor IIIC subunit 5	2,20	2,62	FAIL
UHRF1	Q96T88	ubiquitin like with PHD and ring finger domains 1	2,20	2,56	FAIL
STUB1	Q9UNE7	STIP1 homology and U-box containing protein 1	2,19	4,05	FAIL
NSDHL	Q15738	NAD(P) dependent steroid dehydrogenase-like	2,19	1,47	FAIL
STAT2	P52630	signal transducer and activator of transcription 2	2,19	1,44	FAIL
TRAPPC5	Q8IUR0	trafficking protein particle complex subunit 5	2,19	1,95	FAIL
BAG3	O95817	BAG cochaperone 3	2,19	2,39	FAIL
IKBKG	Q9Y6K9	inhibitor of nuclear factor kappa B kinase regulatory subunit gamma	2,19	2,43	FAIL
SEC11A	H0YNG3	SEC11 homolog A, signal peptidase complex subunit	2,19	1,68	FAIL
PLAA	Q9Y263	phospholipase A2 activating protein	2,18	3,15	FAIL
ACAP1	Q15027	ArfGAP with coiled-coil, ankyrin repeat and PH domains 1	2,18	2,11	FAIL
IPO7	O95373	importin 7	2,18	3,57	FAIL
DCAF1	Q9Y4B6	DDB1 and CUL4 associated factor 1	2,18	1,98	FAIL
WDR6	A0A087X295	WD repeat domain 6	2,18	2,67	FAIL
RPL37A	P61513	ribosomal protein L37a	2,18	1,33	FAIL
HK1	P19367	hexokinase 1	2,18	1,32	FAIL
PES1	B5MCF9	pescadillo ribosomal biogenesis factor 1	2,18	4,07	FAIL
VPS51	Q9UID3	VPS51 subunit of GARP complex	2,18	3,94	FAIL
GCDH	Q92947	glutaryl-CoA dehydrogenase	2,18	1,65	FAIL
YBX2	Q9Y2T7	Y-box binding protein 2	2,17	2,25	FAIL
HLA-B	P01889	major histocompatibility complex, class I, B	2,17	2,17	FAIL
ARFGAP2	A0A0D9SF70	ADP ribosylation factor GTPase activating protein 2	2,17	3,85	FAIL
EIF4A1	P60842	eukaryotic translation initiation factor 4A1	2,17	2,23	FAIL
LATS1	O95835	large tumor suppressor kinase 1	2,17	2,41	FAIL
ATPAF2	Q8N5M1	ATP synthase mitochondrial F1 complex assembly factor 2	2,17	1,33	FAIL
GRK6	F8W9W2	G protein-coupled receptor kinase 6	2,17	3,34	FAIL
SETD1A	O15047	SET domain containing 1A, histone lysine methyltransferase	2,17	1,71	FAIL
AKAP13	Q12802	A-kinase anchoring protein 13	2,17	2,87	FAIL
IFT25	A6NIR2	intraflagellar transport 25	2,16	1,50	FAIL
MICAL1	Q8TDZ2	microtubule associated monooxygenase, calponin and LIM domain containing 1	2,16	1,80	FAIL
PGRMC1	O00264	progesterone receptor membrane component 1	2,16	5,11	FAIL
GNAI2	P04899	G protein subunit alpha i2	2,16	4,30	FAIL
ANXA4	P09525	annexin A4	2,16	2,36	FAIL
R3HDM2	B5MCG9	R3H domain containing 2	2,16	1,66	FAIL
PNPLA6	A0A384DVU0	patatin like phospholipase domain containing 6	2,16	2,89	FAIL
PPFIA3	O75145	PTPRF interacting protein alpha 3	2,16	4,20	FAIL
SUMO1	P63165	small ubiquitin like modifier 1	2,16	2,02	FAIL
TMEM87A	Q8NBN3	transmembrane protein 87A	2,16	1,88	FAIL

PARP4	Q9UKK3	poly(ADP-ribose) polymerase family member 4	2,16	1,58	FAIL
EIF4G1	Q04637	eukaryotic translation initiation factor 4 gamma 1	2,16	1,73	FAIL
C1QBP	Q07021	complement C1q binding protein	2,16	1,53	FAIL
KIF7	Q2M1P5	kinesin family member 7	2,16	1,91	FAIL
KMT2A	Q03164	lysine methyltransferase 2A	2,15	1,83	FAIL
COMMD2	Q86X83	COMM domain containing 2	2,15	2,86	FAIL
TUBE1	S4R3K3	tubulin epsilon 1	2,15	1,32	FAIL
MAP2K2	P36507	mitogen-activated protein kinase kinase 2	2,15	1,47	FAIL
	H0Y6I6		2,15	1,80	FAIL
RNF20	Q5VTR2	ring finger protein 20	2,15	2,74	FAIL
SECISBP2L	Q93073	SECIS binding protein 2 like	2,15	1,50	FAIL
FBL	P22087	fibrillarin	2,15	4,93	FAIL
SERPINH1	P50454	serpin family H member 1	2,15	1,95	FAIL
UQCC1	Q9NVA1	ubiquinol-cytochrome c reductase complex assembly factor 1	2,15	2,12	FAIL
ATXN10	Q9UBB4	ataxin 10	2,15	2,20	FAIL
GABARAPL2	H3BQ50	GABA type A receptor associated protein like 2	2,15	2,29	FAIL
DNAJA1	P31689	DnaJ heat shock protein family (Hsp40) member A1	2,15	2,65	FAIL
SLC25A19	Q9HC21	solute carrier family 25 member 19	2,14	1,62	FAIL
PRIM1	A0A5F9ZHB6	DNA primase subunit 1	2,14	2,93	FAIL
FGD6	Q6ZV73	FYVE, RhoGEF and PH domain containing 6	2,14	1,56	FAIL
HYOU1	Q9Y4L1	hypoxia up-regulated 1	2,14	1,70	FAIL
CRTAP	O75718	cartilage associated protein	2,14	1,76	FAIL
QRICH1	Q2TAL8	glutamine rich 1	2,14	2,00	FAIL
GPS1	Q13098	G protein pathway suppressor 1	2,14	1,83	FAIL
OSGEP	Q9NPF4	O-sialoglycoprotein endopeptidase	2,14	3,46	FAIL
NCAPG	Q9BPX3	non-SMC condensin I complex subunit G	2,13	2,05	FAIL
RAB8B	Q92930	RAB8B, member RAS oncogene family	2,13	2,10	FAIL
FASN	P49327	fatty acid synthase	2,13	2,51	FAIL
HNRNPAB	Q99729	heterogeneous nuclear ribonucleoprotein A/B	2,13	2,31	FAIL
RBM28	Q9NW13	RNA binding motif protein 28	2,13	2,78	FAIL
FLII	J3KS54	FLII actin remodeling protein	2,13	4,90	FAIL
TOMM40	O96008	translocase of outer mitochondrial membrane 40	2,13	1,34	FAIL
HDLBP	Q00341	high density lipoprotein binding protein	2,13	3,72	FAIL
TAMM41	Q96BW9	TAM41 mitochondrial translocator assembly and maintenance homolog	2,13	1,56	FAIL
WEE1	E9PQ51	WEE1 G2 checkpoint kinase	2,13	1,60	FAIL
RAP1GDS1	P52306	Rap1 GTPase-GDP dissociation stimulator 1	2,13	1,36	FAIL
LRR1	Q96L50	leucine rich repeat protein 1	2,13	3,29	FAIL
PRKD2	Q9BZL6	protein kinase D2	2,13	1,61	FAIL
CD3D	P04234	CD3 delta subunit of T-cell receptor complex	2,13	3,15	FAIL
	A0A1B0GX12		2,13	3,80	FAIL
MCUR1	Q96AQ8	mitochondrial calcium uniporter regulator 1	2,12	1,77	FAIL
ALG2	Q9H553	ALG2 alpha-1,3/1,6-mannosyltransferase	2,12	1,45	FAIL
KPNB1	Q14974	karyopherin subunit beta 1	2,12	2,68	FAIL
TRIM14	Q14142	tripartite motif containing 14	2,12	3,02	FAIL

VPS36	Q86VN1	vacuolar protein sorting 36 homolog	2,12	2,25	FAIL
DNTTIP2	Q5QJE6	deoxynucleotidyltransferase terminal interacting protein 2	2,12	3,40	FAIL
RPL27	P61353	ribosomal protein L27	2,12	4,38	FAIL
CDC25C	P30307	cell division cycle 25C	2,12	2,52	FAIL
DTX3L	Q8TDB6	deltex E3 ubiquitin ligase 3L	2,12	1,33	FAIL
UBLCP1	Q8WVY7	ubiquitin like domain containing CTD phosphatase 1	2,12	1,37	FAIL
COASY	Q13057	Coenzyme A synthase	2,12	4,91	FAIL
	A0A0A0MTP3		2,12	2,52	FAIL
PPP4R1	Q8TF05	protein phosphatase 4 regulatory subunit 1	2,12	3,52	FAIL
AASDHPPT	Q9NRN7	aminoadipate-semialdehyde dehydrogenase-phosphopantetheinyl transferase	2,12	1,46	FAIL
CAMK2G	A0A2Q3DQE3	calcium/calmodulin dependent protein kinase II gamma	2,12	4,24	FAIL
PITPNM2	Q9BZ72	phosphatidylinositol transfer protein membrane associated 2	2,11	3,77	FAIL
NAGK	Q9UJ70	N-acetylglucosamine kinase	2,11	2,52	FAIL
COG5	Q9UP83	component of oligomeric golgi complex 5	2,11	2,87	FAIL
NUP188	Q5SRE5	nucleoporin 188	2,11	1,87	FAIL
MKRN2	Q9H000	makorin ring finger protein 2	2,11	2,59	FAIL
GEMIN4	I3L2C7	gem nuclear organelle associated protein 4	2,11	2,57	FAIL
PSMD11	O00231	proteasome 26S subunit, non-ATPase 11	2,11	2,04	FAIL
ARMH3	Q5T2E6	armadillo like helical domain containing 3	2,11	2,12	FAIL
CSDE1	O75534	cold shock domain containing E1	2,11	3,17	FAIL
GET1	B7Z1T1	guided entry of tail-anchored proteins factor 1	2,11	2,61	FAIL
ZNF384	Q8TF68	zinc finger protein 384	2,10	3,97	FAIL
PPIL2	Q13356	peptidylprolyl isomerase like 2	2,10	3,09	FAIL
ZC3H7A	Q8IWR0	zinc finger CCCH-type containing 7A	2,10	1,57	FAIL
TTLL12	Q14166	tubulin tyrosine ligase like 12	2,10	2,81	FAIL
YWHAH	Q04917	tyrosine 3-monooxygenase/tryptophan 5-monooxygenase activation protein eta	2,10	4,06	FAIL
B2M	H0YLF3	beta-2-microglobulin	2,10	3,04	FAIL
PEX5L	Q8IYB4	peroxisomal biogenesis factor 5 like	2,10	2,09	FAIL
KLF16	Q9BXK1	KLF transcription factor 16	2,09	1,40	FAIL
HCFC1	A6NEM2	host cell factor C1	2,09	2,43	FAIL
UBE3C	Q15386	ubiquitin protein ligase E3C	2,09	3,00	FAIL
TMEM126A	Q9H061	transmembrane protein 126A	2,09	2,48	FAIL
NSD2	O96028	nuclear receptor binding SET domain protein 2	2,09	1,80	FAIL
NBN	O60934	nibrin	2,09	2,10	FAIL
TADA3	O75528	transcriptional adaptor 3	2,09	1,32	FAIL
FNDC3A	Q9Y2H6	fibronectin type III domain containing 3A	2,09	1,45	FAIL
EMC8	M0R1B0	ER membrane protein complex subunit 8	2,09	1,71	FAIL
UBE2L6	O14933	ubiquitin conjugating enzyme E2 L6	2,09	3,07	FAIL
FAM234A	B4DGG1	family with sequence similarity 234 member A	2,09	1,57	FAIL
GPAA1	E9PM11	glycosylphosphatidylinositol anchor attachment 1	2,09	1,83	FAIL
PSMC4	P43686	proteasome 26S subunit, ATPase 4	2,09	1,54	FAIL
CCDC191	Q8NCU4	coiled-coil domain containing 191	2,08	1,77	FAIL

NEO1	Q92859	neogenin 1	2,08	1,91	FAIL
WASF2	Q9Y6W5	WASP family member 2	2,08	2,60	FAIL
SMARCE1	A0A2R8Y855	SWI/SNF related, matrix associated, actin dependent regulator of chromatin, subfamily e, member 1	2,08	2,18	FAIL
ELMO1	Q92556	engulfment and cell motility 1	2,08	3,15	FAIL
KTN1	Q86UP2	kinectin 1	2,08	2,85	FAIL
ERCC6L	Q2NKX8	ERCC excision repair 6 like, spindle assembly checkpoint helicase	2,08	3,17	FAIL
TYSND1	Q2T9J0	trypsin like peroxisomal matrix peptidase 1	2,08	2,80	FAIL
SLC25A11	Q02978	solute carrier family 25 member 11	2,08	1,33	FAIL
THOC6	Q86W42	THO complex subunit 6	2,08	1,79	FAIL
RAB2B	Q8WUD1	RAB2B, member RAS oncogene family	2,08	3,30	FAIL
RAD21	O60216	RAD21 cohesin complex component	2,08	2,42	FAIL
CAD	P27708	carbamoyl-phosphate synthetase 2, aspartate transcarbamylase, and dihydroorotase	2,08	3,09	FAIL
CSNK2A2	P19784	casein kinase 2 alpha 2	2,08	2,70	FAIL
MPG	P29372	N-methylpurine DNA glycosylase	2,08	1,91	FAIL
AKNA	Q7Z591	AT-hook transcription factor	2,08	1,32	FAIL
ATXN2	F8WB06	ataxin 2	2,08	3,07	FAIL
UBE4A	Q14139	ubiquitination factor E4A	2,08	1,43	FAIL
MED22	E7EN96	mediator complex subunit 22	2,07	4,13	FAIL
UTP11	Q9Y3A2	UTP11 small subunit processome component	2,07	1,48	FAIL
PIK3R2	O00459	phosphoinositide-3-kinase regulatory subunit 2	2,07	1,82	FAIL
BRF1	Q92994	BRF1 RNA polymerase III transcription initiation factor subunit	2,07	1,95	FAIL
CSNK2B	P67870	casein kinase 2 beta	2,07	2,14	FAIL
NPAT	Q14207	nuclear protein, coactivator of histone transcription	2,07	1,45	FAIL
ZNF512B	Q96KM6	zinc finger protein 512B	2,07	2,11	FAIL
EIF3K	Q9UBQ5	eukaryotic translation initiation factor 3 subunit K	2,07	2,11	FAIL
CLINT1	Q14677	clathrin interactor 1	2,07	2,29	FAIL
RBBP7	E9PC52	RB binding protein 7, chromatin remodeling factor	2,07	2,43	FAIL
MCRS1	Q96EZ8	microspherule protein 1	2,07	2,65	FAIL
KNOP1	Q1ED39	lysine rich nucleolar protein 1	2,07	2,29	FAIL
TRNT1	Q96Q11	tRNA nucleotidyl transferase 1	2,07	2,91	FAIL
C8orf33	A0A3B3IT54	chromosome 8 open reading frame 33	2,06	2,26	FAIL
RWDD2A	Q9UIY3	RWD domain containing 2A	2,06	1,74	FAIL
FBXO11	Q86XK2	F-box protein 11	2,06	1,83	FAIL
CDIN1	H3BNF9	CDAN1 interacting nuclease 1	2,06	1,59	FAIL
USP24	Q9UPU5	ubiquitin specific peptidase 24	2,06	3,65	FAIL
RP2	O75695	RP2 activator of ARL3 GTPase	2,06	4,16	FAIL
CAAP1	Q9H8G2	caspase activity and apoptosis inhibitor 1	2,06	1,55	FAIL
LCK	P06239	LCK proto-oncogene, Src family tyrosine kinase	2,06	2,23	FAIL
GATD1	H0YE25	glutamine amidotransferase class 1 domain containing 1	2,06	1,49	FAIL
SLC39A11	J3KRX2	solute carrier family 39 member 11	2,06	2,81	FAIL

PIK3R4	Q99570	phosphoinositide-3-kinase regulatory subunit 4	2,06	3,37	FAIL
GTF3C3	Q9Y5Q9	general transcription factor IIIC subunit 3	2,06	2,17	FAIL
TBC1D10A	Q9BXI6	TBC1 domain family member 10A	2,06	1,63	FAIL
ARFIP1	P53367	ADP ribosylation factor interacting protein 1	2,06	1,41	FAIL
SLC16A7	O60669	solute carrier family 16 member 7	2,06	1,39	FAIL
RHOA	P61586	ras homolog family member A	2,06	2,16	FAIL
DPF2	J3KMZ8	double PHD fingers 2	2,05	1,54	FAIL
SNF8	Q96H20	SNF8 subunit of ESCRT-II	2,05	1,49	FAIL
AGK	E9PC15	acylglycerol kinase	2,05	1,56	FAIL
TROAP	Q12815	trophinin associated protein	2,05	2,32	FAIL
PUM1	Q14671	pumilio RNA binding family member 1	2,05	2,35	FAIL
ARIH1	Q9Y4X5	ariadne RBR E3 ubiquitin protein ligase 1	2,05	2,80	FAIL
WAC	Q9BTA9	WW domain containing adaptor with coiled-coil	2,05	2,01	FAIL
NAA15	Q9BXJ9	N-alpha-acetyltransferase 15, NatA auxiliary subunit	2,05	2,86	FAIL
PCNT	O95613	pericentrin	2,05	1,62	FAIL
GLS	O94925	glutaminase	2,05	1,75	FAIL
GPR180	Q86V85	G protein-coupled receptor 180	2,05	2,48	FAIL
BRAT1	Q6PJG6	BRCA1 associated ATM activator 1	2,04	1,74	FAIL
PARP10	E9PK67	poly(ADP-ribose) polymerase family member 10	2,04	1,91	FAIL
ZNG1B	Q8IUF1	Zn regulated GTPase metalloprotein activator 1B	2,04	1,87	FAIL
PCGF6	Q9BYE7	polycomb group ring finger 6	2,04	1,36	FAIL
EFTUD2	Q15029	elongation factor Tu GTP binding domain containing 2	2,04	1,65	FAIL
SPNS1	H3BPQ9	SPNS lysolipid transporter 1, lysophospholipid	2,04	2,19	FAIL
KTI12	Q96EK9	KTI12 chromatin associated homolog	2,04	1,50	FAIL
NRM	Q8IXM6	nurim	2,04	1,54	FAIL
STIM1	Q13586	stromal interaction molecule 1	2,04	3,16	FAIL
CEP72	Q9P209	centrosomal protein 72	2,04	2,50	FAIL
MOGS	Q13724	mannosyl-oligosaccharide glucosidase	2,04	2,91	FAIL
RPL9	P32969	ribosomal protein L9	2,03	1,42	FAIL
TRIP12	Q14669	thyroid hormone receptor interactor 12	2,03	2,42	FAIL
SKIC8	Q9GZS3	SKI8 subunit of superkiller complex	2,03	1,47	FAIL
WTAP	Q15007	WT1 associated protein	2,03	1,69	FAIL
SCCPDH	Q8NBX0	saccharopine dehydrogenase (putative)	2,03	3,39	FAIL
OGFR	Q9NZT2	opioid growth factor receptor	2,03	2,31	FAIL
TRAF6	Q9Y4K3	TNF receptor associated factor 6	2,03	2,67	FAIL
SUPT5H	O00267	SPT5 homolog, DSIF elongation factor subunit	2,03	4,09	FAIL
NUP50	Q9UKX7	nucleoporin 50	2,03	3,48	FAIL
OSBP	P22059	oxysterol binding protein	2,03	2,03	FAIL
USP14	P54578	ubiquitin specific peptidase 14	2,03	1,81	FAIL
LRRC41	Q15345	leucine rich repeat containing 41	2,03	2,20	FAIL
OTULIN	Q96BN8	OTU deubiquitinase with linear linkage specificity	2,03	4,12	FAIL
CFL1	E9PK25	cofilin 1	2,03	4,82	FAIL
DCUN1D5	Q9BTE7	defective in cullin neddylation 1 domain containing 5	2,03	1,64	FAIL
PPP2R5A	Q15172	protein phosphatase 2 regulatory subunit B'alpha	2,03	1,88	FAIL
TOP3A	Q13472	DNA topoisomerase III alpha	2,03	1,46	FAIL
FARSB	Q9NSD9	phenylalanyl-tRNA synthetase subunit beta	2,03	4,66	FAIL
PRMT5	O14744	protein arginine methyltransferase 5	2,03	2,58	FAIL
IGF2R	P11717	insulin like growth factor 2 receptor	2,02	2,51	FAIL

	A0A024RCR6		2,02	1,64	FAIL
PPA2	Q9H2U2	inorganic pyrophosphatase 2	2,02	2,74	FAIL
PEX16	E9PLS4	peroxisomal biogenesis factor 16	2,02	2,04	FAIL
RECQL4	O94761	RecQ like helicase 4	2,02	2,17	FAIL
RPS6	P62753	ribosomal protein S6	2,02	2,29	FAIL
ASH2L	Q9UBL3	ASH2 like, histone lysine methyltransferase complex subunit	2,02	3,13	FAIL
NACC1	Q96RE7	nucleus accumbens associated 1	2,02	1,72	FAIL
TRMT6	Q9UJA5	tRNA methyltransferase 6 non-catalytic subunit	2,02	2,66	FAIL
NDUFB11	Q9NX14	NADH:ubiquinone oxidoreductase subunit B11	2,02	2,72	FAIL
IPO5	O00410	importin 5	2,02	2,81	FAIL
NOP9	Q86U38	NOP9 nucleolar protein	2,02	1,76	FAIL
MARCKSL1	P49006	MARCKS like 1	2,02	1,87	FAIL
SEC13	P55735	SEC13 homolog, nuclear pore and COPII coat complex component	2,02	1,52	FAIL
NDUFAF1	Q9Y375	NADH:ubiquinone oxidoreductase complex assembly factor 1	2,02	2,22	FAIL
GCC2	Q8IWJ2	GRIP and coiled-coil domain containing 2	2,01	3,33	FAIL
RPL14	P50914	ribosomal protein L14	2,01	1,48	FAIL
EDC4	Q6P2E9	enhancer of mRNA decapping 4	2,01	3,79	FAIL
RPL21	P46778	ribosomal protein L21	2,01	2,84	FAIL
RREB1	Q92766	ras responsive element binding protein 1	2,01	1,55	FAIL
	A0A087WU02		2,01	4,67	FAIL
EIF2B1	Q14232	eukaryotic translation initiation factor 2B subunit alpha	2,01	2,49	FAIL
SF3A3	Q12874	splicing factor 3a subunit 3	2,01	1,49	FAIL
OSBPL9	Q96SU4	oxysterol binding protein like 9	2,01	1,89	FAIL
PARL	A0A1W2PS40	presenilin associated rhomboid like	2,01	2,09	FAIL
PRRC2A	P48634	proline rich coiled-coil 2A	2,01	2,24	FAIL
TUBGCP2	Q9BSJ2	tubulin gamma complex component 2	2,00	1,91	FAIL
SLC25A12	O75746	solute carrier family 25 member 12	2,00	3,60	FAIL
IRF2BP2	Q7Z5L9	interferon regulatory factor 2 binding protein 2	2,00	2,69	FAIL
CTNNA1	P35221	catenin alpha 1	2,00	4,05	FAIL
FBXO34	Q9NWN3	F-box protein 34	2,00	3,01	FAIL
RDH13	Q8NBN7	retinol dehydrogenase 13	2,00	1,47	FAIL
BRD1	O95696	bromodomain containing 1	2,00	1,33	FAIL
RASA3	Q14644	RAS p21 protein activator 3	2,00	2,17	FAIL
	A0A087WY00		2,00	2,17	FAIL
CEP78	A0A2R8Y589	centrosomal protein 78	2,00	3,12	FAIL
AP1G1	O43747	adaptor related protein complex 1 subunit gamma 1	2,00	2,18	FAIL
NIPSNAP1	Q9BPW8	nipsnap homolog 1	2,00	2,89	FAIL
AP3M1	Q9Y2T2	adaptor related protein complex 3 subunit mu 1	1,99	2,89	FAIL
SNX8	Q9Y5X2	sorting nexin 8	1,99	1,54	FAIL
LRRC40	Q9H9A6	leucine rich repeat containing 40	1,99	3,10	FAIL
AAAS	Q9NRG9	aladin WD repeat nucleoporin	1,99	2,36	FAIL
EXOSC8	Q96B26	exosome component 8	1,99	1,50	FAIL
HSPA4L	O95757	heat shock protein family A (Hsp70) member 4 like	1,99	2,01	FAIL

SENP5	Q96HI0	SUMO specific peptidase 5	1,99	2,62	FAIL
PIAS1	O75925	protein inhibitor of activated STAT 1	1,99	3,00	FAIL
SUGP2	Q8IX01	SURP and G-patch domain containing 2	1,99	2,59	FAIL
CBX4	O00257	chromobox 4	1,98	2,62	FAIL
HINT3	Q9NQE9	histidine triad nucleotide binding protein 3	1,98	4,92	FAIL
GTF2I	P78347	general transcription factor IIi	1,98	2,25	FAIL
LONP1	K7EKE6	lon peptidase 1, mitochondrial	1,98	1,59	FAIL
SUPT6H	Q7KZ85	SPT6 homolog, histone chaperone and transcription elongation factor	1,98	2,38	FAIL
BPHL	Q86W56	biphenyl hydrolase like	1,98	1,94	FAIL
NOC2L	Q9Y3T9	NOC2 like nucleolar associated transcriptional re-pressor	1,98	3,83	FAIL
MRPS7	J3QKW2	mitochondrial ribosomal protein S7	1,98	2,39	FAIL
RPS6KA1	Q15418	ribosomal protein S6 kinase A1	1,98	2,33	FAIL
ATAD2	Q6PL18	ATPase family AAA domain containing 2	1,98	1,79	FAIL
CHEK1	O14757	checkpoint kinase 1	1,98	3,05	FAIL
ERAP2	Q6P179	endoplasmic reticulum aminopeptidase 2	1,98	1,70	FAIL
RNF25	Q96BH1	ring finger protein 25	1,98	3,14	FAIL
METTL16	Q86W50	methyltransferase 16, RNA N6-adenosine	1,98	1,48	FAIL
RBM4	Q9BWF3	RNA binding motif protein 4	1,98	2,56	FAIL
TIMM23	O14925	translocase of inner mitochondrial membrane 23	1,98	2,12	FAIL
MTX2	C9JAZ1	metaxin 2	1,98	2,29	FAIL
UBL4A	P11441	ubiquitin like 4A	1,98	1,71	FAIL
EIF5A	I3L397	eukaryotic translation initiation factor 5A	1,98	2,98	FAIL
PLCB1	A0A087WT80	phospholipase C beta 1	1,98	2,32	FAIL
ATP1A1	P05023	ATPase Na+/K+ transporting subunit alpha 1	1,97	3,34	FAIL
PRPSAP2	O60256	phosphoribosyl pyrophosphate synthetase associated protein 2	1,97	2,03	FAIL
SLC1A5	Q15758	solute carrier family 1 member 5	1,97	2,47	FAIL
DRG1	Q9Y295	developmentally regulated GTP binding protein 1	1,97	2,73	FAIL
GALE	Q14376	UDP-galactose-4-epimerase	1,97	1,60	FAIL
SIPA1	F6RY50	signal-induced proliferation-associated 1	1,97	2,62	FAIL
PCMT1	P22061	protein-L-isoaspartate (D-aspartate) O-methyltransfer-ase	1,97	4,11	FAIL
ARHGDIB	P52566	Rho GDP dissociation inhibitor beta	1,97	2,15	FAIL
ZC3HAV1	C9J6P4	zinc finger CCCH-type containing, antiviral 1	1,97	2,18	FAIL
PRMT3	O60678	protein arginine methyltransferase 3	1,97	3,51	FAIL
SUCLA2	A0A2R8Y6Y7	succinate-CoA ligase ADP-forming subunit beta	1,96	2,33	FAIL
TBCD	J3KR97	tubulin folding cofactor D	1,96	2,37	FAIL
MAP2K4	P45985	mitogen-activated protein kinase kinase 4	1,96	1,94	FAIL
RANGAP1	P46060	Ran GTPase activating protein 1	1,96	3,28	FAIL
RNASEH2A	O75792	ribonuclease H2 subunit A	1,96	2,06	FAIL
FAR1	Q8WVX9	fatty acyl-CoA reductase 1	1,96	2,36	FAIL
SSR3	C9JA28	signal sequence receptor subunit 3	1,96	2,10	FAIL
LSM12	K7ELG9	LSM12 homolog	1,96	2,36	FAIL

GART	P22102	phosphoribosylglycinamide formyltransferase, phosphoribosylglycinamide synthetase, phosphoribosylaminoimidazole synthetase	1,96	3,39	FAIL
ARFGEF2	Q9Y6D5	ADP ribosylation factor guanine nucleotide exchange factor 2	1,95	1,48	FAIL
SMC3	Q9UQE7	structural maintenance of chromosomes 3	1,95	2,23	FAIL
CHEK2	O96017	checkpoint kinase 2	1,95	1,86	FAIL
NUSAP1	Q9BXS6	nucleolar and spindle associated protein 1	1,95	2,74	FAIL
FMR1	Q06787	fragile X messenger ribonucleoprotein 1	1,95	2,67	FAIL
NCALD	P61601	neurocalcin delta	1,95	1,88	FAIL
RFWD3	Q6PCD5	ring finger and WD repeat domain 3	1,95	2,54	FAIL
IGF2BP1	Q9NZI8	insulin like growth factor 2 mRNA binding protein 1	1,95	2,50	FAIL
IKZF2	Q9UKS7	IKAROS family zinc finger 2	1,95	1,65	FAIL
MRPS10	P82664	mitochondrial ribosomal protein S10	1,95	1,46	FAIL
RAB5B	P61020	RAB5B, member RAS oncogene family	1,95	1,31	FAIL
ZC3H15	Q8WU90	zinc finger CCCH-type containing 15	1,95	4,55	FAIL
SMARCC1	Q92922	SWI/SNF related, matrix associated, actin dependent regulator of chromatin subfamily c member 1	1,95	1,66	FAIL
ESYT2	H7BXI1	extended synaptotagmin 2	1,95	3,17	FAIL
VDAC3	Q9Y277	voltage dependent anion channel 3	1,95	1,73	FAIL
EXOSC9	H0Y9L5	exosome component 9	1,94	4,31	FAIL
AP2S1	M0R0N4	adaptor related protein complex 2 subunit sigma 1	1,94	1,85	FAIL
CPSF3	Q9UKF6	cleavage and polyadenylation specific factor 3	1,94	2,27	FAIL
C17orf75	Q9HAS0	chromosome 17 open reading frame 75	1,94	2,43	FAIL
TTI1	O43156	TELO2 interacting protein 1	1,94	1,81	FAIL
	Q5T3I0		1,94	4,13	FAIL
HNRNPK	P61978	heterogeneous nuclear ribonucleoprotein K	1,94	1,60	FAIL
ATP2C1	P98194	ATPase secretory pathway Ca2+ transporting 1	1,94	1,76	FAIL
FDFT1	P37268	farnesyl-diphosphate farnesyltransferase 1	1,94	2,08	FAIL
NPY1R	P25929	neuropeptide Y receptor Y1	1,94	1,91	FAIL
GEMIN5	Q8TEQ6	gem nuclear organelle associated protein 5	1,93	2,20	FAIL
VPS13A	Q96RL7	vacuolar protein sorting 13 homolog A	1,93	1,98	FAIL
BRIX1	Q8TDN6	biogenesis of ribosomes BRX1	1,93	4,69	FAIL
ALG11	Q2TAA5	ALG11 alpha-1,2-mannosyltransferase	1,93	1,83	FAIL
ATXN2L	Q8WWM7	ataxin 2 like	1,93	1,98	FAIL
EED	A0A5F9ZI63	embryonic ectoderm development	1,93	3,11	FAIL
FAM98A	Q8NCA5	family with sequence similarity 98 member A	1,93	1,42	FAIL
EDEM2	Q9BV94	ER degradation enhancing alpha-mannosidase like protein 2	1,93	1,55	FAIL
BCAT1	P54687	branched chain amino acid transaminase 1	1,93	3,06	FAIL
CLASP2	E7EW49	cytoplasmic linker associated protein 2	1,93	2,87	FAIL
SEC16A	F1T0I1	SEC16 homolog A, endoplasmic reticulum export factor	1,93	3,15	FAIL
ZBTB21	Q9ULJ3	zinc finger and BTB domain containing 21	1,93	1,90	FAIL
LETM1	O95202	leucine zipper and EF-hand containing transmembrane protein 1	1,93	1,42	FAIL

TIMM17B	V9GYS0	translocase of inner mitochondrial membrane 17B	1,92	1,86	FAIL
MRGBP	Q9NV56	MRG domain binding protein	1,92	1,60	FAIL
UFD1	C9J6N9	ubiquitin recognition factor in ER associated degradation 1	1,92	2,61	FAIL
CEP68	Q76N32	centrosomal protein 68	1,92	2,61	FAIL
USP10	Q14694	ubiquitin specific peptidase 10	1,92	2,73	FAIL
GMIP	Q9P107	GEM interacting protein	1,92	2,88	FAIL
MAGT1	Q9H0U3	magnesium transporter 1	1,92	2,99	FAIL
TAP2	X5CMH5	transporter 2, ATP binding cassette subfamily B member	1,92	2,68	FAIL
C11orf58	O00193	chromosome 11 open reading frame 58	1,92	1,77	FAIL
ERP44	Q9BS26	endoplasmic reticulum protein 44	1,92	3,11	FAIL
UBAP2L	Q14157	ubiquitin associated protein 2 like	1,92	1,70	FAIL
HIP1R	O75146	huntingtin interacting protein 1 related	1,92	2,61	FAIL
TJAP1	E2QRK7	tight junction associated protein 1	1,92	2,25	FAIL
C11orf54	E9PIP1	chromosome 11 open reading frame 54	1,92	3,16	FAIL
INPP5K	Q9BT40	inositol polyphosphate-5-phosphatase K	1,92	1,35	FAIL
SPTLC1	O15269	serine palmitoyltransferase long chain base subunit 1	1,92	2,83	FAIL
AGPAT5	Q9NUQ2	1-acylglycerol-3-phosphate O-acyltransferase 5	1,91	1,94	FAIL
THUMPD3	Q9BV44	THUMP domain containing 3	1,91	4,06	FAIL
IRGQ	Q8WZA9	immunity related GTPase Q	1,91	3,00	FAIL
TOR1AIP1	J3KN66	torsin 1A interacting protein 1	1,91	2,93	FAIL
EHD4	Q9H223	EH domain containing 4	1,91	2,28	FAIL
GSK3B	P49841	glycogen synthase kinase 3 beta	1,91	3,16	FAIL
INTS5	Q6P9B9	integrator complex subunit 5	1,91	3,83	FAIL
UBE2S	Q16763	ubiquitin conjugating enzyme E2 S	1,91	1,32	FAIL
ARID2	Q68CP9	AT-rich interaction domain 2	1,90	4,42	FAIL
ZMYND8	A0A087WYS3	zinc finger MYND-type containing 8	1,90	1,35	FAIL
MTA1	E7ESY4	metastasis associated 1	1,90	2,44	FAIL
MAN1B1	Q9UKM7	mannosidase alpha class 1B member 1	1,90	2,33	FAIL
SOX12	O15370	SRY-box transcription factor 12	1,90	1,85	FAIL
NAA10	P41227	N-alpha-acetyltransferase 10, NatA catalytic subunit	1,90	2,32	FAIL
CEP350	Q5VT06	centrosomal protein 350	1,90	1,48	FAIL
PSMD10	B1AJY5	proteasome 26S subunit, non-ATPase 10	1,90	1,67	FAIL
CERS6	Q6ZMG9	ceramide synthase 6	1,90	3,91	FAIL
LRP8	Q14114	LDL receptor related protein 8	1,90	3,35	FAIL
PTPN2	P17706	protein tyrosine phosphatase non-receptor type 2	1,90	2,17	FAIL
VPS26B	Q4G0F5	VPS26 retromer complex component B	1,90	2,15	FAIL
NOL9	Q5SY16	nucleolar protein 9	1,89	3,43	FAIL
SARS2	M0QWZ7	seryl-tRNA synthetase 2, mitochondrial	1,89	1,92	FAIL
ADH5	P11766	alcohol dehydrogenase 5 (class III), chi polypeptide	1,89	2,02	FAIL
RDH11	Q8TC12	retinol dehydrogenase 11	1,89	3,35	FAIL
TAF10	Q12962	TATA-box binding protein associated factor 10	1,89	1,94	FAIL
MINK1	Q8N4C8	misshapen like kinase 1	1,89	2,18	FAIL
HARS1	A0A6Q8PHA5	histidyl-tRNA synthetase 1	1,89	1,54	FAIL
PDHX	O00330	pyruvate dehydrogenase complex component X	1,89	3,91	FAIL

STT3B	Q8TCJ2	STT3 oligosaccharyltransferase complex catalytic subunit B	1,89	4,56	FAIL
UBE2G2	P60604	ubiquitin conjugating enzyme E2 G2	1,89	2,96	FAIL
ABCC1	P33527	ATP binding cassette subfamily C member 1 (ABCC1 blood group)	1,89	2,39	FAIL
PSMA5	P28066	proteasome 20S subunit alpha 5	1,89	1,37	FAIL
ELOB	Q15370	elongin B	1,89	2,61	FAIL
CTCF	A0A2R8Y595	CCCTC-binding factor	1,89	1,85	FAIL
AAK1	A0A096LP25	AP2 associated kinase 1	1,89	2,11	FAIL
ASS1	P00966	argininosuccinate synthase 1	1,89	2,22	FAIL
STAT1	P42224	signal transducer and activator of transcription 1	1,89	2,05	FAIL
TLR3	O15455	toll like receptor 3	1,89	1,64	FAIL
TRUB1	Q8WWH5	TruB pseudouridine synthase family member 1	1,89	3,02	FAIL
	A0A087WZ13		1,89	1,65	FAIL
UBXN4	Q92575	UBX domain protein 4	1,89	1,34	FAIL
HSP90AB1	P08238	heat shock protein 90 alpha family class B member 1	1,89	2,91	FAIL
CMSS1	C9J384	cms1 ribosomal small subunit homolog	1,89	2,76	FAIL
TSC2	A0A2R8YDR3	TSC complex subunit 2	1,89	3,77	FAIL
MRPL23	Q16540	mitochondrial ribosomal protein L23	1,89	3,30	FAIL
RAB9A	P51151	RAB9A, member RAS oncogene family	1,89	1,44	FAIL
DCAF7	P61962	DDB1 and CUL4 associated factor 7	1,89	3,98	FAIL
VPS4A	Q9UN37	vacuolar protein sorting 4 homolog A	1,89	2,78	FAIL
EEF1E1	O43324	eukaryotic translation elongation factor 1 epsilon 1	1,89	1,91	FAIL
CNOT3	O75175	CCR4-NOT transcription complex subunit 3	1,89	1,89	FAIL
CSE1L	P55060	chromosome segregation 1 like	1,88	1,75	FAIL
COTL1	Q14019	coactosin like F-actin binding protein 1	1,88	2,99	FAIL
SGO1	Q5FBB7	shugoshin 1	1,88	3,72	FAIL
RBM26	Q5T8P6	RNA binding motif protein 26	1,88	3,49	FAIL
NDUFB10	O96000	NADH:ubiquinone oxidoreductase subunit B10	1,88	2,42	FAIL
RCC2	Q9P258	regulator of chromosome condensation 2	1,88	2,47	FAIL
CCDC120	Q96HB5	coiled-coil domain containing 120	1,88	1,96	FAIL
TAF1	P21675	TATA-box binding protein associated factor 1	1,88	1,63	FAIL
HOMER3	Q9NSC5	homer scaffold protein 3	1,88	1,61	FAIL
PGM3	A0A494C0G1	phosphoglucomutase 3	1,88	1,41	FAIL
BCAP31	C9JSP1	B cell receptor associated protein 31	1,88	2,80	FAIL
NUFIP2	Q7Z417	nuclear FMR1 interacting protein 2	1,88	3,05	FAIL
XYLT1	Q86Y38	xylosyltransferase 1	1,88	1,95	FAIL
SMAD2	Q15796	SMAD family member 2	1,88	1,82	FAIL
TRIM26	Q12899	tripartite motif containing 26	1,88	2,28	FAIL
	A0A0J9YYD9		1,88	1,71	FAIL
SMC5	Q8IY18	structural maintenance of chromosomes 5	1,88	2,22	FAIL
ELF1	A0A0U1RRL5	E74 like ETS transcription factor 1	1,87	2,65	FAIL
IPO13	O94829	importin 13	1,87	2,30	FAIL
KPNA1	P52294	karyopherin subunit alpha 1	1,87	2,62	FAIL
SCAMP2	O15127	secretory carrier membrane protein 2	1,87	1,85	FAIL
PCK2	Q16822	phosphoenolpyruvate carboxykinase 2, mitochondrial	1,87	1,68	FAIL

RAB35	Q15286	RAB35, member RAS oncogene family	1,87	4,01	FAIL
SACM1L	A0A5F9ZHN7	SAC1 like phosphatidylinositide phosphatase	1,87	2,59	FAIL
	A0A087WY61		1,87	3,44	FAIL
KDM6A	O15550	lysine demethylase 6A	1,87	2,79	FAIL
PRKAG1	P54619	protein kinase AMP-activated non-catalytic subunit gamma 1	1,87	2,77	FAIL
NIFK	Q9BYG3	nucleolar protein interacting with the FHA domain of MKI67	1,87	2,88	FAIL
HDAC2	Q92769	histone deacetylase 2	1,87	1,68	FAIL
MYL6	B7Z6Z4	myosin light chain 6	1,87	2,89	FAIL
TCF12	Q99081	transcription factor 12	1,87	2,04	FAIL
PROX1	Q92786	prospero homeobox 1	1,87	2,04	FAIL
LDAF1	Q96B96	lipid droplet assembly factor 1	1,87	3,65	FAIL
EP300	Q09472	E1A binding protein p300	1,87	2,84	FAIL
DGKZ	Q13574	diacylglycerol kinase zeta	1,87	4,09	FAIL
ABCF3	Q9NUQ8	ATP binding cassette subfamily F member 3	1,86	1,49	FAIL
RPS6KB2	Q9UBS0	ribosomal protein S6 kinase B2	1,86	4,24	FAIL
RPS12	P25398	ribosomal protein S12	1,86	3,99	FAIL
OLA1	Q9NTK5	Obg like ATPase 1	1,86	2,32	FAIL
MIOS	Q9NXC5	meiosis regulator for oocyte development	1,86	2,33	FAIL
MED8	Q96G25	mediator complex subunit 8	1,86	1,48	FAIL
HSP90AA1	P07900	heat shock protein 90 alpha family class A member 1	1,86	3,81	FAIL
LARS2	Q15031	leucyl-tRNA synthetase 2, mitochondrial	1,86	2,50	FAIL
EXD2	Q9NVH0	exonuclease 3'-5' domain containing 2	1,86	1,51	FAIL
MASTL	Q96GX5	microtubule associated serine/threonine kinase like	1,85	2,51	FAIL
PPP2R5E	Q16537	protein phosphatase 2 regulatory subunit B'epsilon	1,85	1,47	FAIL
SYNJ1	O43426	synaptojanin 1	1,85	2,42	FAIL
PSMC2	P35998	proteasome 26S subunit, ATPase 2	1,85	2,49	FAIL
IQGAP2	Q13576	IQ motif containing GTPase activating protein 2	1,85	3,12	FAIL
SNX25	Q9H3E2	sorting nexin 25	1,85	1,55	FAIL
PANK4	E9PHT6	pantothenate kinase 4 (inactive)	1,85	3,35	FAIL
BIVM-ERCC5	R4GMW8	BIVM-ERCC5 readthrough	1,85	1,56	FAIL
EIF2A	Q9BY44	eukaryotic translation initiation factor 2A	1,85	2,72	FAIL
LRRC47	Q8N1G4	leucine rich repeat containing 47	1,85	3,25	FAIL
TRPC3	J3QTB0	transient receptor potential cation channel subfamily C member 3	1,85	1,72	FAIL
SKP2	A0A0A0MTL5	S-phase kinase associated protein 2	1,85	3,45	FAIL
RCOR1	Q9UKL0	REST corepressor 1	1,85	1,65	FAIL
TFB2M	Q9H5Q4	transcription factor B2, mitochondrial	1,85	1,92	FAIL
RPGR	A0A2R8YFT6	retinitis pigmentosa GTPase regulator	1,85	1,37	FAIL
CCT7	Q99832	chaperonin containing TCP1 subunit 7	1,85	1,86	FAIL
PML	P29590	PML nuclear body scaffold	1,85	4,58	FAIL
AP2A1	O95782	adaptor related protein complex 2 subunit alpha 1	1,85	3,68	FAIL
MICU2	Q8IYU8	mitochondrial calcium uptake 2	1,85	2,12	FAIL
	M0QZD8		1,84	3,50	FAIL
DYNLL1	P63167	dynein light chain LC8-type 1	1,84	4,82	FAIL
TM7SF2	H0YEN6	transmembrane 7 superfamily member 2	1,84	1,78	FAIL

VPS29	F8VXU5	VPS29 retromer complex component	1,84	2,63	FAIL
RPTOR	Q8N122	regulatory associated protein of MTOR complex 1	1,84	2,76	FAIL
AHCY	P23526	adenosylhomocysteinase	1,84	4,00	FAIL
TM9SF3	Q9HD45	transmembrane 9 superfamily member 3	1,84	3,01	FAIL
NUDCD2	Q8WVJ2	NudC domain containing 2	1,84	3,54	FAIL
G3BP2	Q9UN86	G3BP stress granule assembly factor 2	1,84	1,93	FAIL
CPNE3	O75131	copine 3	1,84	1,98	FAIL
PSAT1	Q9Y617	phosphoserine aminotransferase 1	1,84	2,11	FAIL
SMPD4	Q9NXE4	sphingomyelin phosphodiesterase 4	1,84	2,65	FAIL
CEP250	Q9BV73	centrosomal protein 250	1,84	2,01	FAIL
TUBB3	Q13509	tubulin beta 3 class III	1,84	1,95	FAIL
SRPK1	Q96SB4	SRSF protein kinase 1	1,83	3,82	FAIL
CLPTM1L	Q96KA5	CLPTM1 like	1,83	1,61	FAIL
VAV1	F5H5P4	vav guanine nucleotide exchange factor 1	1,83	3,07	FAIL
COX15	Q7KZN9	cytochrome c oxidase assembly homolog COX15	1,83	1,97	FAIL
UMPS	P11172	uridine monophosphate synthetase	1,83	1,42	FAIL
CTNNBL1	Q8WYA6	catenin beta like 1	1,83	3,37	FAIL
GTF2B	Q00403	general transcription factor IIB	1,83	1,46	FAIL
LMO7	E9PMT2	LIM domain 7	1,83	1,42	FAIL
ARHGEF1	M0QZR4	Rho guanine nucleotide exchange factor 1	1,83	1,94	FAIL
MAPK1	P28482	mitogen-activated protein kinase 1	1,83	3,36	FAIL
TRIR	Q9BQ61	telomerase RNA component interacting RNase	1,83	1,69	FAIL
VPS50	Q96JG6	VPS50 subunit of EARP/GARPII complex	1,83	1,66	FAIL
ATP1B3	P54709	ATPase Na+/K+ transporting subunit beta 3	1,83	2,13	FAIL
PI4K2A	Q9BTU6	phosphatidylinositol 4-kinase type 2 alpha	1,83	1,92	FAIL
MPO	P05164	myeloperoxidase	1,83	1,67	FAIL
TRAPPC8	J3KSL8	trafficking protein particle complex subunit 8	1,83	2,38	FAIL
MAGEA4	P43358	MAGE family member A4	1,83	1,96	FAIL
CORO1A	P31146	coronin 1A	1,83	2,12	FAIL
MSL3	A0A3B3IT59	MSL complex subunit 3	1,82	2,82	FAIL
GPS2	I3L3Y9	G protein pathway suppressor 2	1,82	3,55	FAIL
CDK6	Q00534	cyclin dependent kinase 6	1,82	2,23	FAIL
CLCN7	P51798	chloride voltage-gated channel 7	1,82	2,69	FAIL
FOXRED1	Q96CU9	FAD dependent oxidoreductase domain containing 1	1,82	2,69	FAIL
FAM167B	Q9BTA0	family with sequence similarity 167 member B	1,82	3,46	FAIL
RRP15	Q9Y3B9	ribosomal RNA processing 15 homolog	1,82	1,40	FAIL
PRKCQ	Q04759	protein kinase C theta	1,82	2,24	FAIL
PTGES3	A0A087WYT3	prostaglandin E synthase 3	1,82	1,95	FAIL
UBE2N	F8VZ29	ubiquitin conjugating enzyme E2 N	1,82	2,08	FAIL
INPP5F	A0A3B3ISI3	inositol polyphosphate-5-phosphatase F	1,82	1,39	FAIL
NDUFB4	O95168	NADH:ubiquinone oxidoreductase subunit B4	1,82	1,96	FAIL
RAB43	Q86YS6	RAB43, member RAS oncogene family	1,82	3,13	FAIL
STK26	Q8NBY1	serine/threonine kinase 26	1,82	2,68	FAIL
COG6	Q9Y2V7	component of oligomeric golgi complex 6	1,82	3,02	FAIL
TUBB3	G3V542	tubulin beta 3 class III	1,82	1,89	FAIL
	A0A0U1RRH6		1,82	4,03	FAIL
SLC25A25	Q6KCM7	solute carrier family 25 member 25	1,82	1,69	FAIL
PIP5K1A	A6PW57	phosphatidylinositol-4-phosphate 5-kinase type 1 alpha	1,82	3,61	FAIL

MARK3	J3KNR0	microtubule affinity regulating kinase 3	1,82	2,86	FAIL
ARMC9	A0A2Q3DP09	armadillo repeat containing 9	1,82	3,21	FAIL
GSK3A	P49840	glycogen synthase kinase 3 alpha	1,82	3,54	FAIL
LMBRD2	Q68DH5	LMBR1 domain containing 2	1,82	2,60	FAIL
HPS6	Q86YV9	HPS6 biogenesis of lysosomal organelles complex 2 subunit 3	1,81	2,22	FAIL
CSNK1D	H7BYT1	casein kinase 1 delta	1,81	4,21	FAIL
SEC61B	P60468	SEC61 translocon subunit beta	1,81	2,87	FAIL
MED1	Q15648	mediator complex subunit 1	1,81	2,91	FAIL
POC5	Q8NA72	POC5 centriolar protein	1,81	2,74	FAIL
PRAME	P78395	PRAME nuclear receptor transcriptional regulator	1,81	1,30	FAIL
DNAJC11	Q9NVH1	DnaJ heat shock protein family (Hsp40) member C11	1,81	1,78	FAIL
RPS21	P63220	ribosomal protein S21	1,81	1,34	FAIL
USP3	Q9Y6I4	ubiquitin specific peptidase 3	1,81	2,99	FAIL
ABL1	P00519	ABL proto-oncogene 1, non-receptor tyrosine kinase	1,81	2,11	FAIL
CUL3	Q13618	cullin 3	1,81	2,18	FAIL
MAPK9	P45984	mitogen-activated protein kinase 9	1,81	2,73	FAIL
UTP14A	Q9BVJ6	UTP14A small subunit processome component	1,81	1,34	FAIL
SMARCAL1	Q9NZC9	SWI/SNF related, matrix associated, actin dependent regulator of chromatin, subfamily a like 1	1,81	1,39	FAIL
CSNK1A1	U3KPX3	casein kinase 1 alpha 1	1,81	2,91	FAIL
KIF5B	P33176	kinesin family member 5B	1,81	2,58	FAIL
KLC1	G3V3H3	kinesin light chain 1	1,81	3,80	FAIL
CUX1	P39880	cut like homeobox 1	1,81	1,41	FAIL
NVL	O15381	nuclear VCP like	1,81	2,47	FAIL
SH2D3C	Q8N5H7	SH2 domain containing 3C	1,80	3,40	FAIL
RIF1	Q5UIP0	replication timing regulatory factor 1	1,80	1,76	FAIL
AUP1	Q9Y679	AUP1 lipid droplet regulating VLDL assembly factor	1,80	2,03	FAIL
LCMT2	O60294	leucine carboxyl methyltransferase 2	1,80	2,79	FAIL
TOP2B	Q02880	DNA topoisomerase II beta	1,80	3,71	FAIL
CERT1	A0A2R8YEK8	ceramide transporter 1	1,80	1,76	FAIL
MYO1B	E9PDF6	myosin IB	1,80	1,87	FAIL
CLPX	O76031	caseinolytic mitochondrial matrix peptidase chaperone subunit X	1,80	2,77	FAIL
DTYMK	P23919	deoxythymidylate kinase	1,80	2,06	FAIL
UBR4	Q5T4S7	ubiquitin protein ligase E3 component n-recognin 4	1,80	4,46	FAIL
	A0A0A0MS59		1,80	1,86	FAIL
KDM1A	O60341	lysine demethylase 1A	1,80	1,40	FAIL
OPTC	Q9UBM4	opticin	1,80	1,65	FAIL
EIF4G1	E7EUU4	eukaryotic translation initiation factor 4 gamma 1	1,80	2,39	FAIL
	A0A0A0MS54		1,80	1,70	FAIL
TBC1D5	Q92609	TBC1 domain family member 5	1,80	2,56	FAIL
ARF5	P84085	ADP ribosylation factor 5	1,80	1,75	FAIL
PHB1	P35232	prohibitin 1	1,80	3,54	FAIL
	A2BF26		1,79	2,56	FAIL
ELOC	Q15369	elongin C	1,79	4,09	FAIL

PDK1	Q15118	pyruvate dehydrogenase kinase 1	1,79	3,15	FAIL
LIG1	P18858	DNA ligase 1	1,79	2,59	FAIL
XPO5	Q9HAV4	exportin 5	1,79	2,55	FAIL
CLPB	Q9H078	ClpB family mitochondrial disaggregase	1,79	2,09	FAIL
ANAPC5	Q9UJX4	anaphase promoting complex subunit 5	1,79	3,08	FAIL
NFXL1	Q6ZNB6	nuclear transcription factor, X-box binding like 1	1,79	2,46	FAIL
RAB21	Q9UL25	RAB21, member RAS oncogene family	1,79	1,72	FAIL
KIRREL1	Q96J84	kirre like nephrin family adhesion molecule 1	1,79	2,61	FAIL
	P51784		1,79	2,60	FAIL
CHMP7	Q8WUX9	charged multivesicular body protein 7	1,79	1,77	FAIL
ACSF3	Q4G176	acyl-CoA synthetase family member 3	1,79	3,50	FAIL
RAB33B	A0A494C0Z5	RAB33B, member RAS oncogene family	1,78	2,13	FAIL
LCLAT1	Q6UWP7	lysocardiolipin acyltransferase 1	1,78	1,75	FAIL
SND1	Q7KZF4	staphylococcal nuclease and tudor domain containing 1	1,78	3,02	FAIL
LIG4	A0A0C4DGV9	DNA ligase 4	1,78	2,13	FAIL
TAP1	Q03518	transporter 1, ATP binding cassette subfamily B member	1,78	2,50	FAIL
AIMP1	Q12904	aminoacyl tRNA synthetase complex interacting multifunctional protein 1	1,78	1,58	FAIL
SCAMP1	A0A087WXB0	secretory carrier membrane protein 1	1,78	1,92	FAIL
ST13	P50502	ST13 Hsp70 interacting protein	1,78	3,21	FAIL
MIA3	Q5JRA6	MIA SH3 domain ER export factor 3	1,78	1,39	FAIL
MED23	Q9ULK4	mediator complex subunit 23	1,78	3,37	FAIL
EIF2S3	P41091	eukaryotic translation initiation factor 2 subunit gamma	1,78	2,40	FAIL
MAP2K6	P52564	mitogen-activated protein kinase kinase 6	1,77	2,85	FAIL
MICOS13	A0A140TA86	mitochondrial contact site and cristae organizing system subunit 13	1,77	1,67	FAIL
	F6S7C4		1,77	3,66	FAIL
ZNF22	P17026	zinc finger protein 22	1,77	1,64	FAIL
USP7	Q93009	ubiquitin specific peptidase 7	1,77	3,67	FAIL
VAV3	Q9UKW4	vav guanine nucleotide exchange factor 3	1,77	3,11	FAIL
TUT4	A0A0C4DFM7	terminal uridylyl transferase 4	1,77	3,16	FAIL
KDM4B	O94953	lysine demethylase 4B	1,77	2,04	FAIL
NHERF1	O14745	NHERF family PDZ scaffold protein 1	1,77	2,95	FAIL
EDC3	Q96F86	enhancer of mRNA decapping 3	1,77	2,64	FAIL
SPCS1	A0A5F9YFS9	signal peptidase complex subunit 1	1,77	2,20	FAIL
PRKAA1	Q13131	protein kinase AMP-activated catalytic subunit alpha 1	1,77	3,32	FAIL
	A8MPP1		1,77	2,28	FAIL
STAT6	A0A1W2PNW1	signal transducer and activator of transcription 6	1,77	2,69	FAIL
RNF123	Q5XPI4	ring finger protein 123	1,77	3,19	FAIL
EIF4E	D6RBW1	eukaryotic translation initiation factor 4E	1,77	2,25	FAIL
HACD3	Q9P035	3-hydroxyacyl-CoA dehydratase 3	1,77	1,69	FAIL
TBL1XR1	Q9BZK7	TBL1X/Y related 1	1,77	3,45	FAIL
SLC33A1	O00400	solute carrier family 33 member 1	1,76	2,66	FAIL
MRPL41	Q8IXM3	mitochondrial ribosomal protein L41	1,76	4,00	FAIL
POLR2C	P19387	RNA polymerase II subunit C	1,76	3,42	FAIL
SLFN11	Q7Z7L1	schlafen family member 11	1,76	1,59	FAIL

C2CD5	Q86YS7	C2 calcium dependent domain containing 5	1,76	1,67	FAIL
SDHB	P21912	succinate dehydrogenase complex iron sulfur subunit B	1,76	1,61	FAIL
TMEM126B	H0YD74	transmembrane protein 126B	1,76	2,06	FAIL
SMARCB1	G5E975	SWI/SNF related, matrix associated, actin dependent regulator of chromatin, subfamily b, member 1	1,76	1,88	FAIL
ARMC1	Q9NVT9	armadillo repeat containing 1	1,76	1,69	FAIL
SPTY2D1	Q68D10	SPT2 chromatin protein domain containing 1	1,76	3,11	FAIL
MBD4	O95243	methyl-CpG binding domain 4, DNA glycosylase	1,76	2,74	FAIL
MRPS14	O60783	mitochondrial ribosomal protein S14	1,76	1,64	FAIL
RFT1	B5MDE0	RFT1 homolog	1,75	2,68	FAIL
SEC24A	O95486	SEC24 homolog A, COPII coat complex component	1,75	3,55	FAIL
RBM34	H7C5M1	RNA binding motif protein 34	1,75	1,69	FAIL
TRIP13	Q15645	thyroid hormone receptor interactor 13	1,75	2,09	FAIL
DHX15	O43143	DEAH-box helicase 15	1,75	2,79	FAIL
SLC2A1	P11166	solute carrier family 2 member 1	1,75	3,22	FAIL
TNPO3	Q9Y5L0	transportin 3	1,75	2,24	FAIL
PTDSS2	Q9BVG9	phosphatidylserine synthase 2	1,75	1,72	FAIL
ATP8	P03928	ATP synthase F0 subunit 8	1,75	4,07	FAIL
ECH1	Q13011	enoyl-CoA hydratase 1	1,75	1,34	FAIL
CDKAL1	Q5VV42	CDK5 regulatory subunit associated protein 1 like 1	1,75	2,70	FAIL
HNRNPH2	P55795	heterogeneous nuclear ribonucleoprotein H2	1,75	1,41	FAIL
NELFA	A0A0C4DFX9	negative elongation factor complex member A	1,74	2,42	FAIL
RTN3	O95197	reticulon 3	1,74	3,11	FAIL
MTDH	Q86UE4	metadherin	1,74	1,81	FAIL
ZNF326	Q5BKZ1	zinc finger protein 326	1,74	2,31	FAIL
MGMT	B4DEE8	O-6-methylguanine-DNA methyltransferase	1,74	2,77	FAIL
FMNL1	O95466	formin like 1	1,74	4,37	FAIL
METAP1	P53582	methionyl aminopeptidase 1	1,74	2,09	FAIL
CCDC25	Q86WR0	coiled-coil domain containing 25	1,74	2,66	FAIL
RAP1B	P61224	RAP1B, member of RAS oncogene family	1,74	1,94	FAIL
CDK9	P50750	cyclin dependent kinase 9	1,74	2,63	FAIL
GANAB	Q14697	glucosidase II alpha subunit	1,74	1,96	FAIL
CCAR1	Q8IX12	cell division cycle and apoptosis regulator 1	1,74	1,45	FAIL
SLC27A4	Q6P1M0	solute carrier family 27 member 4	1,73	3,00	FAIL
PMPCB	O75439	peptidase, mitochondrial processing subunit beta	1,73	2,54	FAIL
CD3E	E9PSH8	CD3 epsilon subunit of T-cell receptor complex	1,73	1,93	FAIL
NEU3	Q9UQ49	neuraminidase 3	1,73	3,05	FAIL
COPB1	P53618	COPI coat complex subunit beta 1	1,73	3,23	FAIL
RCE1	Q9Y256	Ras converting CAAX endopeptidase 1	1,73	1,82	FAIL
UBE2D3	P61077	ubiquitin conjugating enzyme E2 D3	1,73	4,16	FAIL
MEAF6	Q9HAF1	MYST/Esa1 associated factor 6	1,73	2,80	FAIL
GATAD2B	Q8WXI9	GATA zinc finger domain containing 2B	1,73	3,20	FAIL
LPCAT1	Q8NF37	lysophosphatidylcholine acyltransferase 1	1,73	2,18	FAIL
TUBA1B	P68363	tubulin alpha 1b	1,73	2,09	FAIL
PMS2	P54278	PMS1 homolog 2, mismatch repair system component	1,73	1,42	FAIL
ODAD3	A5D8V7	outer dynein arm docking complex subunit 3	1,72	2,33	FAIL
ITPA	Q9BY32	inosine triphosphatase	1,72	1,48	FAIL

SMG1	Q96Q15	SMG1 nonsense mediated mRNA decay associated PI3K related kinase	1,72	2,39	FAIL
INTS4	Q96HW7	integrator complex subunit 4	1,72	1,83	FAIL
GIMAP1	Q8WWP7	GTPase, IMAP family member 1	1,72	2,06	FAIL
BST2	Q10589	bone marrow stromal cell antigen 2	1,72	2,73	FAIL
VDAC2	P45880	voltage dependent anion channel 2	1,72	2,24	FAIL
UNC45A	A0A1W2PNX8	unc-45 myosin chaperone A	1,72	2,94	FAIL
PSMD12	O00232	proteasome 26S subunit, non-ATPase 12	1,72	1,84	FAIL
HVCN1	Q96D96	hydrogen voltage gated channel 1	1,72	1,37	FAIL
COMMD3-BMI1	R4GMX3	COMMD3-BMI1 readthrough	1,72	2,03	FAIL
RPS24	A0A2R8Y849	ribosomal protein S24	1,71	1,82	FAIL
RB1	P06400	RB transcriptional corepressor 1	1,71	2,71	FAIL
SRP54	P61011	signal recognition particle 54	1,71	1,96	FAIL
TCF3	P15923	transcription factor 3	1,71	1,39	FAIL
CUL2	Q13617	cullin 2	1,71	2,13	FAIL
GTF3C1	Q12789	general transcription factor IIIC subunit 1	1,71	1,46	FAIL
PGLS	O95336	6-phosphogluconolactonase	1,71	2,43	FAIL
EARS2	Q5JPH6	glutamyl-tRNA synthetase 2, mitochondrial	1,71	1,86	FAIL
TAF12	Q16514	TATA-box binding protein associated factor 12	1,71	2,20	FAIL
PRPF3	O43395	pre-mRNA processing factor 3	1,71	1,90	FAIL
YTHDF1	Q9BYJ9	YTH N6-methyladenosine RNA binding protein F1	1,70	1,94	FAIL
NUDC	Q9Y266	nuclear distribution C, dynein complex regulator	1,70	4,30	FAIL
HMCES	E7EMP6	5-hydroxymethylcytosine binding, ES cell specific	1,70	3,75	FAIL
DYNLRB2	Q8TF09	dynein light chain roadblock-type 2	1,70	1,73	FAIL
HM13	A0A3B3IUB5	histocompatibility minor 13	1,70	2,42	FAIL
DIDO1	Q9BTC0	death inducer-obliterator 1	1,70	1,66	FAIL
LAMA3	A0A0A0MSA0	laminin subunit alpha 3	1,70	2,69	FAIL
TMEM97	Q5BJF2	transmembrane protein 97	1,70	2,79	FAIL
KIF2C	Q99661	kinesin family member 2C	1,70	2,02	FAIL
ZMYM2	Q9UBW7	zinc finger MYM-type containing 2	1,70	4,21	FAIL
MTCH2	Q9Y6C9	mitochondrial carrier 2	1,70	2,02	FAIL
HSPA9	P38646	heat shock protein family A (Hsp70) member 9	1,70	3,25	FAIL
DLGAP4	Q9Y2H0	DLG associated protein 4	1,70	4,09	FAIL
RIC8A	Q9NPQ8	RIC8 guanine nucleotide exchange factor A	1,70	2,66	FAIL
ARHGAP19	Q14CB8	Rho GTPase activating protein 19	1,70	3,68	FAIL
ARFIP2	P53365	ADP ribosylation factor interacting protein 2	1,70	2,70	FAIL
RPS15A	P62244	ribosomal protein S15a	1,70	2,05	FAIL
SF3B6	Q9Y3B4	splicing factor 3b subunit 6	1,70	1,36	FAIL
MRPS12	O15235	mitochondrial ribosomal protein S12	1,70	2,76	FAIL
BCL11B	Q9C0K0	BCL11 transcription factor B	1,70	1,92	FAIL
CHAF1A	Q13111	chromatin assembly factor 1 subunit A	1,70	2,31	FAIL
ALMS1	Q8TCU4	ALMS1 centrosome and basal body associated protein	1,69	3,06	FAIL
SLC35F2	Q8IXU6	solute carrier family 35 member F2	1,69	4,26	FAIL
UBAC2	Q8NBM4	UBA domain containing 2	1,69	3,22	FAIL
CTBP1	Q13363	C-terminal binding protein 1	1,69	2,45	FAIL
IDE	P14735	insulin degrading enzyme	1,69	2,28	FAIL
	A0A087WZT0		1,69	2,00	FAIL

DNAJC13	O75165	DnaJ heat shock protein family (Hsp40) member C13	1,69	3,43	FAIL
SMYD3	Q9H7B4	SET and MYND domain containing 3	1,69	1,70	FAIL
PRC1	O43663	protein regulator of cytokinesis 1	1,69	1,61	FAIL
TMCO1	Q9UM00	transmembrane and coiled-coil domains 1	1,69	3,03	FAIL
LMNB2	Q03252	lamin B2	1,69	3,12	FAIL
NPC1	O15118	NPC intracellular cholesterol transporter 1	1,69	3,89	FAIL
RDH14	Q9HBH5	retinol dehydrogenase 14	1,69	1,57	FAIL
FAF2	Q96CS3	Fas associated factor family member 2	1,69	2,31	FAIL
CYBC1	H0Y2X1	cytochrome b-245 chaperone 1	1,69	3,12	FAIL
STAG1	Q8WVM7	STAG1 cohesin complex component	1,68	2,01	FAIL
PPM1E	Q8WY54	protein phosphatase, Mg2+/Mn2+ dependent 1E	1,68	1,96	FAIL
CCDC124	Q96CT7	coiled-coil domain containing 124	1,68	1,58	FAIL
SATB1	Q01826	SATB homeobox 1	1,68	3,51	FAIL
KIF23	Q02241	kinesin family member 23	1,68	1,43	FAIL
HSPA8	P11142	heat shock protein family A (Hsp70) member 8	1,68	1,99	FAIL
THYN1	Q9P016	thymocyte nuclear protein 1	1,68	1,57	FAIL
PTPN1	P18031	protein tyrosine phosphatase non-receptor type 1	1,68	2,18	FAIL
POLDIP2	Q9Y2S7	DNA polymerase delta interacting protein 2	1,68	1,64	FAIL
PPIA	P62937	peptidylprolyl isomerase A	1,68	2,43	FAIL
IP6K1	Q92551	inositol hexakisphosphate kinase 1	1,68	1,60	FAIL
YKT6	O15498	YKT6 v-SNARE homolog	1,68	2,62	FAIL
ARHGAP9	Q9BRR9	Rho GTPase activating protein 9	1,68	1,97	FAIL
	A3KFL1		1,68	2,25	FAIL
CD47	Q08722	CD47 molecule	1,68	3,25	FAIL
MED14	O60244	mediator complex subunit 14	1,68	1,89	FAIL
PPP1CC	P36873	protein phosphatase 1 catalytic subunit gamma	1,68	3,94	FAIL
GMFG	O60234	glia maturation factor gamma	1,68	3,54	FAIL
TSC1	A0A2R8Y5N2	TSC complex subunit 1	1,68	3,19	FAIL
NUMA1	Q14980	nuclear mitotic apparatus protein 1	1,68	3,46	FAIL
POLR2L	P62875	RNA polymerase II, I and III subunit L	1,68	3,14	FAIL
SFXN1	Q9H9B4	sideroflexin 1	1,68	1,42	FAIL
AKT2	P31751	AKT serine/threonine kinase 2	1,68	3,38	FAIL
IPO11	Q9UI26	importin 11	1,68	1,70	FAIL
SLC16A3	O15427	solute carrier family 16 member 3	1,67	2,52	FAIL
DROSHA	Q9NRR4	drosha ribonuclease III	1,67	1,64	FAIL
UBR5	O95071	ubiquitin protein ligase E3 component n-recognin 5	1,67	1,77	FAIL
RPS6KA3	B1AXG1	ribosomal protein S6 kinase A3	1,67	2,95	FAIL
XRCC5	P13010	X-ray repair cross complementing 5	1,67	3,60	FAIL
ATL3	Q6DD88	atlastin GTPase 3	1,67	2,02	FAIL
KIF2A	A0A6Q8PFA6	kinesin family member 2A	1,67	3,91	FAIL
USP8	P40818	ubiquitin specific peptidase 8	1,67	1,54	FAIL
XRCC3	G3V399	X-ray repair cross complementing 3	1,67	1,61	FAIL
UTP15	Q8TED0	UTP15 small subunit processome component	1,67	2,73	FAIL
SRPRB	Q9Y5M8	SRP receptor subunit beta	1,67	3,32	FAIL
	Q9UJX3		1,67	2,64	FAIL
	A0A0A0MSJ9		1,67	2,29	FAIL
HNRNPH3	P31942	heterogeneous nuclear ribonucleoprotein H3	1,67	2,30	FAIL
KLC4	Q9NSK0	kinesin light chain 4	1,67	1,71	FAIL

TRMT2A	Q8IZ69	tRNA methyltransferase 2 homolog A	1,67	1,73	FAIL
ACSL3	O95573	acyl-CoA synthetase long chain family member 3	1,67	3,04	FAIL
ESPL1	Q14674	extra spindle pole bodies like 1, separase	1,67	2,70	FAIL
COPA	P53621	COPI coat complex subunit alpha	1,66	3,82	FAIL
SLC25A20	O43772	solute carrier family 25 member 20	1,66	2,64	FAIL
XPO1	O14980	exportin 1	1,66	3,03	FAIL
YARS1	P54577	tyrosyl-tRNA synthetase 1	1,66	1,93	FAIL
RPUSD3	Q6P087	RNA pseudouridine synthase D3	1,66	1,49	FAIL
NUP133	Q8WUM0	nucleoporin 133	1,66	1,64	FAIL
TUBB	P07437	tubulin beta class I	1,66	1,80	FAIL
DDX39A	O00148	DExD-box helicase 39A	1,66	1,40	FAIL
CLP1	Q92989	cleavage factor polyribonucleotide kinase subunit 1	1,66	1,96	FAIL
XRN1	Q8IZH2	5'-3' exoribonuclease 1	1,66	1,99	FAIL
RESF1	Q9HCM1	retroelement silencing factor 1	1,66	1,80	FAIL
INTS3	Q68E01	integrator complex subunit 3	1,66	2,91	FAIL
DARS1	P14868	aspartyl-tRNA synthetase 1	1,66	2,51	FAIL
WDR44	Q5JSH3	WD repeat domain 44	1,66	3,05	FAIL
BRPF1	P55201	bromodomain and PHD finger containing 1	1,66	3,09	FAIL
BTBD2	Q9BX70	BTB domain containing 2	1,66	2,39	FAIL
ATP11B	Q9Y2G3	ATPase phospholipid transporting 11B (putative)	1,65	1,84	FAIL
NARS2	Q96I59	asparaginyl-tRNA synthetase 2, mitochondrial	1,65	1,48	FAIL
	H0YHG0		1,65	2,47	FAIL
PDLIM5	Q96HC4	PDZ and LIM domain 5	1,65	4,27	FAIL
GSE1	Q14687	Gse1 coiled-coil protein	1,65	2,29	FAIL
RAB27A	H3BVH7	RAB27A, member RAS oncogene family	1,65	3,61	FAIL
NCLN	Q969V3	nicalin	1,65	2,32	FAIL
CCDC154	A6NI56	coiled-coil domain containing 154	1,65	2,77	FAIL
TEX55	S4R404	testis expressed 55	1,65	1,62	FAIL
ZFYVE16	Q7Z3T8	zinc finger FYVE-type containing 16	1,65	2,59	FAIL
CHCHD3	C9JRZ6	coiled-coil-helix-coiled-coil-helix domain containing 3	1,65	3,46	FAIL
PURB	Q96QR8	purine rich element binding protein B	1,65	2,31	FAIL
USP36	Q9P275	ubiquitin specific peptidase 36	1,65	2,10	FAIL
STXBP5L	Q9Y2K9	syntaxin binding protein 5L	1,65	2,51	FAIL
NNT	Q13423	nicotinamide nucleotide transhydrogenase	1,65	3,02	FAIL
SPINK5	Q9NQ38	serine peptidase inhibitor Kazal type 5	1,65	1,95	FAIL
TRIM27	P14373	tripartite motif containing 27	1,65	1,80	FAIL
SAR1A	Q9NR31	secretion associated Ras related GTPase 1A	1,65	1,76	FAIL
SLAIN2	Q9P270	SLAIN motif family member 2	1,65	3,03	FAIL
SLC7A5	Q01650	solute carrier family 7 member 5	1,65	1,85	FAIL
CDH18	D6RER2	cadherin 18	1,64	3,47	FAIL
MEPCE	Q7L2J0	methylphosphate capping enzyme	1,64	2,94	FAIL
DUS2	E7EUN9	dihydrouridine synthase 2	1,64	1,92	FAIL
DDX50	Q9BQ39	DExD-box helicase 50	1,64	1,80	FAIL
PCBP2	F8VZX2	poly(rC) binding protein 2	1,64	1,70	FAIL
PFKL	P17858	phosphofructokinase, liver type	1,64	1,69	FAIL
EIF2B5	Q13144	eukaryotic translation initiation factor 2B subunit epsilon	1,64	2,53	FAIL
SPAG7	I3L4C3	sperm associated antigen 7	1,64	1,51	FAIL

ABCD1	P33897	ATP binding cassette subfamily D member 1	1,64	1,40	FAIL
ARF6	P62330	ADP ribosylation factor 6	1,64	1,74	FAIL
ATP5PB	P24539	ATP synthase peripheral stalk-membrane subunit b	1,64	3,22	FAIL
SON	P18583	SON DNA and RNA binding protein	1,64	2,38	FAIL
NIBAN2	Q96TA1	niban apoptosis regulator 2	1,64	2,34	FAIL
RHOG	P84095	ras homolog family member G	1,64	4,23	FAIL
RAC2	P15153	Rac family small GTPase 2	1,64	3,75	FAIL
YBX1	P67809	Y-box binding protein 1	1,64	1,82	FAIL
PSD4	Q8NDX1	pleckstrin and Sec7 domain containing 4	1,64	3,08	FAIL
ZCCHC8	Q6NZY4	zinc finger CCHC-type containing 8	1,64	1,94	FAIL
GLYR1	Q49A26	glyoxylate reductase 1 homolog	1,64	3,55	FAIL
ARHGAP4	P98171	Rho GTPase activating protein 4	1,64	2,56	FAIL
GOLGB1	Q14789	golgin B1	1,64	1,85	FAIL
ATP13A1	Q9HD20	ATPase 13A1	1,64	1,50	FAIL
RPLP2	P05387	ribosomal protein lateral stalk subunit P2	1,63	2,72	FAIL
PSMC3	P17980	proteasome 26S subunit, ATPase 3	1,63	3,91	FAIL
CTDNEP1	O95476	CTD nuclear envelope phosphatase 1	1,63	3,23	FAIL
UPF1	Q92900	UPF1 RNA helicase and ATPase	1,63	2,20	FAIL
VAPA	Q9P0L0	VAMP associated protein A	1,63	1,62	FAIL
IPO8	O15397	importin 8	1,63	2,18	FAIL
CCAR2	Q8N163	cell cycle and apoptosis regulator 2	1,63	3,71	FAIL
RXRB	P28702	retinoid X receptor beta	1,63	2,37	FAIL
FKBP8	Q14318	FKBP prolyl isomerase 8	1,63	1,86	FAIL
SRP68	Q9UHB9	signal recognition particle 68	1,63	4,05	FAIL
PEBP1	P30086	phosphatidylethanolamine binding protein 1	1,62	3,24	FAIL
C1orf174	Q8IYL3	chromosome 1 open reading frame 174	1,62	3,06	FAIL
YIPF5	Q969M3	Yip1 domain family member 5	1,62	2,42	FAIL
DPM3	Q9P2X0	dolichyl-phosphate mannosyltransferase subunit 3, regulatory	1,62	2,14	FAIL
PPID	Q08752	peptidylprolyl isomerase D	1,62	1,41	FAIL
IDH3B	O43837	isocitrate dehydrogenase (NAD(+)) 3 non-catalytic subunit beta	1,62	2,73	FAIL
NUP88	Q99567	nucleoporin 88	1,62	2,81	FAIL
CLTC	A0A087WVQ6	clathrin heavy chain	1,62	3,13	FAIL
	Q32MN6		1,62	1,56	FAIL
STT3A	P46977	STT3 oligosaccharyltransferase complex catalytic subunit A	1,62	3,80	FAIL
	H3BN98		1,62	2,14	FAIL
ARHGAP17	Q68EM7	Rho GTPase activating protein 17	1,62	2,43	FAIL
DNA2	P51530	DNA replication helicase/nuclease 2	1,62	2,57	FAIL
NUP210	Q8TEM1	nucleoporin 210	1,62	3,25	FAIL
ISY1-RAB43	Q9ULR0	ISY1-RAB43 readthrough	1,62	1,51	FAIL
TRABD	J3KPT4	TraB domain containing	1,62	2,04	FAIL
NCBP1	Q09161	nuclear cap binding protein subunit 1	1,62	4,07	FAIL
TRAM1	Q15629	translocation associated membrane protein 1	1,62	2,20	FAIL
MARS1	P56192	methionyl-tRNA synthetase 1	1,62	4,19	FAIL

KPNA2	P52292	karyopherin subunit alpha 2	1,62	2,22	FAIL
ZW10	O43264	zw10 kinetochore protein	1,61	1,40	FAIL
VTN	P04004	vitronectin	1,61	2,34	FAIL
TMX1	Q9H3N1	thioredoxin related transmembrane protein 1	1,61	2,94	FAIL
ERCC2	P18074	ERCC excision repair 2, TFIIH core complex helicase subunit	1,61	2,08	FAIL
USO1	O60763	USO1 vesicle transport factor	1,61	3,01	FAIL
SLC3A2	F5GZS6	solute carrier family 3 member 2	1,61	1,80	FAIL
ZAP70	P43403	zeta chain of T cell receptor associated protein kinase 70	1,61	3,95	FAIL
OXSR1	O95747	oxidative stress responsive kinase 1	1,61	1,62	FAIL
DDX24	Q9GZR7	DEAD-box helicase 24	1,61	1,75	FAIL
AP2M1	Q96CW1	adaptor related protein complex 2 subunit mu 1	1,61	2,40	FAIL
MTPAP	Q9NVV4	mitochondrial poly(A) polymerase	1,61	1,84	FAIL
ARHGAP11A	Q6P4F7	Rho GTPase activating protein 11A	1,61	1,36	FAIL
SEC62	F8WCJ7	SEC62 homolog, preprotein translocation factor	1,61	1,53	FAIL
ASCC3	Q8N3C0	activating signal cointegrator 1 complex subunit 3	1,61	2,68	FAIL
STK19	B7ZLI8	serine/threonine kinase 19	1,61	3,81	FAIL
CCZ1	P86790	CCZ1 homolog, vacuolar protein trafficking and biogenesis associated	1,61	2,20	FAIL
ASNS	P08243	asparagine synthetase (glutamine-hydrolyzing)	1,61	2,64	FAIL
THOC3	Q96J01	THO complex subunit 3	1,61	1,63	FAIL
RAB1A	P62820	RAB1A, member RAS oncogene family	1,61	2,50	FAIL
MBD2	Q9UBB5	methyl-CpG binding domain protein 2	1,61	1,95	FAIL
MPC1	A0A087WVZ0	mitochondrial pyruvate carrier 1	1,61	1,73	FAIL
UTP3	Q9NQZ2	UTP3 small subunit processome component	1,60	1,75	FAIL
LMF2	Q9BU23	lipase maturation factor 2	1,60	2,77	FAIL
	A0A1W2PRB8		1,60	3,14	FAIL
FIRRM	Q9NSG2	FIGNL1 interacting regulator of recombination and mitosis	1,60	2,08	FAIL
RPL24	P83731	ribosomal protein L24	1,60	1,54	FAIL
NUDT4	Q9NZJ9	nudix hydrolase 4	1,60	1,57	FAIL
S100B	A8MRB1	S100 calcium binding protein B	1,60	3,59	FAIL
DIP2B	Q9P265	disco interacting protein 2 homolog B	1,60	1,77	FAIL
P2RY8	Q86VZ1	P2Y receptor family member 8	1,60	2,94	FAIL
ACSL4	O60488	acyl-CoA synthetase long chain family member 4	1,60	2,55	FAIL
DCAF10	Q5QP82	DDB1 and CUL4 associated factor 10	1,60	2,37	FAIL
RAB5A	P20339	RAB5A, member RAS oncogene family	1,60	2,50	FAIL
CTDSPL2	Q05D32	CTD small phosphatase like 2	1,60	1,60	FAIL
MMS19	Q96T76	MMS19 homolog, cytosolic iron-sulfur assembly component	1,60	1,70	FAIL
LEMD2	Q8NC56	LEM domain nuclear envelope protein 2	1,60	1,92	FAIL
KCMF1	Q9P0J7	potassium channel modulatory factor 1	1,60	1,35	FAIL
SPCS3	P61009	signal peptidase complex subunit 3	1,60	3,80	FAIL
GPATCH8	Q9UKJ3	G-patch domain containing 8	1,60	2,76	FAIL
MAK16	Q9BXY0	MAK16 homolog	1,59	1,36	FAIL
RAB14	P61106	RAB14, member RAS oncogene family	1,59	1,63	FAIL

DHFR	P00374	dihydrofolate reductase	1,59	1,42	FAIL
TOP2A	P11388	DNA topoisomerase II alpha	1,59	2,44	FAIL
STX18	Q9P2W9	syntaxin 18	1,59	1,83	FAIL
SMG6	Q86US8	SMG6 nonsense mediated mRNA decay factor	1,59	2,37	FAIL
SHFL	Q9NUL5	shiftless antiviral inhibitor of ribosomal frameshifting	1,59	3,44	FAIL
DUS1L	H0YGW8	dihydrouridine synthase 1 like	1,59	1,83	FAIL
ACAT2	Q9BWD1	acetyl-CoA acetyltransferase 2	1,59	1,46	FAIL
UNC13D	Q70J99	unc-13 homolog D	1,59	1,84	FAIL
RPL18	G3V203	ribosomal protein L18	1,59	2,01	FAIL
SRP72	O76094	signal recognition particle 72	1,59	1,64	FAIL
AAMP	C9JG97	angio associated migratory cell protein	1,59	1,81	FAIL
SCYL1	Q96KG9	SCY1 like pseudokinase 1	1,59	2,09	FAIL
SEH1L	Q96EE3	SEH1 like nucleoporin	1,59	1,93	FAIL
EIF3B	P55884	eukaryotic translation initiation factor 3 subunit B	1,59	3,71	FAIL
TOMM20	Q15388	translocase of outer mitochondrial membrane 20	1,59	2,18	FAIL
VARS1	P26640	valyl-tRNA synthetase 1	1,59	2,82	FAIL
EI24	E9PM05	EI24 autophagy associated transmembrane protein	1,59	2,31	FAIL
DNM2	P50570	dynamin 2	1,59	4,39	FAIL
GFUS	Q13630	GDP-L-fucose synthase	1,59	3,02	FAIL
CARD11	Q9BXL7	caspase recruitment domain family member 11	1,58	2,32	FAIL
NAA35	Q5VZE5	N-alpha-acetyltransferase 35, NatC auxiliary subunit	1,58	1,41	FAIL
ZDHHC17	Q8IUH5	zinc finger DHHC-type palmitoyltransferase 17	1,58	3,59	FAIL
BYSL	Q13895	bystin like	1,58	3,35	FAIL
ABCC5	O15440	ATP binding cassette subfamily C member 5	1,58	1,78	FAIL
SQOR	Q9Y6N5	sulfide quinone oxidoreductase	1,58	1,64	FAIL
DPY19L1	A0A1B0GW05	dpy-19 like C-mannosyltransferase 1	1,58	2,47	FAIL
UQCRC1	P31930	ubiquinol-cytochrome c reductase core protein 1	1,58	2,92	FAIL
MAD2L1	Q13257	mitotic arrest deficient 2 like 1	1,58	2,84	FAIL
RING1	Q06587	ring finger protein 1	1,58	2,30	FAIL
RPL7A	P62424	ribosomal protein L7a	1,58	2,23	FAIL
NCAPD2	Q15021	non-SMC condensin I complex subunit D2	1,58	2,23	FAIL
SLC35F6	Q8N357	solute carrier family 35 member F6	1,58	1,41	FAIL
RBBP4	Q09028	RB binding protein 4, chromatin remodeling factor	1,58	1,71	FAIL
PTRH2	J3KQ48	peptidyl-tRNA hydrolase 2	1,58	1,58	FAIL
CKAP4	Q07065	cytoskeleton associated protein 4	1,58	2,23	FAIL
URB1	O60287	URB1 ribosome biogenesis homolog	1,58	2,97	FAIL
POLR1G	O15446	RNA polymerase I subunit G	1,58	1,69	FAIL
CSTF2T	Q9H0L4	cleavage stimulation factor subunit 2 tau variant	1,58	2,19	FAIL
SLC16A1	P53985	solute carrier family 16 member 1	1,58	3,08	FAIL
IGLL1	P15814	immunoglobulin lambda like polypeptide 1	1,58	1,87	FAIL
PUS1	G8JLB3	pseudouridine synthase 1	1,58	3,55	FAIL
PSMD13	Q9UNM6	proteasome 26S subunit, non-ATPase 13	1,58	1,58	FAIL
TRMU	B4DZN5	tRNA mitochondrial 2-thiouridylase	1,58	3,13	FAIL
HSPA1A	A0A0G2JIW1	heat shock protein family A (Hsp70) member 1A	1,58	3,06	FAIL
INTS6	Q9UL03	integrator complex subunit 6	1,57	1,73	FAIL
NUB1	Q9Y5A7	negative regulator of ubiquitin like proteins 1	1,57	2,00	FAIL
NEPRO	Q6NW34	nucleolus and neural progenitor protein	1,57	1,86	FAIL
HGH1	Q9BTY7	HGH1 homolog	1,57	1,97	FAIL

NUP107	P57740	nucleoporin 107	1,57	3,03	FAIL
COQ8A	Q8NI60	coenzyme Q8A	1,57	1,54	FAIL
HSD17B11	Q8NBQ5	hydroxysteroid 17-beta dehydrogenase 11	1,57	2,13	FAIL
CTBP2	P56545	C-terminal binding protein 2	1,57	2,72	FAIL
TBC1D10B	Q4KMP7	TBC1 domain family member 10B	1,57	3,15	FAIL
TIMM21	Q9BVV7	translocase of inner mitochondrial membrane 21	1,57	1,81	FAIL
	A0A0C4DGS9		1,57	2,44	FAIL
SYMPK	Q92797	symplekin scaffold protein	1,57	2,44	FAIL
COPE	O14579	COPI coat complex subunit epsilon	1,57	1,41	FAIL
ZNF512	Q96ME7	zinc finger protein 512	1,57	1,97	FAIL
SLC25A32	Q9H2D1	solute carrier family 25 member 32	1,56	1,40	FAIL
TIMELESS	Q9UNS1	timeless circadian regulator	1,56	3,65	FAIL
TSGA13	Q96PP4	testis specific 13	1,56	1,86	FAIL
TTF2	Q9UNY4	transcription termination factor 2	1,56	2,89	FAIL
MRPL16	Q9NX20	mitochondrial ribosomal protein L16	1,56	1,92	FAIL
NELFCD	X6RLT1	negative elongation factor complex member C/D	1,56	1,43	FAIL
ATAD3B	Q5T9A4	ATPase family AAA domain containing 3B	1,56	4,62	FAIL
SPTBN1	Q01082	spectrin beta, non-erythrocytic 1	1,56	2,63	FAIL
POLR2B	C9J2Y9	RNA polymerase II subunit B	1,55	2,66	FAIL
COX4I1	P13073	cytochrome c oxidase subunit 4I1	1,55	2,65	FAIL
MRPS22	P82650	mitochondrial ribosomal protein S22	1,55	3,21	FAIL
PHGDH	O43175	phosphoglycerate dehydrogenase	1,55	2,10	FAIL
DVL2	O14641	dishevelled segment polarity protein 2	1,55	1,68	FAIL
KIF22	Q14807	kinesin family member 22	1,55	3,96	FAIL
FRYL	O94915	FRY like transcription coactivator	1,55	2,57	FAIL
RHEB	Q15382	Ras homolog, mTORC1 binding	1,55	3,23	FAIL
PRDX4	H7C3T4	peroxiredoxin 4	1,55	2,09	FAIL
DNAJA3	Q96EY1	DnaJ heat shock protein family (Hsp40) member A3	1,55	1,65	FAIL
EMD	P50402	emerin	1,55	3,67	FAIL
DOCK10	Q96BY6	dedicator of cytokinesis 10	1,54	3,11	FAIL
SPC24	K7EMX1	SPC24 component of NDC80 kinetochore complex	1,54	1,72	FAIL
PHC2	A0A0A0MSI2	polyhomeotic homolog 2	1,54	2,15	FAIL
TUBB4B	P68371	tubulin beta 4B class IVb	1,54	1,93	FAIL
ABHD16A	A0A0G2JJD3	abhydrolase domain containing 16A, phospholipase	1,54	2,69	FAIL
MSI2	B4DHE8	musashi RNA binding protein 2	1,54	2,92	FAIL
RPRD1B	Q9NQG5	regulation of nuclear pre-mRNA domain containing 1B	1,54	1,96	FAIL
SNW1	Q13573	SNW domain containing 1	1,54	1,81	FAIL
TMA16	H0Y9X1	translation machinery associated 16 homolog	1,54	1,82	FAIL
ARHGEF2	V9GYF0	Rho/Rac guanine nucleotide exchange factor 2	1,54	2,60	FAIL
SEC61A1	B4DR61	SEC61 translocon subunit alpha 1	1,54	1,80	FAIL
RABL6	Q3YEC7	RAB, member RAS oncogene family like 6	1,54	1,69	FAIL
	H7C0V5		1,54	1,53	FAIL
FASTKD2	Q9NYY8	FAST kinase domains 2	1,54	3,54	FAIL
GNL2	Q13823	G protein nucleolar 2	1,54	3,42	FAIL
SLC25A1	P53007	solute carrier family 25 member 1	1,53	3,51	FAIL
	E7ENN3		1,53	1,64	FAIL
FKBP5	Q13451	FKBP prolyl isomerase 5	1,53	1,36	FAIL
RECQL	P46063	RecQ like helicase	1,53	2,91	FAIL

POMP	Q9Y244	proteasome maturation protein	1,53	1,31	FAIL
CTR9	Q6PD62	CTR9 homolog, Paf1/RNA polymerase II complex component	1,53	2,14	FAIL
TOE1	Q96GM8	target of EGR1, exonuclease	1,53	2,43	FAIL
ANKHD1	Q8IWZ3	ankyrin repeat and KH domain containing 1	1,53	3,69	FAIL
RPS4Y1	P22090	ribosomal protein S4 Y-linked 1	1,53	2,90	FAIL
CHD4	A0A2U3TZM0	chromodomain helicase DNA binding protein 4	1,53	2,26	FAIL
SHPK	A0A0B4J2A0	sedoheptulokinase	1,53	1,34	FAIL
CCT6A	P40227	chaperonin containing TCP1 subunit 6A	1,53	1,70	FAIL
TRAPPC1	I3L3S0	trafficking protein particle complex subunit 1	1,53	1,79	FAIL
GTF2H5	Q6ZYL4	general transcription factor IIH subunit 5	1,53	2,89	FAIL
HSD17B10	Q99714	hydroxysteroid 17-beta dehydrogenase 10	1,53	2,18	FAIL
RAD50	Q92878	RAD50 double strand break repair protein	1,53	1,53	FAIL
HSD17B12	Q53GQ0	hydroxysteroid 17-beta dehydrogenase 12	1,53	2,70	FAIL
NARF	Q9UHQ1	nuclear prelamin A recognition factor	1,53	2,09	FAIL
SLIRP	G3V2S9	SRA stem-loop interacting RNA binding protein	1,53	1,42	FAIL
PTK2B	Q14289	protein tyrosine kinase 2 beta	1,53	2,54	FAIL
SEC24B	O95487	SEC24 homolog B, COPII coat complex component	1,53	2,90	FAIL
RABL2B	Q9UNT1	RAB, member of RAS oncogene family like 2B	1,53	1,50	FAIL
MCM2	P49736	minichromosome maintenance complex component 2	1,53	1,57	FAIL
DOCK2	Q92608	dedicator of cytokinesis 2	1,53	1,87	FAIL
BAZ2B	Q9UIF8	bromodomain adjacent to zinc finger domain 2B	1,52	1,83	FAIL
	F5H5I6		1,52	1,79	FAIL
SLX9	Q9NSI2	SLX9 ribosome biogenesis factor	1,52	1,72	FAIL
SERINC5	A0A0J9YYI4	serine incorporator 5	1,52	1,75	FAIL
CDK2	P24941	cyclin dependent kinase 2	1,52	2,68	FAIL
TESC	Q96BS2	tescalcin	1,52	2,75	FAIL
INTS11	Q5TA45	integrator complex subunit 11	1,52	1,36	FAIL
TTC17	Q96AE7	tetratricopeptide repeat domain 17	1,52	1,44	FAIL
MYB	P10242	MYB proto-oncogene, transcription factor	1,52	2,86	FAIL
MTHFD1L	B7ZM99	methylenetetrahydrofolate dehydrogenase (NADP+ dependent) 1 like	1,52	2,39	FAIL
YLPM1	P49750	YLP motif containing 1	1,52	3,39	FAIL
SMARCA5	O60264	SWI/SNF related, matrix associated, actin dependent regulator of chromatin, subfamily a, member 5	1,52	2,98	FAIL
TAOK3	Q9H2K8	TAO kinase 3	1,52	2,54	FAIL
ARL2	P36404	ADP ribosylation factor like GTPase 2	1,51	2,67	FAIL
CDK2AP1	O14519	cyclin dependent kinase 2 associated protein 1	1,51	1,92	FAIL
ORC5	O43913	origin recognition complex subunit 5	1,51	1,35	FAIL
TRIM33	Q9UPN9	tripartite motif containing 33	1,51	3,02	FAIL
CWC15	Q9P013	CWC15 spliceosome associated protein homolog	1,51	1,73	FAIL
MCM9	Q9NXL9	minichromosome maintenance 9 homologous recombination repair factor	1,51	2,58	FAIL
RBM7	J3KPD3	RNA binding motif protein 7	1,51	1,79	FAIL
SYNGR2	K7ELS8	synaptogyrin 2	1,51	2,27	FAIL

ARPC4	P59998	actin related protein 2/3 complex subunit 4	1,51	2,01	FAIL
CHERP	Q8IWX8	calcium homeostasis endoplasmic reticulum protein	1,51	1,64	FAIL
MAP2K3	P46734	mitogen-activated protein kinase kinase 3	1,51	2,37	FAIL
RAP1GAP	F2Z357	RAP1 GTPase activating protein	1,51	2,51	FAIL
GRWD1	Q9BQ67	glutamate rich WD repeat containing 1	1,51	2,47	FAIL
CHD1	O14646	chromodomain helicase DNA binding protein 1	1,51	3,70	FAIL
CWC22	Q9HCG8	CWC22 spliceosome associated protein homolog	1,50	2,33	FAIL
ARF3	P61204	ADP ribosylation factor 3	1,50	1,93	FAIL
KAT8	Q9H7Z6	lysine acetyltransferase 8	1,50	2,61	FAIL
TEX264	A0A087WTU3	testis expressed 264, ER-phagy receptor	1,50	2,61	FAIL
MRPS34	C9JJ19	mitochondrial ribosomal protein S34	1,50	2,45	FAIL
SBNO1	A3KN83	strawberry notch homolog 1	1,50	1,52	FAIL
VPS52	Q8N1B4	VPS52 subunit of GARP complex	1,50	1,94	FAIL
PSMC5	P62195	proteasome 26S subunit, ATPase 5	1,50	1,85	FAIL
DCAKD	Q8WVC6	dephospho-CoA kinase domain containing	1,50	2,98	FAIL
DEF6	Q9H4E7	DEF6 guanine nucleotide exchange factor	1,50	2,85	FAIL
WDR37	Q9Y2I8	WD repeat domain 37	1,50	2,16	FAIL
FOXK2	Q01167	forkhead box K2	1,50	2,39	FAIL
ARL8A	Q96BM9	ADP ribosylation factor like GTPase 8A	1,50	2,13	FAIL
MBOAT7	Q96N66	membrane bound O-acyltransferase domain containing 7	1,49	3,91	FAIL
SNRPB	P14678	small nuclear ribonucleoprotein polypeptides B and B1	1,49	1,75	FAIL
BCAS2	O75934	BCAS2 pre-mRNA processing factor	1,49	2,47	FAIL
VPS35L	E7EWW0	VPS35 endosomal protein sorting factor like	1,49	1,55	FAIL
ARCN1	P48444	archain 1	1,49	1,47	FAIL
RHBDD2	Q6NTF9	rhomboid domain containing 2	1,49	1,98	FAIL
DYM	Q7RTS9	dymeclin	1,49	3,12	FAIL
NUP93	Q8N1F7	nucleoporin 93	1,49	3,06	FAIL
GOLPH3	Q9H4A6	golgi phosphoprotein 3	1,49	2,32	FAIL
POLR1B	Q9H9Y6	RNA polymerase I subunit B	1,49	3,79	FAIL
SLC35A1	P78382	solute carrier family 35 member A1	1,49	1,42	FAIL
TMPO	P42167	thymopoietin	1,49	2,04	FAIL
CCT5	P48643	chaperonin containing TCP1 subunit 5	1,49	1,62	FAIL
KDM3A	Q9Y4C1	lysine demethylase 3A	1,49	2,00	FAIL
FHL2	Q14192	four and a half LIM domains 2	1,49	2,31	FAIL
NUP62	P37198	nucleoporin 62	1,49	1,73	FAIL
CRTC2	Q53ET0	CREB regulated transcription coactivator 2	1,49	2,13	FAIL
SAC3D1	F8WC89	SAC3 domain containing 1	1,49	2,78	FAIL
PTP4A2	Q12974	protein tyrosine phosphatase 4A2	1,49	1,49	FAIL
CTPS1	P17812	CTP synthase 1	1,49	4,25	FAIL
RASSF2	P50749	Ras association domain family member 2	1,49	1,41	FAIL
CDS2	O95674	CDP-diacylglycerol synthase 2	1,49	5,00	FAIL
PDCD11	Q14690	programmed cell death 11	1,49	2,85	FAIL
GPD1L	Q8N335	glycerol-3-phosphate dehydrogenase 1 like	1,49	2,82	FAIL
RPL8	P62917	ribosomal protein L8	1,48	2,82	FAIL
RAB11B	Q15907	RAB11B, member RAS oncogene family	1,48	1,49	FAIL
SAMD1	Q6SPF0	sterile alpha motif domain containing 1	1,48	2,17	FAIL
SNU13	A0A3B3IUA2	small nuclear ribonucleoprotein 13	1,48	1,76	FAIL

PCID2	Q5JVF3	PCI domain containing 2	1,48	3,38	FAIL
GNL3	Q9BVP2	G protein nucleolar 3	1,48	1,50	FAIL
TERF2	Q15554	telomeric repeat binding factor 2	1,48	1,60	FAIL
NOC4L	Q9BVI4	nucleolar complex associated 4 homolog	1,48	1,59	FAIL
SMYD2	Q9NRG4	SET and MYND domain containing 2	1,48	2,15	FAIL
STAT3	P40763	signal transducer and activator of transcription 3	1,48	4,43	FAIL
DDX3Y	O15523	DEAD-box helicase 3 Y-linked	1,48	1,99	FAIL
NDUFA4	O00483	NDUFA4 mitochondrial complex associated	1,48	2,89	FAIL
PAN2	Q504Q3	poly(A) specific ribonuclease subunit PAN2	1,48	1,49	FAIL
GOLGA7	Q7Z5G4	golgin A7	1,48	1,90	FAIL
CLN6	H3BUV4	CLN6 transmembrane ER protein	1,47	1,74	FAIL
VPS4B	O75351	vacuolar protein sorting 4 homolog B	1,47	3,89	FAIL
ARF4	P18085	ADP ribosylation factor 4	1,47	2,56	FAIL
PDS5A	Q29RF7	PDS5 cohesin associated factor A	1,47	3,01	FAIL
REEP4	Q9H6H4	receptor accessory protein 4	1,47	1,81	FAIL
	A0A3B3ITX4		1,47	2,65	FAIL
MRPL11	Q9Y3B7	mitochondrial ribosomal protein L11	1,47	1,79	FAIL
PHF20	Q5JWZ0	PHD finger protein 20	1,47	1,60	FAIL
GAS2L1	A0A5E8	growth arrest specific 2 like 1	1,47	3,00	FAIL
JPH1	Q9HDC5	junctophilin 1	1,47	2,55	FAIL
BLM	P54132	BLM RecQ like helicase	1,47	2,44	FAIL
COMT	P21964	catechol-O-methyltransferase	1,47	3,05	FAIL
CDC5L	Q99459	cell division cycle 5 like	1,47	2,54	FAIL
FERMT3	Q86UX7	FERM domain containing kindlin 3	1,47	3,08	FAIL
HAUS6	Q7Z4H7	HAUS augmin like complex subunit 6	1,47	2,03	FAIL
EPRS1	P07814	glutamyl-prolyl-tRNA synthetase 1	1,46	2,35	FAIL
JUND	P17535	JunD proto-oncogene, AP-1 transcription factor subunit	1,46	1,57	FAIL
ABCB10	Q9NRK6	ATP binding cassette subfamily B member 10	1,46	3,08	FAIL
RAB10	P61026	RAB10, member RAS oncogene family	1,46	1,76	FAIL
MAPRE2	Q15555	microtubule associated protein RP/EB family member 2	1,46	3,56	FAIL
NSG1	P42857	neuronal vesicle trafficking associated 1	1,46	1,43	FAIL
FCGBP	Q9Y6R7	Fc gamma binding protein	1,46	2,56	FAIL
	A0A0A0MSU2		1,46	1,67	FAIL
GTF2E2	P29084	general transcription factor IIE subunit 2	1,46	3,71	FAIL
RNF2	Q99496	ring finger protein 2	1,46	1,49	FAIL
RAB33A	Q14088	RAB33A, member RAS oncogene family	1,46	2,56	FAIL
RPS20	P60866	ribosomal protein S20	1,46	1,52	FAIL
	A0A669KAZ0		1,46	1,87	FAIL
TIMP1	Q5H9B4	TIMP metallopeptidase inhibitor 1	1,46	3,36	FAIL
MAGI1	Q96QZ7	membrane associated guanylate kinase, WW and PDZ domain containing 1	1,46	1,97	FAIL
NSUN5	Q96P11	NOP2/Sun RNA methyltransferase 5	1,46	2,09	FAIL
CHD2	O14647	chromodomain helicase DNA binding protein 2	1,46	1,95	FAIL
CXCR4	P61073	C-X-C motif chemokine receptor 4	1,45	2,36	FAIL
TELO2	Q9Y4R8	telomere maintenance 2	1,45	2,77	FAIL
IK	Q13123	IK cytokine	1,45	1,60	FAIL
TFRC	P02786	transferrin receptor	1,45	2,16	FAIL

SDR39U1	Q9NRG7	short chain dehydrogenase/reductase family 39U member 1	1,45	2,02	FAIL
IPO9	Q96P70	importin 9	1,45	2,59	FAIL
LRRC59	Q96AG4	leucine rich repeat containing 59	1,45	3,19	FAIL
SNX6	Q9UNH7	sorting nexin 6	1,45	2,41	FAIL
NDUFA9	Q16795	NADH:ubiquinone oxidoreductase subunit A9	1,45	2,75	FAIL
ERLIN1	O75477	ER lipid raft associated 1	1,45	2,04	FAIL
CDT1	Q9H211	chromatin licensing and DNA replication factor 1	1,45	4,29	FAIL
KIF11	P52732	kinesin family member 11	1,45	3,42	FAIL
PSMD8	P48556	proteasome 26S subunit, non-ATPase 8	1,45	3,16	FAIL
TENM2	Q9NT68	teneurin transmembrane protein 2	1,45	1,78	FAIL
TUBG1	P23258	tubulin gamma 1	1,45	1,86	FAIL
RPS28	P62857	ribosomal protein S28	1,45	1,87	FAIL
WDR3	Q9UNX4	WD repeat domain 3	1,45	3,90	FAIL
RARS2	Q5T160	arginyl-tRNA synthetase 2, mitochondrial	1,45	1,56	FAIL
SARS1	Q5T5C7	seryl-tRNA synthetase 1	1,45	2,30	FAIL
EHD1	A0A024R571	EH domain containing 1	1,45	2,24	FAIL
FXR2	P51116	FMR1 autosomal homolog 2	1,45	3,36	FAIL
TOR1AIP2	Q8NFQ8	torsin 1A interacting protein 2	1,45	1,45	FAIL
ERGIC1	Q969X5	endoplasmic reticulum-golgi intermediate compartment 1	1,45	2,71	FAIL
THNSL1	Q8IYQ7	threonine synthase like 1	1,45	1,85	FAIL
ALDH5A1	P51649	aldehyde dehydrogenase 5 family member A1	1,45	1,55	FAIL
ARNT	P27540	aryl hydrocarbon receptor nuclear translocator	1,44	2,01	FAIL
HADHB	P55084	hydroxyacyl-CoA dehydrogenase trifunctional multienzyme complex subunit beta	1,44	3,60	FAIL
MDH1	B8ZZ51	malate dehydrogenase 1	1,44	1,34	FAIL
PRDX2	P32119	peroxiredoxin 2	1,44	2,43	FAIL
SLC25A3	Q00325	solute carrier family 25 member 3	1,44	2,14	FAIL
DNM1L	O00429	dynamin 1 like	1,44	1,58	FAIL
RPS3	P23396	ribosomal protein S3	1,44	2,56	FAIL
LENG8	A0A087WUE4	leukocyte receptor cluster member 8	1,44	1,63	FAIL
MRPS27	G5EA06	mitochondrial ribosomal protein S27	1,44	2,27	FAIL
HNRNPF	P52597	heterogeneous nuclear ribonucleoprotein F	1,44	3,43	FAIL
CYBA	P13498	cytochrome b-245 alpha chain	1,44	1,97	FAIL
ORMDL2	F8VXD5	ORMDL sphingolipid biosynthesis regulator 2	1,44	2,25	FAIL
TARS2	Q9BW92	threonyl-tRNA synthetase 2, mitochondrial	1,44	2,43	FAIL
HAUS1	Q96CS2	HAUS augmin like complex subunit 1	1,44	1,50	FAIL
STOML2	Q9UJZ1	stomatin like 2	1,44	2,37	FAIL
NOP56	O00567	NOP56 ribonucleoprotein	1,43	1,81	FAIL
DHCR24	Q15392	24-dehydrocholesterol reductase	1,43	3,23	FAIL
CPSF2	Q9P2I0	cleavage and polyadenylation specific factor 2	1,43	3,57	FAIL
MCM4	P33991	minichromosome maintenance complex component 4	1,43	4,11	FAIL
CHTF18	Q8WVB6	chromosome transmission fidelity factor 18	1,43	2,36	FAIL
FAM120A	Q9NZB2	family with sequence similarity 120 member A	1,43	2,56	FAIL
RPS27L	Q71UM5	ribosomal protein S27 like	1,43	1,92	FAIL
ACOT7	O00154	acyl-CoA thioesterase 7	1,43	1,33	FAIL

	Q5TZK3		1,43	3,27	FAIL
HNRNPR	O43390	heterogeneous nuclear ribonucleoprotein R	1,43	2,18	FAIL
AKAP8	O43823	A-kinase anchoring protein 8	1,43	1,41	FAIL
COPZ1	P61923	COPI coat complex subunit zeta 1	1,43	2,67	FAIL
TMEM68	Q96MH6	transmembrane protein 68	1,43	2,18	FAIL
COPG1	Q9Y678	COPI coat complex subunit gamma 1	1,43	2,29	FAIL
SMARCA4	P51532	SWI/SNF related, matrix associated, actin dependent regulator of chromatin, subfamily a, member 4	1,43	2,75	FAIL
DNAAF5	Q86Y56	dynein axonemal assembly factor 5	1,42	2,17	FAIL
EXOC4	Q96A65	exocyst complex component 4	1,42	2,21	FAIL
EXOC3	O60645	exocyst complex component 3	1,42	1,39	FAIL
KATNA1	O75449	katanin catalytic subunit A1	1,42	3,06	FAIL
PTBP1	A6NLN1	polypyrimidine tract binding protein 1	1,42	3,14	FAIL
	F6Y5H0		1,42	3,19	FAIL
RFTN1	Q14699	raftlin, lipid raft linker 1	1,42	1,69	FAIL
RPS27	P42677	ribosomal protein S27	1,42	1,88	FAIL
RRAGC	Q9HB90	Ras related GTP binding C	1,42	2,24	FAIL
RAB6A	P20340	RAB6A, member RAS oncogene family	1,42	2,58	FAIL
ADAR	P55265	adenosine deaminase RNA specific	1,42	3,14	FAIL
NOM1	Q5C9Z4	nucleolar protein with MIF4G domain 1	1,41	1,90	FAIL
SLC38A9	D6R9X0	solute carrier family 38 member 9	1,41	3,23	FAIL
SLC25A6	P12236	solute carrier family 25 member 6	1,41	1,65	FAIL
RTN1	A8MT72	reticulon 1	1,41	2,33	FAIL
TMEM41B	Q5BJD5	transmembrane protein 41B	1,41	1,69	FAIL
PLEKHA7	E9PKC0	pleckstrin homology domain containing A7	1,41	1,39	FAIL
BOP1	Q14137	BOP1 ribosomal biogenesis factor	1,41	2,00	FAIL
SLC25A4	P12235	solute carrier family 25 member 4	1,41	2,37	FAIL
XRCC6	P12956	X-ray repair cross complementing 6	1,41	1,72	FAIL
LSM6	P62312	LSM6 homolog, U6 small nuclear RNA and mRNA degradation associated	1,41	3,55	FAIL
FANCI	Q9NVI1	FA complementation group I	1,41	1,83	FAIL
ORAI1	Q96D31	ORAI calcium release-activated calcium modulator 1	1,40	2,07	FAIL
UBE2M	P61081	ubiquitin conjugating enzyme E2 M	1,40	1,94	FAIL
DTX1	Q86Y01	deltex E3 ubiquitin ligase 1	1,40	1,84	FAIL
GLUL	A0A2R8YDT1	glutamate-ammonia ligase	1,40	1,39	FAIL
	F8W6I7		1,40	1,45	FAIL
PSMB10	P40306	proteasome 20S subunit beta 10	1,40	1,42	FAIL
STXBP4	Q6ZWJ1	syntaxin binding protein 4	1,39	2,46	FAIL
CAMK2D	Q13557	calcium/calmodulin dependent protein kinase II delta	1,39	2,64	FAIL
MDN1	Q9NU22	midasin AAA ATPase 1	1,39	2,48	FAIL
MRPL24	Q96A35	mitochondrial ribosomal protein L24	1,39	1,60	FAIL
MBD1	Q9UIS9	methyl-CpG binding domain protein 1	1,39	1,65	FAIL
UROS	A0A087X021	uroporphyrinogen III synthase	1,39	3,88	FAIL
LRRC36	Q1X8D7	leucine rich repeat containing 36	1,39	3,88	FAIL
ATP5ME	P56385	ATP synthase membrane subunit e	1,39	2,52	FAIL
RFC1	P35251	replication factor C subunit 1	1,39	2,15	FAIL

FOCAD	Q5VW36	focadhesin	1,39	3,71	FAIL
HSPA5	P11021	heat shock protein family A (Hsp70) member 5	1,39	2,66	FAIL
RAF1	P04049	Raf-1 proto-oncogene, serine/threonine kinase	1,39	1,73	FAIL
JAGN1	Q8N5M9	jagunal homolog 1	1,39	1,77	FAIL
TKFC	Q3LXA3	triokinase and FMN cyclase	1,39	1,61	FAIL
KDM5C	P41229	lysine demethylase 5C	1,39	1,88	FAIL
TP53I11	E9PIN5	tumor protein p53 inducible protein 11	1,39	2,19	FAIL
MYO9B	M0R0P8	myosin IXB	1,38	3,16	FAIL
DDX42	Q86XP3	DEAD-box helicase 42	1,38	3,34	FAIL
ATP5PD	O75947	ATP synthase peripheral stalk subunit d	1,38	3,54	FAIL
PPAN-P2RY11	A0A0B4J1V8	PPAN-P2RY11 readthrough	1,38	2,88	FAIL
RPS5	M0R0F0	ribosomal protein S5	1,38	3,63	FAIL
CENPB	P07199	centromere protein B	1,38	2,93	FAIL
AFG3L2	Q9Y4W6	AFG3 like matrix AAA peptidase subunit 2	1,38	1,93	FAIL
TCOF1	E7ETY2	treacle ribosome biogenesis factor 1	1,38	3,07	FAIL
TONSL	Q96HA7	tonsoku like, DNA repair protein	1,38	2,13	FAIL
MTHFD1	P11586	methylenetetrahydrofolate dehydrogenase, cyclohydro-lase and formyltetrahydrofolate synthetase 1	1,38	2,90	FAIL
POLD3	Q15054	DNA polymerase delta 3, accessory subunit	1,38	3,26	FAIL
ABCF2	Q9UG63	ATP binding cassette subfamily F member 2	1,38	1,73	FAIL
OGT	O15294	O-linked N-acetylglucosamine (GlcNAc) transferase	1,38	1,43	FAIL
GMPPA	Q96IJ6	GDP-mannose pyrophosphorylase A	1,38	1,87	FAIL
EIF3F	O00303	eukaryotic translation initiation factor 3 subunit F	1,38	2,52	FAIL
TDRD3	Q9H7E2	tudor domain containing 3	1,38	1,59	FAIL
PWP1	B4DJV5	PWP1 homolog, endonuclein	1,38	2,02	FAIL
CPSF4	O95639	cleavage and polyadenylation specific factor 4	1,38	2,01	FAIL
NPM1	P06748	nucleophosmin 1	1,38	1,80	FAIL
SF3B1	O75533	splicing factor 3b subunit 1	1,38	2,43	FAIL
TCERG1	O14776	transcription elongation regulator 1	1,38	2,74	FAIL
NFRKB	Q6P4R8	nuclear factor related to kappaB binding protein	1,38	1,70	FAIL
TBRG4	Q969Z0	transforming growth factor beta regulator 4	1,37	3,11	FAIL
CHUK	O15111	component of inhibitor of nuclear factor kappa B kinase complex	1,37	1,53	FAIL
SLC25A22	E9PJH7	solute carrier family 25 member 22	1,37	2,93	FAIL
XPO7	E7ESC6	exportin 7	1,37	2,75	FAIL
TEX30	A0A0C4DFW4	testis expressed 30	1,37	1,38	FAIL
NMD3	Q96D46	NMD3 ribosome export adaptor	1,37	1,33	FAIL
EIF2B4	E7ERK9	eukaryotic translation initiation factor 2B subunit delta	1,37	2,47	FAIL
RPL17	J3KRX5	ribosomal protein L17	1,37	1,62	FAIL
GPBP1L1	Q9HC44	GC-rich promoter binding protein 1 like 1	1,37	2,12	FAIL
MAP3K6	O95382	mitogen-activated protein kinase kinase kinase 6	1,37	1,30	FAIL
RPLP0	P05388	ribosomal protein lateral stalk subunit P0	1,37	1,98	FAIL
PNO1	Q9NRX1	partner of NOB1 homolog	1,37	1,99	FAIL
TBC1D31	E5RI94	TBC1 domain family member 31	1,37	2,39	FAIL
HEATR1	Q9H583	HEAT repeat containing 1	1,37	1,57	FAIL
LEO1	Q8WVC0	LEO1 homolog, Paf1/RNA polymerase II complex com-ponent	1,37	2,68	FAIL

DNAJB6	A0A0J9YX62	DnaJ heat shock protein family (Hsp40) member B6	1,36	2,37	FAIL
ARPC2	O15144	actin related protein 2/3 complex subunit 2	1,36	1,52	FAIL
POLR1E	Q9GZS1	RNA polymerase I subunit E	1,36	3,84	FAIL
SVIL	A0A6I8PIX7	supervillin	1,36	2,78	FAIL
SMARCAD1	Q9H4L7	SWI/SNF-related, matrix-associated actin-dependent regulator of chromatin, subfamily a, containing DEAD/H box 1	1,36	2,56	FAIL
POLRMT	O00411	RNA polymerase mitochondrial	1,36	1,82	FAIL
TRMT1L	Q7Z2T5	tRNA methyltransferase 1 like	1,36	1,51	FAIL
	A0A024RAC6		1,36	1,84	FAIL
COPG2	Q9UBF2	COPI coat complex subunit gamma 2	1,36	3,17	FAIL
AP2B1	A0A087X253	adaptor related protein complex 2 subunit beta 1	1,36	2,99	FAIL
LEMD3	Q9Y2U8	LEM domain containing 3	1,36	1,97	FAIL
CDC27	G5EA36	cell division cycle 27	1,36	4,36	FAIL
EP400	Q96L91	E1A binding protein p400	1,36	2,25	FAIL
CSTF3	Q12996	cleavage stimulation factor subunit 3	1,36	1,82	FAIL
BLOC1S5	Q8TDH9	biogenesis of lysosomal organelles complex 1 subunit 5	1,36	1,97	FAIL
CBX3	Q13185	chromobox 3	1,36	1,63	FAIL
MAP1A	E9PGC8	microtubule associated protein 1A	1,35	2,04	FAIL
RCC1	P18754	regulator of chromosome condensation 1	1,35	2,69	FAIL
TECR	Q9NZ01	trans-2,3-enoyl-CoA reductase	1,35	1,79	FAIL
HK2	E9PB90	hexokinase 2	1,35	1,30	FAIL
SIX6	O95475	SIX homeobox 6	1,35	3,16	FAIL
PFKP	Q01813	phosphofructokinase, platelet	1,35	1,74	FAIL
NIPSNAP2	O75323	nipsnap homolog 2	1,35	3,23	FAIL
FUBP3	Q96I24	far upstream element binding protein 3	1,35	3,24	FAIL
PSIP1	O75475	PC4 and SRSF1 interacting protein 1	1,35	2,28	FAIL
C12orf43	F5H7W8	chromosome 12 open reading frame 43	1,35	1,56	FAIL
DDX21	Q9NR30	DExD-box helicase 21	1,35	2,00	FAIL
MCU	Q8NE86	mitochondrial calcium uniporter	1,35	2,11	FAIL
IARS1	P41252	isoleucyl-tRNA synthetase 1	1,35	1,88	FAIL
RFC2	P35250	replication factor C subunit 2	1,35	3,92	FAIL
REPS1	Q96D71	RALBP1 associated Eps domain containing 1	1,35	1,44	FAIL
PNKD	Q8N490	PNKD metallo-beta-lactamase domain containing	1,35	1,39	FAIL
HADHA	H0YFD6	hydroxyacyl-CoA dehydrogenase trifunctional multienzyme complex subunit alpha	1,35	2,50	FAIL
MYH9	P35579	myosin heavy chain 9	1,35	1,96	FAIL
DIS3	Q9Y2L1	DIS3 homolog, exosome endoribonuclease and 3'-5' exoribonuclease	1,35	1,32	FAIL
NTPCR	Q5TDF0	nucleoside-triphosphatase, cancer-related	1,35	3,20	FAIL
CLPP	Q16740	caseinolytic mitochondrial matrix peptidase proteolytic subunit	1,35	2,68	FAIL
MUL1	Q969V5	mitochondrial E3 ubiquitin protein ligase 1	1,34	1,77	FAIL

RPS8	P62241	ribosomal protein S8	1,34	2,68	FAIL
FADS1	A0A0A0MR51	fatty acid desaturase 1	1,34	3,22	FAIL
NAT10	Q9H0A0	N-acetyltransferase 10	1,34	2,29	FAIL
NOP58	Q9Y2X3	NOP58 ribonucleoprotein	1,34	2,15	FAIL
	H0YDQ1		1,34	2,77	FAIL
MRPS5	P82675	mitochondrial ribosomal protein S5	1,34	2,46	FAIL
VWF	P04275	von Willebrand factor	1,34	1,61	FAIL
TOX4	O94842	TOX high mobility group box family member 4	1,34	2,23	FAIL
UNK	Q9C0B0	unk zinc finger	1,34	2,80	FAIL
TTC27	Q6P3X3	tetratricopeptide repeat domain 27	1,34	1,35	FAIL
PRORP	O15091	protein only RNase P catalytic subunit	1,34	1,84	FAIL
POU6F2	P78424	POU class 6 homeobox 2	1,34	2,36	FAIL
LPCAT3	Q6P1A2	lysophosphatidylcholine acyltransferase 3	1,34	3,13	FAIL
TOMM7	Q9P0U1	translocase of outer mitochondrial membrane 7	1,34	2,28	FAIL
DOCK5	Q9H7D0	dedicator of cytokinesis 5	1,34	2,51	FAIL
CDC45	O75419	cell division cycle 45	1,34	2,11	FAIL
NSA2	A0A0A0MQZ6	NSA2 ribosome biogenesis factor	1,34	2,49	FAIL
VPS35	Q96QK1	VPS35 retromer complex component	1,34	1,78	FAIL
	Q5VTU8		1,34	2,57	FAIL
TUBGCP4	Q9UGJ1	tubulin gamma complex component 4	1,33	1,34	FAIL
GLIPR2	Q9H4G4	GLI pathogenesis related 2	1,33	2,37	FAIL
TCF25	Q9BQ70	transcription factor 25	1,33	2,39	FAIL
NET1	Q7Z628	neuroepithelial cell transforming 1	1,33	1,38	FAIL
ZBTB40	Q9NUA8	zinc finger and BTB domain containing 40	1,33	1,68	FAIL
MAZ	H3BQI4	MYC associated zinc finger protein	1,33	1,39	FAIL
RPL30	P62888	ribosomal protein L30	1,33	1,69	FAIL
FAM120C	Q9NX05	family with sequence similarity 120 member C	1,33	2,73	FAIL
EZH2	Q15910	enhancer of zeste 2 polycomb repressive complex 2 sub-unit	1,33	3,01	FAIL
ABHD17B	Q5VST6	abhydrolase domain containing 17B, depalmitoylase	1,33	2,52	FAIL
ATP5F1C	P36542	ATP synthase F1 subunit gamma	1,32	1,99	FAIL
ITPKB	P27987	inositol-trisphosphate 3-kinase B	1,32	2,37	FAIL
GEMIN2	G5EA29	gem nuclear organelle associated protein 2	1,32	3,03	FAIL
UCHL3	P15374	ubiquitin C-terminal hydrolase L3	1,32	1,48	FAIL
	A0A087WYN9		1,32	2,14	FAIL
SDAD1	Q9NVU7	SDA1 domain containing 1	1,32	3,39	FAIL
VPS26A	O75436	VPS26 retromer complex component A	1,32	1,45	FAIL
POM121	Q96HA1	POM121 transmembrane nucleoporin	1,32	1,38	FAIL
NUP85	Q9BW27	nucleoporin 85	1,32	2,47	FAIL
ACAD9	Q9H845	acyl-CoA dehydrogenase family member 9	1,32	1,45	FAIL
MAP2K7	O14733	mitogen-activated protein kinase kinase 7	1,32	2,07	FAIL
TRIM28	Q13263	tripartite motif containing 28	1,32	1,44	FAIL
LARP4B	Q92615	La ribonucleoprotein 4B	1,32	1,69	FAIL
ACAD8	Q9UKU7	acyl-CoA dehydrogenase family member 8	1,32	2,94	FAIL
DIMT1	Q9UNQ2	DIM1 rRNA methyltransferase and ribosome matura-tion factor	1,32	2,27	FAIL

SERBP1	Q8NC51	SERPINE1 mRNA binding protein 1	1,32	1,84	FAIL
RIOK1	Q9BRS2	RIO kinase 1	1,32	1,74	FAIL
RAB24	F8W8H5	RAB24, member RAS oncogene family	1,32	1,38	FAIL
DPM1	H0Y368	dolichyl-phosphate mannosyltransferase subunit 1, catalytic	1,32	3,12	FAIL
RPP30	Q5VU11	ribonuclease P/MRP subunit p30	1,31	1,30	FAIL
TERF1	P54274	telomeric repeat binding factor 1	1,31	1,82	FAIL
RPS23	P62266	ribosomal protein S23	1,31	1,47	FAIL
SLC25A5	P05141	solute carrier family 25 member 5	1,31	2,89	FAIL
DPY30	Q9C005	dpy-30 histone methyltransferase complex regulatory subunit	1,31	2,03	FAIL
MRTO4	Q9UKD2	MRT4 homolog, ribosome maturation factor	1,31	2,96	FAIL
LAP3	P28838	leucine aminopeptidase 3	1,31	2,75	FAIL
AEBP2	Q6ZN18	AE binding protein 2	1,31	2,87	FAIL
ESCO2	Q56NI9	establishment of sister chromatid cohesion N-acetyltransferase 2	1,31	1,37	FAIL
SLC39A7	A2AAT0	solute carrier family 39 member 7	1,31	1,32	FAIL
MBD3	O95983	methyl-CpG binding domain protein 3	1,31	3,50	FAIL
CFAP20	Q9Y6A4	cilia and flagella associated protein 20	1,30	1,61	FAIL
TENT2	Q6PIY7	terminal nucleotidyltransferase 2	1,30	1,87	FAIL
DHX9	Q08211	DExH-box helicase 9	1,30	2,13	FAIL
EMG1	Q92979	EMG1 N1-specific pseudouridine methyltransferase	1,30	2,53	FAIL
GPI	P06744	glucose-6-phosphate isomerase	1,30	2,86	FAIL
DHX36	Q9H2U1	DEAH-box helicase 36	1,30	2,03	FAIL
SENP3	Q9H4L4	SUMO specific peptidase 3	1,30	1,33	FAIL
MYO1C	O00159	myosin IC	1,30	1,49	FAIL
NKRF	O15226	NFKB repressing factor	1,30	2,62	FAIL
ABCF1	Q8NE71	ATP binding cassette subfamily F member 1	1,30	4,14	FAIL
PHF2	O75151	PHD finger protein 2	1,30	2,09	FAIL
ZNF446	Q9NWS9	zinc finger protein 446	1,30	1,85	FAIL
DDX1	Q92499	DEAD-box helicase 1	1,30	3,76	FAIL
CYTH1	K7ENQ8	cytohesin 1	1,29	1,73	FAIL
NECTIN1	Q15223	nectin cell adhesion molecule 1	1,29	2,80	FAIL
ARMC6	Q6NXE6	armadillo repeat containing 6	1,29	1,72	FAIL
TUBGCP3	Q96CW5	tubulin gamma complex component 3	1,29	2,74	FAIL
PDS5B	A9IYQ1	PDS5 cohesin associated factor B	1,29	1,68	FAIL
PTCD3	Q96EY7	pentatricopeptide repeat domain 3	1,29	3,09	FAIL
RARS1	P54136	arginyl-tRNA synthetase 1	1,29	3,85	FAIL
DNAJB1	P25685	DnaJ heat shock protein family (Hsp40) member B1	1,29	1,99	FAIL
DDX19A	I3L0H8	DEAD-box helicase 19A	1,29	2,65	FAIL
NAMPT	P43490	nicotinamide phosphoribosyltransferase	1,29	1,99	FAIL
COPS4	D6RAX7	COP9 signalosome subunit 4	1,28	2,05	FAIL
LBR	Q14739	lamin B receptor	1,28	3,06	FAIL
GLRX	P35754	glutaredoxin	1,28	1,31	FAIL
CHAF1B	Q13112	chromatin assembly factor 1 subunit B	1,28	2,56	FAIL

ELMO2	Q5JVZ5	engulfment and cell motility 2	1,28	1,34	FAIL
CIAO1	O76071	cytosolic iron-sulfur assembly component 1	1,28	1,45	FAIL
RFC5	P40937	replication factor C subunit 5	1,28	2,58	FAIL
NOC3L	Q8WTT2	NOC3 like DNA replication regulator	1,28	1,88	FAIL
EIF3E	P60228	eukaryotic translation initiation factor 3 subunit E	1,28	2,61	FAIL
PAXBP1	Q9Y5B6	PAX3 and PAX7 binding protein 1	1,28	1,63	FAIL
IDH3A	P50213	isocitrate dehydrogenase (NAD(+)) 3 catalytic subunit alpha	1,28	3,29	FAIL
WDR76	Q9H967	WD repeat domain 76	1,28	2,50	FAIL
GFM1	C9IZ01	G elongation factor mitochondrial 1	1,28	1,54	FAIL
EIF5B	O60841	eukaryotic translation initiation factor 5B	1,28	2,38	FAIL
RPL22	K7ERI7	ribosomal protein L22	1,28	2,63	FAIL
MARK2	E9PC69	microtubule affinity regulating kinase 2	1,27	2,25	FAIL
FAM76A	B1AJQ5	family with sequence similarity 76 member A	1,27	1,77	FAIL
BZW1	Q7L1Q6	basic leucine zipper and W2 domains 1	1,27	2,03	FAIL
PIGT	A0A1W2PNP0	phosphatidylinositol glycan anchor biosynthesis class T	1,27	2,21	FAIL
SSB	P05455	small RNA binding exonuclease protection factor La	1,27	1,52	FAIL
CYB5R1	Q9UHQ9	cytochrome b5 reductase 1	1,27	1,67	FAIL
DGKA	P23743	diacylglycerol kinase alpha	1,27	2,10	FAIL
USP42	Q9H9J4	ubiquitin specific peptidase 42	1,27	2,52	FAIL
PHF5A	Q7RTV0	PHD finger protein 5A	1,27	1,70	FAIL
WDR62	O43379	WD repeat domain 62	1,27	2,71	FAIL
FAM76B	Q5HYJ3	family with sequence similarity 76 member B	1,27	2,44	FAIL
RUNX1	Q01196	RUNX family transcription factor 1	1,27	2,45	FAIL
SNX17	Q15036	sorting nexin 17	1,27	1,62	FAIL
GNG2	P59768	G protein subunit gamma 2	1,26	2,48	FAIL
PRPS1	B1ALA9	phosphoribosyl pyrophosphate synthetase 1	1,26	2,89	FAIL
INTS7	Q9NVH2	integrator complex subunit 7	1,26	3,43	FAIL
SACS	Q9NZJ4	sacsin molecular chaperone	1,26	2,15	FAIL
RPN1	P04843	ribophorin I	1,26	2,22	FAIL
FKBP4	Q02790	FKBP prolyl isomerase 4	1,26	2,65	FAIL
CDCA8	Q53HL2	cell division cycle associated 8	1,26	2,05	FAIL
PHB2	Q99623	prohibitin 2	1,25	3,46	FAIL
CCNT1	O60563	cyclin T1	1,25	1,60	FAIL
EEFSEC	P57772	eukaryotic elongation factor, selenocysteine-tRNA specific	1,25	1,93	FAIL
DNMT1	P26358	DNA methyltransferase 1	1,25	2,01	FAIL
PRPF40A	H0YG38	pre-mRNA processing factor 40 homolog A	1,25	1,38	FAIL
CDK12	Q9NYV4	cyclin dependent kinase 12	1,25	1,69	FAIL
MRPS15	P82914	mitochondrial ribosomal protein S15	1,25	1,88	FAIL
ACTR2	P61160	actin related protein 2	1,25	2,17	FAIL
A2ML1	A8K2U0	alpha-2-macroglobulin like 1	1,25	1,70	FAIL
GTPBP4	Q9BZE4	GTP binding protein 4	1,25	2,02	FAIL
AP1M1	Q9BXS5	adaptor related protein complex 1 subunit mu 1	1,25	1,65	FAIL
URB2	Q14146	URB2 ribosome biogenesis homolog	1,24	3,08	FAIL
SP2	Q02086	Sp2 transcription factor	1,24	3,24	FAIL
CHCHD6	J3QTA6	coiled-coil-helix-coiled-coil-helix domain containing 6	1,24	2,37	FAIL

IARS2	Q9NSE4	isoleucyl-tRNA synthetase 2, mitochondrial	1,24	1,57	FAIL
AP3B1	O00203	adaptor related protein complex 3 subunit beta 1	1,24	3,17	FAIL
AP3D1	O14617	adaptor related protein complex 3 subunit delta 1	1,24	2,56	FAIL
ACO1	P21399	aconitase 1	1,24	1,65	FAIL
XRN2	Q9H0D6	5'-3' exoribonuclease 2	1,24	1,84	FAIL
PUM3	Q15397	pumilio RNA binding family member 3	1,24	1,63	FAIL
HNRNPU	Q00839	heterogeneous nuclear ribonucleoprotein U	1,23	4,53	FAIL
NDUFA5	Q16718	NADH:ubiquinone oxidoreductase subunit A5	1,23	2,37	FAIL
TKT	P29401	transketolase	1,23	3,51	FAIL
BDH1	Q02338	3-hydroxybutyrate dehydrogenase 1	1,23	1,95	FAIL
PKM	P14618	pyruvate kinase M1/2	1,23	2,04	FAIL
PDIA6	Q15084	protein disulfide isomerase family A member 6	1,23	1,49	FAIL
AP3S1	Q92572	adaptor related protein complex 3 subunit sigma 1	1,23	2,34	FAIL
MTCL1	Q9Y4B5	microtubule crosslinking factor 1	1,23	1,41	FAIL
XAB2	Q9HCS7	XPA binding protein 2	1,23	1,81	FAIL
GPAT4	Q86UL3	glycerol-3-phosphate acyltransferase 4	1,23	1,42	FAIL
	A0A140TA45		1,23	2,23	FAIL
PSMC6	A0A087X2I1	proteasome 26S subunit, ATPase 6	1,23	2,08	FAIL
U2SURP	O15042	U2 snRNP associated SURP domain containing	1,23	2,86	FAIL
GTF2H2	Q13888	general transcription factor IIH subunit 2	1,23	2,54	FAIL
UQCRC2	P22695	ubiquinol-cytochrome c reductase core protein 2	1,23	2,84	FAIL
PDS5B	Q9NTI5	PDS5 cohesin associated factor B	1,22	2,09	FAIL
BRI3BP	Q8WY22	BRI3 binding protein	1,22	1,79	FAIL
DHCR7	Q9UBM7	7-dehydrocholesterol reductase	1,22	2,70	FAIL
MRI1	Q9BV20	methylthioribose-1-phosphate isomerase 1	1,22	2,43	FAIL
SELPLG	Q14242	selectin P ligand	1,22	1,72	FAIL
ME2	P23368	malic enzyme 2	1,22	2,67	FAIL
MLH1	A0A669KAW3	mutL homolog 1	1,22	3,02	FAIL
RBM48	Q5RL73	RNA binding motif protein 48	1,22	1,38	FAIL
RPS2	P15880	ribosomal protein S2	1,22	2,04	FAIL
RPL35A	P18077	ribosomal protein L35a	1,21	2,23	FAIL
SF3B2	Q13435	splicing factor 3b subunit 2	1,21	1,49	FAIL
SAE1	Q9UBE0	SUMO1 activating enzyme subunit 1	1,21	3,55	FAIL
DBR1	Q9UK59	debranching RNA lariats 1	1,21	1,89	FAIL
GTF2H4	Q92759	general transcription factor IIH subunit 4	1,21	2,23	FAIL
NTHL1	P78549	nth like DNA glycosylase 1	1,21	1,74	FAIL
ZNF275	A6NFS0	zinc finger protein 275	1,21	2,44	FAIL
DHX37	Q8IY37	DEAH-box helicase 37	1,20	2,21	FAIL
PRDX3	P30048	peroxiredoxin 3	1,20	2,80	FAIL
	A0A0B4J1R3		1,20	2,06	FAIL
AGL	P35573	amylo-alpha-1, 6-glucosidase, 4-alpha-glucanotransfer-ase	1,20	1,40	FAIL
CCDC47	Q96A33	coiled-coil domain containing 47	1,20	2,17	FAIL
AIFM1	O95831	apoptosis inducing factor mitochondria associated 1	1,20	2,20	FAIL
NDUFA10	O95299	NADH:ubiquinone oxidoreductase subunit A10	1,20	1,83	FAIL
NSF	P46459	N-ethylmaleimide sensitive factor, vesicle fusing ATPase	1,20	2,08	FAIL
DDX52	Q9Y2R4	DExD-box helicase 52	1,20	1,64	FAIL

PHF6	A0A0D9SGE8	PHD finger protein 6	1,20	1,99	FAIL
ELAVL1	Q15717	ELAV like RNA binding protein 1	1,20	1,44	FAIL
TMEM30A	A0A669KBF5	transmembrane protein 30A	1,20	1,82	FAIL
PSMD3	O43242	proteasome 26S subunit, non-ATPase 3	1,20	2,88	FAIL
EIF2S2	P20042	eukaryotic translation initiation factor 2 subunit beta	1,19	3,38	FAIL
SEC24C	P53992	SEC24 homolog C, COPII coat complex component	1,19	2,57	FAIL
RRP8	O43159	ribosomal RNA processing 8	1,19	2,21	FAIL
NUP153	P49790	nucleoporin 153	1,19	1,92	FAIL
HIRIP3	Q9BW71	HIRA interacting protein 3	1,19	3,06	FAIL
TMEM214	Q6NUQ4	transmembrane protein 214	1,19	2,58	FAIL
CLIC4	Q9Y696	chloride intracellular channel 4	1,19	1,79	FAIL
SF3A2	Q15428	splicing factor 3a subunit 2	1,19	2,54	FAIL
EBNA1BP2	Q99848	EBNA1 binding protein 2	1,19	1,90	FAIL
UGP2	E7EUC7	UDP-glucose pyrophosphorylase 2	1,19	2,73	FAIL
RBM33	Q96EV2	RNA binding motif protein 33	1,19	1,69	FAIL
COQ5	Q5HYK3	coenzyme Q5, methyltransferase	1,19	2,23	FAIL
STAU2	E7EVJ4	staufen double-stranded RNA binding protein 2	1,19	2,19	FAIL
TP53RK	Q96S44	TP53 regulating kinase	1,19	3,06	FAIL
MCM6	Q14566	minichromosome maintenance complex component 6	1,19	3,55	FAIL
RSL1D1	O76021	ribosomal L1 domain containing 1	1,19	2,66	FAIL
CBFB	Q13951	core-binding factor subunit beta	1,18	1,50	FAIL
EIF2D	P41214	eukaryotic translation initiation factor 2D	1,18	1,95	FAIL
ARFRP1	Q13795	ADP ribosylation factor related protein 1	1,18	1,96	FAIL
PELO	Q9BRX2	pelota mRNA surveillance and ribosome rescue factor	1,18	1,98	FAIL
PITHD1	Q9GZP4	PITH domain containing 1	1,18	2,51	FAIL
GNG5	P63218	G protein subunit gamma 5	1,18	2,64	FAIL
LYAR	Q9NX58	Ly1 antibody reactive	1,18	1,98	FAIL
ETFA	H0YK49	electron transfer flavoprotein subunit alpha	1,18	3,07	FAIL
AKAP8L	Q9ULX6	A-kinase anchoring protein 8 like	1,18	2,74	FAIL
ARHGEF2	V9GYM8	Rho/Rac guanine nucleotide exchange factor 2	1,18	2,76	FAIL
NUDT5	A6NFX8	nudix hydrolase 5	1,17	2,77	FAIL
ATAD1	Q8NBU5	ATPase family AAA domain containing 1	1,17	1,72	FAIL
ATP5F1A	P25705	ATP synthase F1 subunit alpha	1,17	3,89	FAIL
TMEM245	Q9H330	transmembrane protein 245	1,17	1,84	FAIL
TTC14	Q96N46	tetratricopeptide repeat domain 14	1,17	1,39	FAIL
SUN2	Q9UH99	Sad1 and UNC84 domain containing 2	1,17	1,78	FAIL
RPL6	Q02878	ribosomal protein L6	1,16	2,26	FAIL
PUM1	Q5T1Z8	pumilio RNA binding family member 1	1,16	3,16	FAIL
APOO	G3V1B6	apolipoprotein O	1,16	1,72	FAIL
DNAH5	Q8TE73	dynein axonemal heavy chain 5	1,16	2,58	FAIL
UBR1	Q8IWV7	ubiquitin protein ligase E3 component n-recognin 1	1,16	2,08	FAIL
REXO4	Q9GZR2	REX4 homolog, 3'-5' exonuclease	1,16	3,30	FAIL
SRSF5	Q13243	serine and arginine rich splicing factor 5	1,16	1,67	FAIL
AGPS	O00116	alkylglycerone phosphate synthase	1,16	1,55	FAIL
MRPL37	Q9BZE1	mitochondrial ribosomal protein L37	1,16	2,20	FAIL
PPIH	C9JQD4	peptidylprolyl isomerase H	1,16	3,10	FAIL
TFAM	Q00059	transcription factor A, mitochondrial	1,16	1,76	FAIL
RACK1	P63244	receptor for activated C kinase 1	1,16	3,12	FAIL

GPAT3	Q53EU6	glycerol-3-phosphate acyltransferase 3	1,16	1,40	FAIL
RABL3	Q5HYI8	RAB, member of RAS oncogene family like 3	1,16	1,93	FAIL
MOSPD2	Q8NHP6	motile sperm domain containing 2	1,15	1,62	FAIL
TYK2	E9PM19	tyrosine kinase 2	1,15	3,72	FAIL
FARSA	K7ER00	phenylalanyl-tRNA synthetase subunit alpha	1,15	2,47	FAIL
RPS11	P62280	ribosomal protein S11	1,15	2,23	FAIL
PRPF6	O94906	pre-mRNA processing factor 6	1,15	1,92	FAIL
GCAT	O75600	glycine C-acetyltransferase	1,15	2,56	FAIL
TFIP11	Q9UBB9	tuftelin interacting protein 11	1,15	2,05	FAIL
TPI1	P60174	triosephosphate isomerase 1	1,15	2,48	FAIL
VIM	P08670	vimentin	1,15	2,13	FAIL
SEC31A	D6REX3	SEC31 homolog A, COPII coat complex component	1,15	2,15	FAIL
RPL18A	M0R3D6	ribosomal protein L18a	1,15	2,65	FAIL
LYPLA2	Q5QPQ0	lysophospholipase 2	1,14	2,40	FAIL
NEK7	Q8TDX7	NIMA related kinase 7	1,14	2,88	FAIL
PTBP3	O95758	polypyrimidine tract binding protein 3	1,14	1,57	FAIL
FCF1	Q9Y324	FCF1 rRNA-processing protein	1,14	2,03	FAIL
RPL12	P30050	ribosomal protein L12	1,14	1,87	FAIL
MYEF2	A0A0A0MQW0	myelin expression factor 2	1,14	2,31	FAIL
TAF5L	O75529	TATA-box binding protein associated factor 5 like	1,14	2,20	FAIL
CCDC83	Q8IWF9	coiled-coil domain containing 83	1,13	1,57	FAIL
DDX47	Q9H0S4	DEAD-box helicase 47	1,13	2,67	FAIL
CAPN1	P07384	calpain 1	1,13	2,85	FAIL
PRKCH	P24723	protein kinase C eta	1,13	1,39	FAIL
PTRH1	A0A096LP22	peptidyl-tRNA hydrolase 1 homolog	1,13	1,47	FAIL
TRAF3IP1	Q8TDR0	TRAF3 interacting protein 1	1,13	1,46	FAIL
PBRM1	Q86U86	polybromo 1	1,13	1,81	FAIL
PECAM1	P16284	platelet and endothelial cell adhesion molecule 1	1,13	2,90	FAIL
ORC2	Q13416	origin recognition complex subunit 2	1,13	1,65	FAIL
RPS3A	P61247	ribosomal protein S3A	1,12	3,23	FAIL
GABPA	Q06546	GA binding protein transcription factor subunit alpha	1,12	1,66	FAIL
NPEPPS	E9PLK3	aminopeptidase puromycin sensitive	1,12	2,67	FAIL
ALDH18A1	P54886	aldehyde dehydrogenase 18 family member A1	1,12	3,29	FAIL
NXF1	Q9UBU9	nuclear RNA export factor 1	1,12	2,97	FAIL
PUM2	Q8TB72	pumilio RNA binding family member 2	1,12	1,92	FAIL
CDK5RAP2	Q96SN8	CDK5 regulatory subunit associated protein 2	1,12	2,74	FAIL
RPL10	F8W7C6	ribosomal protein L10	1,12	2,53	FAIL
DGCR8	Q8WYQ5	DGCR8 microprocessor complex subunit	1,12	2,07	FAIL
RPL4	P36578	ribosomal protein L4	1,11	2,09	FAIL
APC	P25054	APC regulator of WNT signaling pathway	1,11	1,73	FAIL
TINF2	B4DFJ1	TERF1 interacting nuclear factor 2	1,11	1,72	FAIL
PA2G4	Q9UQ80	proliferation-associated 2G4	1,11	1,67	FAIL
RPA1	P27694	replication protein A1	1,11	1,85	FAIL
RPS16	P62249	ribosomal protein S16	1,11	1,50	FAIL
TRAPPC10	P48553	trafficking protein particle complex subunit 10	1,11	2,26	FAIL
INTS10	Q9NVR2	integrator complex subunit 10	1,11	2,48	FAIL
EXOSC4	Q9NPD3	exosome component 4	1,11	2,57	FAIL

DEK	H0Y993	DEK proto-oncogene	1,11	2,45	FAIL
NDUFV2	E7EPT4	NADH:ubiquinone oxidoreductase core subunit V2	1,10	1,49	FAIL
RPL3	P39023	ribosomal protein L3	1,10	1,71	FAIL
HTT	P42858	huntingtin	1,10	1,50	FAIL
EIF3D	O15371	eukaryotic translation initiation factor 3 subunit D	1,10	1,53	FAIL
BRD8	Q9H0E9	bromodomain containing 8	1,10	1,71	FAIL
RAN	B5MDF5	RAN, member RAS oncogene family	1,10	3,24	FAIL
KHDRBS1	Q07666	KH RNA binding domain containing, signal transduction associated 1	1,10	2,52	FAIL
IDH2	P48735	isocitrate dehydrogenase (NADP(+)) 2	1,10	2,54	FAIL
DAD1	F5GXX5	defender against cell death 1	1,10	1,53	FAIL
MYL12B	O14950	myosin light chain 12B	1,10	1,68	FAIL
GTPBP2	Q9BX10	GTP binding protein 2	1,10	2,18	FAIL
CACNG6	Q9BXT2	calcium voltage-gated channel auxiliary subunit gamma 6	1,09	2,44	FAIL
CDKN2AIP	Q9NXV6	CDKN2A interacting protein	1,09	2,26	FAIL
IGHMBP2	P38935	immunoglobulin mu DNA binding protein 2	1,09	1,91	FAIL
MRPS16	A6ND22	mitochondrial ribosomal protein S16	1,09	1,55	FAIL
RPL34	P49207	ribosomal protein L34	1,09	3,28	FAIL
RBM15B	Q8NDT2	RNA binding motif protein 15B	1,08	2,30	FAIL
MMAA	Q8IVH4	metabolism of cobalamin associated A	1,08	1,63	FAIL
CDC42BPA	Q5VT25	CDC42 binding protein kinase alpha	1,08	1,51	FAIL
ERAL1	O75616	Era like 12S mitochondrial rRNA chaperone 1	1,08	2,24	FAIL
CNP	P09543	2',3'-cyclic nucleotide 3' phosphodiesterase	1,08	2,89	FAIL
CKAP2	Q8WWK9	cytoskeleton associated protein 2	1,08	1,71	FAIL
PDK3	Q15120	pyruvate dehydrogenase kinase 3	1,08	1,95	FAIL
DKC1	O60832	dyskerin pseudouridine synthase 1	1,08	2,15	FAIL
SSR1	A0A3B3IRT8	signal sequence receptor subunit 1	1,08	1,76	FAIL
PRPF4	O43172	pre-mRNA processing factor 4	1,08	2,00	FAIL
COX7B	P24311	cytochrome c oxidase subunit 7B	1,07	1,51	FAIL
NOL6	Q9H6R4	nucleolar protein 6	1,07	1,91	FAIL
NDUFS3	O75489	NADH:ubiquinone oxidoreductase core subunit S3	1,07	2,22	FAIL
PARD3	Q5VWU8	par-3 family cell polarity regulator	1,07	2,53	FAIL
PPP2CA	P67775	protein phosphatase 2 catalytic subunit alpha	1,07	4,08	FAIL
HNRNPL	P14866	heterogeneous nuclear ribonucleoprotein L	1,07	1,66	FAIL
GNL1	P36915	G protein nucleolar 1 (putative)	1,07	2,07	FAIL
DTNBP1	D6RCT8	dystrobrevin binding protein 1	1,07	1,65	FAIL
SNRNP200	O75643	small nuclear ribonucleoprotein U5 subunit 200	1,06	2,22	FAIL
HNRNPA0	Q13151	heterogeneous nuclear ribonucleoprotein A0	1,06	1,79	FAIL
WDR12	Q9GZL7	WD repeat domain 12	1,06	1,56	FAIL
TARDBP	Q13148	TAR DNA binding protein	1,06	3,55	FAIL
	A0A5H1ZRP4		1,06	2,41	FAIL
COX2	P00403	cytochrome c oxidase subunit II	1,06	1,37	FAIL
UBIAD1	Q9Y5Z9	UbiA prenyltransferase domain containing 1	1,06	1,83	FAIL
CYB5R3	P00387	cytochrome b5 reductase 3	1,06	2,23	FAIL
SSR4	P51571	signal sequence receptor subunit 4	1,05	1,49	FAIL

LRWD1	Q9UFC0	leucine rich repeats and WD repeat domain containing 1	1,05	2,66	FAIL
NUP35	Q8NFH5	nucleoporin 35	1,05	2,28	FAIL
GOSR2	I3L4Z6	golgi SNAP receptor complex member 2	1,05	2,00	FAIL
CRNKL1	Q9BZJ0	crooked neck pre-mRNA splicing factor 1	1,05	2,16	FAIL
ESD	U3KQT1	esterase D	1,05	1,36	FAIL
IWS1	Q96ST2	interacts with SUPT6H, CTD assembly factor 1	1,05	2,98	FAIL
IFI16	Q16666	interferon gamma inducible protein 16	1,04	3,96	FAIL
REXO2	H0YGR4	RNA exonuclease 2	1,04	2,22	FAIL
HELLS	Q9NRZ9	helicase, lymphoid specific	1,04	1,67	FAIL
DCAF13	A0A087X1F8	DDB1 and CUL4 associated factor 13	1,04	1,67	FAIL
PDHB	P11177	pyruvate dehydrogenase E1 subunit beta	1,04	2,34	FAIL
AHSA1	O95433	activator of HSP90 ATPase activity 1	1,04	2,17	FAIL
RFC3	P40938	replication factor C subunit 3	1,04	3,06	FAIL
RPL11	Q5VVC8	ribosomal protein L11	1,04	2,52	FAIL
PRKAR1A	P10644	protein kinase cAMP-dependent type I regulatory sub-unit alpha	1,04	2,14	FAIL
PYCR2	Q96C36	pyrroline-5-carboxylate reductase 2	1,04	1,34	FAIL
PRPF31	Q8WWY3	pre-mRNA processing factor 31	1,04	1,93	FAIL
PSMB8	A0A140T998	proteasome 20S subunit beta 8	1,03	1,34	FAIL
ZNF32	P17041	zinc finger protein 32	1,03	1,84	FAIL
PCF11	O94913	PCF11 cleavage and polyadenylation factor subunit	1,03	1,63	FAIL
RTF1	Q92541	RTF1 homolog, Paf1/RNA polymerase II complex com-ponent	1,03	3,37	FAIL
ILF3	Q12906	interleukin enhancer binding factor 3	1,03	2,20	FAIL
LSM14A	Q8ND56	LSM14A mRNA processing body assembly factor	1,03	1,37	FAIL
TCF7	P36402	transcription factor 7	1,03	1,61	FAIL
RACGAP1	Q9H0H5	Rac GTPase activating protein 1	1,03	2,85	FAIL
ATXN7L3B	Q96GX2	ataxin 7 like 3B	1,02	1,48	FAIL
HSP90B1	P14625	heat shock protein 90 beta family member 1	1,02	1,41	FAIL
ERCC3	P19447	ERCC excision repair 3, TFIIH core complex helicase subunit	1,02	1,88	FAIL
GNB1L	Q9BYB4	G protein subunit beta 1 like	1,02	2,00	FAIL
PNN	Q9H307	pinin, desmosome associated protein	1,02	1,98	FAIL
SNTB2	Q13425	syntrophin beta 2	1,02	1,39	FAIL
RPS7	P62081	ribosomal protein S7	1,01	2,14	FAIL
PPHLN1	F8W6A0	periphilin 1	1,01	1,81	FAIL
CLIP1	P30622	CAP-Gly domain containing linker protein 1	1,01	1,96	FAIL
RRP1	P56182	ribosomal RNA processing 1	1,01	1,93	FAIL
MTX1	Q13505	metaxin 1	1,01	2,21	FAIL
HNRNPA3	P51991	heterogeneous nuclear ribonucleoprotein A3	1,01	1,51	FAIL
SNRPA1	P09661	small nuclear ribonucleoprotein polypeptide A'	1,00	1,68	FAIL
SAFB2	Q14151	scaffold attachment factor B2	1,00	1,50	FAIL
PNISR	Q8TF01	PNN interacting serine and arginine rich protein	1,00	2,16	FAIL
HSDL2	Q6YN16	hydroxysteroid dehydrogenase like 2	1,00	1,80	FAIL
SIKE1	Q9BRV8	suppressor of IKBKE 1	1,00	2,13	FAIL

ACAT1	A0A5F9ZHL1	acetyl-CoA acetyltransferase 1	1,00	1,57	FAIL
PSMA2	P25787	proteasome 20S subunit alpha 2	1,00	1,94	FAIL
NDUFC2	E9PRJ5	NADH:ubiquinone oxidoreductase subunit C2	1,00	2,53	FAIL
ATP5PO	P48047	ATP synthase peripheral stalk subunit OSCP	1,00	1,71	FAIL
PPP2R2D	Q66LE6	protein phosphatase 2 regulatory subunit Bdelta	1,00	2,76	FAIL
HDHD5	Q9BXW7	haloacid dehalogenase like hydrolase domain containing 5	1,00	3,33	FAIL
DPH2	Q9BQC3	diphthamide biosynthesis 2	1,00	2,66	FAIL
QARS1	P47897	glutaminyl-tRNA synthetase 1	1,00	2,46	FAIL
RPS26	P62854	ribosomal protein S26	1,00	2,51	FAIL
CDC123	O75794	cell division cycle 123	0,99	1,70	FAIL
SNRNP48	Q6IEG0	small nuclear ribonucleoprotein U11/U12 subunit 48	0,99	1,73	FAIL
TRAP1	Q12931	TNF receptor associated protein 1	0,99	1,92	FAIL
RPL7	P18124	ribosomal protein L7	0,99	1,34	FAIL
GSS	P48637	glutathione synthetase	0,99	1,89	FAIL
ZBTB11	O95625	zinc finger and BTB domain containing 11	0,98	1,31	FAIL
ZC3H4	Q9UPT8	zinc finger CCCH-type containing 4	0,98	1,64	FAIL
KAT7	O95251	lysine acetyltransferase 7	0,98	1,57	FAIL
DMAC2	M0R078	distal membrane arm assembly component 2	0,98	1,62	FAIL
KRT10	A0A1B0GVI3	keratin 10	0,98	1,34	FAIL
NCOA7	Q8NI08	nuclear receptor coactivator 7	0,97	1,79	FAIL
LGALS3BP	Q08380	galectin 3 binding protein	0,97	1,59	FAIL
EIF3A	Q14152	eukaryotic translation initiation factor 3 subunit A	0,97	1,39	FAIL
RPL5	A0A2R8Y6J3	ribosomal protein L5	0,97	2,97	FAIL
C19orf47	Q8N9M1	chromosome 19 open reading frame 47	0,97	1,37	FAIL
LAMP2	P13473	lysosomal associated membrane protein 2	0,97	1,72	FAIL
RPN2	P04844	ribophorin II	0,97	1,83	FAIL
LTV1	Q96GA3	LTV1 ribosome biogenesis factor	0,97	1,70	FAIL
RPS25	P62851	ribosomal protein S25	0,97	2,26	FAIL
ATP6V1G1	O75348	ATPase H+ transporting V1 subunit G1	0,97	1,34	FAIL
NOP2	P46087	NOP2 nucleolar protein	0,96	2,22	FAIL
NUP160	Q12769	nucleoporin 160	0,96	1,67	FAIL
HNRNPH1	P31943	heterogeneous nuclear ribonucleoprotein H1	0,96	1,61	FAIL
SREK1	A0A087WY03	splicing regulatory glutamic acid and lysine rich protein 1	0,96	3,10	FAIL
DNAJA2	O60884	DnaJ heat shock protein family (Hsp40) member A2	0,96	1,94	FAIL
RBBP6	Q7Z6E9	RB binding protein 6, ubiquitin ligase	0,96	1,92	FAIL
CEP97	Q8IW35	centrosomal protein 97	0,95	1,64	FAIL
KPNA6	O60684	karyopherin subunit alpha 6	0,95	1,52	FAIL
SPEG	Q15772	striated muscle enriched protein kinase	0,95	1,80	FAIL
TRMT5	Q32P41	tRNA methyltransferase 5	0,95	1,57	FAIL
ATP6V1H	Q9UI12	ATPase H+ transporting V1 subunit H	0,95	3,47	FAIL
RPS14	A0A2R8Y811	ribosomal protein S14	0,94	2,22	FAIL
IRF2BP1	Q8IU81	interferon regulatory factor 2 binding protein 1	0,94	2,15	FAIL
C1orf87	Q8N0U7	chromosome 1 open reading frame 87	0,94	2,85	FAIL
RUVBL1	Q9Y265	RuvB like AAA ATPase 1	0,94	1,72	FAIL
INCENP	Q9NQS7	inner centromere protein	0,94	1,98	FAIL
TPX2	Q9ULW0	TPX2 microtubule nucleation factor	0,94	1,70	FAIL

SHMT2	P34897	serine hydroxymethyltransferase 2	0,94	2,17	FAIL
HNRNPM	A0A087X0X3	heterogeneous nuclear ribonucleoprotein M	0,94	1,77	FAIL
SCAF1	Q9H7N4	SR-related CTD associated factor 1	0,94	1,88	FAIL
JAK1	A0A5F9ZH07	Janus kinase 1	0,94	1,37	FAIL
DNAJB12	Q9NXW2	DnaJ heat shock protein family (Hsp40) member B12	0,94	2,79	FAIL
WDCP	Q9H6R7	WD repeat and coiled coil containing	0,94	1,50	FAIL
CKAP5	Q14008	cytoskeleton associated protein 5	0,94	1,87	FAIL
PFKFB3	A0A1W2PR17	6-phosphofructo-2-kinase/fructose-2,6-biphosphatase 3	0,94	2,44	FAIL
PNP	P00491	purine nucleoside phosphorylase	0,94	3,23	FAIL
MTA2	O94776	metastasis associated 1 family member 2	0,93	1,67	FAIL
LGALS9	O00182	galectin 9	0,93	2,31	FAIL
OAT	P04181	ornithine aminotransferase	0,93	2,02	FAIL
	D3DQV9		0,93	1,65	FAIL
PABPN1	G3V4T2	poly(A) binding protein nuclear 1	0,93	1,62	FAIL
PAN3	Q58A45	poly(A) specific ribonuclease subunit PAN3	0,93	2,54	FAIL
GNE	Q9Y223	glucosamine (UDP-N-acetyl)-2-epimerase/N-acetyl-mannosamine kinase	0,92	2,16	FAIL
THOC2	Q8NI27	THO complex subunit 2	0,92	2,69	FAIL
MCM5	P33992	minichromosome maintenance complex component 5	0,91	1,46	FAIL
TOMM34	Q15785	translocase of outer mitochondrial membrane 34	0,91	1,32	FAIL
RPL23	P62829	ribosomal protein L23	0,91	2,01	FAIL
VRK1	Q99986	VRK serine/threonine kinase 1	0,91	2,06	FAIL
SH2B1	Q9NRF2	SH2B adaptor protein 1	0,91	1,72	FAIL
FIP1L1	Q6UN15	factor interacting with PAPOLA and CPSF1	0,91	2,59	FAIL
ARHGAP45	Q92619	Rho GTPase activating protein 45	0,91	2,35	FAIL
SYAP1	Q96A49	synapse associated protein 1	0,91	1,50	FAIL
RPS10	P46783	ribosomal protein S10	0,91	2,17	FAIL
KRR1	Q13601	KRR1 small subunit processome component homolog	0,91	3,05	FAIL
DHRS1	Q96LJ7	dehydrogenase/reductase 1	0,90	1,39	FAIL
MTFMT	Q96DP5	mitochondrial methionyl-tRNA formyltransferase	0,90	1,32	FAIL
IKZF1	Q13422	IKAROS family zinc finger 1	0,90	2,55	FAIL
THOC7	Q6I9Y2	THO complex subunit 7	0,90	1,36	FAIL
RPL29	A0A3B3ITT5	ribosomal protein L29	0,90	3,22	FAIL
AHNAK	Q09666	AHNAK nucleoprotein	0,90	3,23	FAIL
DCTN5	H3BUD2	dynactin subunit 5	0,90	2,49	FAIL
GPKOW	Q92917	G-patch domain and KOW motifs	0,90	2,48	FAIL
YY1	P25490	YY1 transcription factor	0,89	1,41	FAIL
KIFC1	Q9BW19	kinesin family member C1	0,89	1,44	FAIL
MOV10	Q9HCE1	Mov10 RNA helicase	0,89	4,13	FAIL
HSPD1	P10809	heat shock protein family D (Hsp60) member 1	0,89	1,58	FAIL
STRBP	Q96SI9	spermatid perinuclear RNA binding protein	0,89	2,33	FAIL
NUDT3	O95989	nudix hydrolase 3	0,89	1,33	FAIL
HDGFL2	Q7Z4V5	HDGF like 2	0,88	1,53	FAIL
ETF1	B7Z7P8	eukaryotic translation termination factor 1	0,88	2,17	FAIL
ERP29	F8VY02	endoplasmic reticulum protein 29	0,88	1,31	FAIL
SPOUT1	Q5T280	SPOUT domain containing methyltransferase 1	0,88	3,02	FAIL
CLK3	P49761	CDC like kinase 3	0,87	2,79	FAIL

NSUN2	Q08J23	NOP2/Sun RNA methyltransferase 2	0,87	1,33	FAIL
RO60	P10155	Ro60, Y RNA binding protein	0,87	1,45	FAIL
MSMO1	Q15800	methylsterol monooxygenase 1	0,87	2,04	FAIL
TXNDC11	Q6PKC3	thioredoxin domain containing 11	0,86	2,63	FAIL
H1-5	P16401	H1.5 linker histone, cluster member	0,86	1,88	FAIL
SURF6	O75683	surfeit 6	0,86	1,69	FAIL
METAP2	P50579	methionyl aminopeptidase 2	0,86	1,70	FAIL
N4BP1	O75113	NEDD4 binding protein 1	0,85	2,53	FAIL
NHERF4	E9PPZ1	NHERF family PDZ scaffold protein 4	0,84	1,45	FAIL
STIL	E9PSF2	STIL centriolar assembly protein	0,84	1,30	FAIL
OCIAD2	Q56VL3	OCIA domain containing 2	0,84	2,04	FAIL
RBMX	P38159	RNA binding motif protein X-linked	0,84	1,40	FAIL
FADS2	O95864	fatty acid desaturase 2	0,84	4,30	FAIL
	H3BR70		0,84	1,89	FAIL
DDX10	Q13206	DEAD-box helicase 10	0,84	2,56	FAIL
SRRT	Q9BXP5	serrate, RNA effector molecule	0,83	1,39	FAIL
	A0A0G2JHC2		0,83	1,76	FAIL
MCM3	P25205	minichromosome maintenance complex component 3	0,83	2,33	FAIL
SLAIN1	A0A0A0MSS3	SLAIN motif family member 1	0,83	1,92	FAIL
RPS18	P62269	ribosomal protein S18	0,83	1,38	FAIL
UPF2	Q9HAU5	UPF2 regulator of nonsense mediated mRNA decay	0,83	2,03	FAIL
DHX8	Q14562	DEAH-box helicase 8	0,82	2,19	FAIL
PMVK	Q15126	phosphomevalonate kinase	0,82	2,11	FAIL
AKAP1	Q92667	A-kinase anchoring protein 1	0,82	2,16	FAIL
TMED10	P49755	transmembrane p24 trafficking protein 10	0,82	1,95	FAIL
CHI3L2	Q15782	chitinase 3 like 2	0,81	1,56	FAIL
CDR2	H3BU23	cerebellar degeneration related protein 2	0,81	1,82	FAIL
	A0A4V7I447		0,81	1,65	FAIL
	F8WCL7		0,80	1,56	FAIL
MRPL54	Q6P161	mitochondrial ribosomal protein L54	0,80	1,95	FAIL
SFSWAP	Q12872	splicing factor SWAP	0,79	2,41	FAIL
MYO1G	B0I1T2	myosin IG	0,79	2,14	FAIL
THOC1	Q96FV9	THO complex subunit 1	0,79	1,32	FAIL
CDK5	Q00535	cyclin dependent kinase 5	0,79	1,46	FAIL
CRYZ	C9JH92	crystallin zeta	0,78	1,67	FAIL
EIF6	P56537	eukaryotic translation initiation factor 6	0,78	1,67	FAIL
HJURP	C9JWC4	Holliday junction recognition protein	0,78	1,58	FAIL
L2HGDH	Q9H9P8	L-2-hydroxyglutarate dehydrogenase	0,77	1,85	FAIL
LRCH1	Q9Y2L9	leucine rich repeats and calponin homology domain containing 1	0,77	1,90	FAIL
CORO7-PAM16	A0A0A6YYL4	CORO7-PAM16 readthrough	0,77	1,74	FAIL
PAF1	Q8N7H5	PAF1 homolog, Paf1/RNA polymerase II complex component	0,76	1,32	FAIL
SAMD13	Q5VXD3	sterile alpha motif domain containing 13	0,76	2,51	FAIL
TRAF3IP3	Q9Y228	TRAF3 interacting protein 3	0,75	1,56	FAIL
	B9ZVT1		0,75	1,53	FAIL

HELZ	P42694	helicase with zinc finger	0,74	1,82	FAIL
RABGAP1	Q9Y3P9	RAB GTPase activating protein 1	0,74	2,30	FAIL
PSMB1	P20618	proteasome 20S subunit beta 1	0,73	1,70	FAIL
FDXR	A0A0A0MT64	ferredoxin reductase	0,73	2,35	FAIL
TXNDC5	Q8NBS9	thioredoxin domain containing 5	0,73	1,75	FAIL
SNRPD3	P62318	small nuclear ribonucleoprotein D3 polypeptide	0,72	2,73	FAIL
RPL7L1	Q6DKI1	ribosomal protein L7 like 1	0,72	1,31	FAIL
RAB8A	P61006	RAB8A, member RAS oncogene family	0,72	1,48	FAIL
AURKA	O14965	aurora kinase A	0,72	1,37	FAIL
TRAF7	Q6Q0C0	TNF receptor associated factor 7	0,72	1,83	FAIL
NSUN4	Q96CB9	NOP2/Sun RNA methyltransferase 4	0,71	1,82	FAIL
MCIDAS	D6RGH6	multiciliate differentiation and DNA synthesis associated cell cycle protein	0,70	3,39	FAIL
TARBP1	Q13395	TAR (HIV-1) RNA binding protein 1	0,70	1,86	FAIL
AVIL	O75366	advillin	0,69	2,03	FAIL
CEP170B	Q9Y4F5	centrosomal protein 170B	0,69	1,44	FAIL
HNRNPA2B1	P22626	heterogeneous nuclear ribonucleoprotein A2/B1	0,69	1,49	FAIL
NOL10	Q9BSC4	nucleolar protein 10	0,69	1,45	FAIL
NCL	P19338	nucleolin	0,68	2,36	FAIL
MRPL14	Q6P1L8	mitochondrial ribosomal protein L14	0,68	1,44	FAIL
RSU1	Q15404	Ras suppressor protein 1	0,67	1,74	FAIL
DDX3X	O00571	DEAD-box helicase 3 X-linked	0,66	1,60	FAIL
ZKSCAN1	P17029	zinc finger with KRAB and SCAN domains 1	0,66	1,85	FAIL
ACTL6A	O96019	actin like 6A	0,66	1,44	FAIL
FXR1	B4DXZ6	FMR1 autosomal homolog 1	0,66	1,70	FAIL
ZC3H11A	O75152	zinc finger CCCH-type containing 11A	0,65	1,55	FAIL
ABCD3	A0A3B3ITW3	ATP binding cassette subfamily D member 3	0,65	2,08	FAIL
RBM5	P52756	RNA binding motif protein 5	0,65	1,40	FAIL
NOB1	Q9ULX3	NIN1 (RPN12) binding protein 1 homolog	0,65	1,32	FAIL
PRXL2A	Q9BRX8	peroxiredoxin like 2A	0,65	1,56	FAIL
HBA2	A0A2R8Y7C0	hemoglobin subunit alpha 2	0,65	1,79	FAIL
CDC20	Q12834	cell division cycle 20	0,64	1,56	FAIL
COIL	P38432	coilin	0,64	1,38	FAIL
NCOA5	Q9HCD5	nuclear receptor coactivator 5	0,63	2,09	FAIL
RPS4X	P62701	ribosomal protein S4 X-linked	0,63	1,56	FAIL
DDX46	Q7L014	DEAD-box helicase 46	0,62	3,86	FAIL
HP1BP3	Q5SSJ5	heterochromatin protein 1 binding protein 3	0,62	1,35	FAIL
RPL36	Q9Y3U8	ribosomal protein L36	0,61	1,52	FAIL
MATR3	A8MXP9	matrin 3	0,59	1,72	FAIL
ZNF24	P17028	zinc finger protein 24	0,59	1,94	FAIL
NMNAT1	Q9HAN9	nicotinamide nucleotide adenylyltransferase 1	0,58	1,54	FAIL
DNAJC7	A0A6I8PU73	DnaJ heat shock protein family (Hsp40) member C7	0,58	1,37	FAIL
HLCS	P50747	holocarboxylase synthetase	0,58	2,94	FAIL
TUFM	P49411	Tu translation elongation factor, mitochondrial	0,57	1,32	FAIL
ABCB7	A0A087WW65	ATP binding cassette subfamily B member 7	0,55	2,44	FAIL
CCDC82	A0A2R8YE98	coiled-coil domain containing 82	0,55	1,74	FAIL
RPL27A	E9PLL6	ribosomal protein L27a	0,55	1,55	FAIL

GSTK1	Q9Y2Q3	glutathione S-transferase kappa 1	0,53	1,33	FAIL
RPL26	P61254	ribosomal protein L26	0,53	1,35	FAIL
CCDC59	Q9P031	coiled-coil domain containing 59	0,52	1,35	FAIL
RPL10A	P62906	ribosomal protein L10a	0,52	1,33	FAIL
TAF6L	Q9Y6J9	TATA-box binding protein associated factor 6 like	0,51	2,07	FAIL
NAXE	Q8NCW5	NAD(P)HX epimerase	0,50	1,62	FAIL
PRPF4B	Q13523	pre-mRNA processing factor 4B	0,50	1,38	FAIL
DECR1	Q16698	2,4-dienoyl-CoA reductase 1	0,49	1,38	FAIL
PLA2G4E	Q3MJ16	phospholipase A2 group IVE	0,49	1,57	FAIL
GLS2	A0A087X004	glutaminase 2	0,47	1,38	FAIL
RPL23A	P62750	ribosomal protein L23a	0,46	1,63	FAIL
SNRNP70	P08621	small nuclear ribonucleoprotein U1 subunit 70	0,45	1,32	FAIL
SAPCD2	Q86UD0	suppressor APC domain containing 2	0,45	1,64	FAIL
CALU	O43852	calumenin	0,43	1,80	FAIL
PUF60	A0A0J9YVP6	poly(U) binding splicing factor 60	0,41	1,66	FAIL
RBM14	Q96PK6	RNA binding motif protein 14	0,41	1,74	FAIL
MPP1	Q00013	MAGUK p55 scaffold protein 1	0,40	2,82	FAIL
FLNC	Q14315	filamin C	0,38	1,31	FAIL
UQCC5	Q8WVI0	ubiquinol-cytochrome c reductase complex assembly factor 5	0,37	1,93	FAIL
SART1	O43290	spliceosome associated factor 1, recruiter of U4/U6.U5 tri-snRNP	0,35	1,75	FAIL
SGPL1	O95470	sphingosine-1-phosphate lyase 1	0,20	1,98	FAIL
RPRD2	Q5VT52	regulation of nuclear pre-mRNA domain containing 2	-0,31	1,48	FAIL
RP9	Q8TA86	RP9 pre-mRNA splicing factor	-0,47	2,01	FAIL
TOM1	O60784	target of myb1 membrane trafficking protein	-0,49	1,43	FAIL
IMPA2	K7EL65	inositol monophosphatase 2	-0,50	1,76	FAIL
HLTF	Q14527	helicase like transcription factor	-0,51	1,39	FAIL
KRT78	Q8N1N4	keratin 78	-0,52	2,71	FAIL
ZRANB2	O95218	zinc finger RANBP2-type containing 2	-0,56	1,97	FAIL
MRPL17	Q9NRX2	mitochondrial ribosomal protein L17	-0,71	1,80	FAIL
MOB3A	Q96BX8	MOB kinase activator 3A	-0,73	1,64	FAIL
S100A7	P31151	S100 calcium binding protein A7	-0,74	1,39	FAIL
IL36G	Q9NZH8	interleukin 36 gamma	-0,74	1,51	FAIL
ARGLU1	Q9NWB6	arginine and glutamate rich 1	-0,75	2,47	FAIL
ITK	Q08881	IL2 inducible T cell kinase	-0,78	2,25	FAIL
PHKA1	A6NMN0	phosphorylase kinase regulatory subunit alpha 1	-0,80	1,56	FAIL
SREK1IP1	Q8N9Q2	SREK1 interacting protein 1	-0,81	1,66	FAIL
SCGB1D2	O95969	secretoglobin family 1D member 2	-0,82	1,86	FAIL
EEF2	P13639	eukaryotic translation elongation factor 2	-0,83	2,59	FAIL
CD151	E9PRJ3	CD151 molecule (Raph blood group)	-0,83	1,35	FAIL
GSTM1	E7EWW9	glutathione S-transferase mu 1	-0,84	1,60	FAIL
MED30	Q96HR3	mediator complex subunit 30	-0,84	1,94	FAIL
ARG1	P05089	arginase 1	-0,87	1,73	FAIL
AZGP1	P25311	alpha-2-glycoprotein 1, zinc-binding	-0,88	1,91	FAIL
PKP1	Q13835	plakophilin 1	-0,88	1,47	FAIL
DCD	P81605	dermcidin	-0,88	2,15	FAIL

AKR1B1	P15121	aldo-keto reductase family 1 member B	-0,89	2,11	FAIL
	A0A087X211		-0,91	3,17	FAIL
MAP4K4	E7EN19	mitogen-activated protein kinase kinase kinase kinase 4	-0,92	1,72	FAIL
BRD10	Q5HYC2	bromodomain containing 10	-0,93	1,66	FAIL
APOD	C9JF17	apolipoprotein D	-0,94	1,62	FAIL
SRSF9	Q13242	serine and arginine rich splicing factor 9	-0,94	1,46	FAIL
NUP205	Q92621	nucleoporin 205	-0,94	1,71	FAIL
KLHDC4	H3BQH3	kelch domain containing 4	-0,94	2,00	FAIL
RBM17	Q96I25	RNA binding motif protein 17	-0,98	1,48	FAIL
WDR70	Q9NW82	WD repeat domain 70	-0,99	1,77	FAIL
KRT77	Q7Z794	keratin 77	-1,00	1,34	FAIL
MCCC1	Q96RQ3	methylcrotonyl-CoA carboxylase subunit 1	-1,03	1,75	FAIL
PSMB7	Q99436	proteasome 20S subunit beta 7	-1,06	1,74	FAIL
FNTA	B3KVN2	farnesyltransferase, CAAX box, alpha	-1,09	1,73	FAIL
	A0A087WZC4		-1,11	1,64	FAIL
SERPINC1	P01008	serpin family C member 1	-1,16	1,75	FAIL
HERC2	O95714	HECT and RLD domain containing E3 ubiquitin protein ligase 2	-1,17	1,45	FAIL
BRD4	O60885	bromodomain containing 4	-1,18	1,72	FAIL
DNAJB14	Q8TBM8	DnaJ heat shock protein family (Hsp40) member B14	-1,18	2,06	FAIL
NCCRP1	Q6ZVX7	NCCRP1, F-box associated domain containing	-1,18	1,87	FAIL
SLC7A1	P30825	solute carrier family 7 member 1	-1,18	1,31	FAIL
KRT1	P04264	keratin 1	-1,19	3,06	FAIL
MED12L	F8WAE6	mediator complex subunit 12L	-1,19	1,57	FAIL
PCCA	P05165	propionyl-CoA carboxylase subunit alpha	-1,21	1,41	FAIL
GPATCH1	Q9BRR8	G-patch domain containing 1	-1,21	1,92	FAIL
RBBP8	F6Q6H0	RB binding protein 8, endonuclease	-1,23	2,05	FAIL
TP53BP2	Q13625	tumor protein p53 binding protein 2	-1,26	2,47	FAIL
FLG	P20930	filaggrin	-1,30	2,08	FAIL
PLEKHO1	Q53GL0	pleckstrin homology domain containing O1	-1,31	2,07	FAIL
S100A14	Q9HCY8	S100 calcium binding protein A14	-1,33	1,87	FAIL
TGM3	Q08188	transglutaminase 3	-1,35	2,62	FAIL
CDK11B	P21127	cyclin dependent kinase 11B	-1,35	1,71	FAIL
WDR91	A4D1P6	WD repeat domain 91	-1,35	2,37	FAIL
ATG5	A0A2R8Y718	autophagy related 5	-1,35	1,36	FAIL
PCCB	C9JQS9	propionyl-CoA carboxylase subunit beta	-1,36	2,14	FAIL
SERPINB12	Q96P63	serpin family B member 12	-1,37	2,73	FAIL
GSDMA	Q96QA5	gasdermin A	-1,38	2,42	FAIL
DSG1	Q02413	desmoglein 1	-1,38	1,70	FAIL
KRT14	P02533	keratin 14	-1,39	2,19	FAIL
PIKFYVE	Q9Y2I7	phosphoinositide kinase, FYVE-type zinc finger containing	-1,41	1,51	FAIL
MCCC2	Q9HCC0	methylcrotonyl-CoA carboxylase subunit 2	-1,42	1,37	FAIL
PPP6R2	O75170	protein phosphatase 6 regulatory subunit 2	-1,43	2,18	FAIL
PRSS1	E7EQ64	serine protease 1	-1,44	1,36	FAIL
SCGB2A2	Q13296	secretoglobin family 2A member 2	-1,46	2,68	FAIL

GDPD3	Q7L5L3	glycerophosphodiester phosphodiesterase domain containing 3	-1,46	1,71	FAIL
GCFC2	P16383	GC-rich sequence DNA-binding factor 2	-1,47	1,72	FAIL
DCP1A	Q9NPI6	decapping mRNA 1A	-1,47	1,32	FAIL
BRCA1	P38398	BRCA1 DNA repair associated	-1,49	2,47	FAIL
VCL	P18206	vinculin	-1,49	1,41	FAIL
MYH10	P35580	myosin heavy chain 10	-1,53	2,07	FAIL
FLII	Q13045	FLII actin remodeling protein	-1,54	2,26	FAIL
CST4	P01036	cystatin S	-1,56	1,40	FAIL
XPOT	O43592	exportin for tRNA	-1,58	1,37	FAIL
S100A16	Q96FQ6	S100 calcium binding protein A16	-1,58	2,89	FAIL
KRT2	P35908	keratin 2	-1,59	3,37	FAIL
SDR9C7	Q8NEX9	short chain dehydrogenase/reductase family 9C member 7	-1,60	1,62	FAIL
HERC1	Q15751	HECT and RLD domain containing E3 ubiquitin protein ligase family member 1	-1,62	1,81	FAIL
SRGAP3	O43295	SLIT-ROBO Rho GTPase activating protein 3	-1,62	1,49	FAIL
IAH1	Q2TAA2	isoamyl acetate hydrolyzing esterase 1 (putative)	-1,63	1,38	FAIL
PAPOLA	P51003	poly(A) polymerase alpha	-1,63	1,43	FAIL
KRT10	P13645	keratin 10	-1,64	2,50	FAIL
KPRP	Q5T749	keratinocyte proline rich protein	-1,68	1,84	FAIL
NDST4	Q9H3R1	N-deacetylase and N-sulfotransferase 4	-1,68	2,15	FAIL
	Q8IZJ6		-1,68	2,15	FAIL
DYNC2H1	Q8NCM8	dynein cytoplasmic 2 heavy chain 1	-1,70	2,17	FAIL
CHIA	Q9BZP6	chitinase acidic	-1,70	1,80	FAIL
PDHA1	P08559	pyruvate dehydrogenase E1 subunit alpha 1	-1,70	2,48	FAIL
IL20RB	Q6UXL0	interleukin 20 receptor subunit beta	-1,72	2,53	FAIL
ABI3BP	Q7Z7G0	ABI family member 3 binding protein	-1,77	1,57	FAIL
ASAP2	O43150	ArfGAP with SH3 domain, ankyrin repeat and PH domain 2	-1,79	2,60	FAIL
FLG2	Q5D862	filaggrin 2	-1,79	2,29	FAIL
GGCT	O75223	gamma-glutamylcyclotransferase	-1,81	3,39	FAIL
CCDC88B	H7BY33	coiled-coil domain containing 88B	-1,86	2,78	FAIL
LACTB	P83111	lactamase beta	-1,88	3,20	FAIL
POLD1	M0R2B7	DNA polymerase delta 1, catalytic subunit	-1,89	4,06	FAIL
KRT17	Q04695	keratin 17	-1,91	3,78	FAIL
ING5	A0A1B0GW41	inhibitor of growth family member 5	-1,93	1,69	FAIL
OSBPL1A	Q9BXW6	oxysterol binding protein like 1A	-1,95	4,04	FAIL
VPS16	Q9H269	VPS16 core subunit of CORVET and HOPS complexes	-1,96	3,74	FAIL
TGM1	P22735	transglutaminase 1	-2,09	2,48	FAIL
	A0A087WX03		-2,09	1,39	FAIL
CRLF3	J3KST8	cytokine receptor like factor 3	-2,10	2,37	FAIL
TLE1	Q04724	TLE family member 1, transcriptional corepressor	-2,10	1,97	FAIL
PDHA2	P29803	pyruvate dehydrogenase E1 subunit alpha 2	-2,12	1,71	FAIL
KRT79	Q5XKE5	keratin 79	-2,12	2,13	FAIL
MCM7	P33993	minichromosome maintenance complex component 7	-2,13	2,50	FAIL
MRM1	Q6IN84	mitochondrial rRNA methyltransferase 1	-2,14	2,64	FAIL

KCTD3	Q9Y597	potassium channel tetramerization domain containing 3	-2,51	1,69	FAIL
	A0A0G2JLB3		-2,72	1,76	FAIL
CTSV	O60911	cathepsin V	-2,88	1,32	FAIL
DST	F8W9J4	dystonin	-2,88	2,27	FAIL
NCBP3	Q53F19	nuclear cap binding subunit 3	-3,41	2,14	FAIL
RBM10	P98175	RNA binding motif protein 10	-3,56	1,50	FAIL
TF	P02787	transferrin	-3,66	1,44	FAIL
BRCA2	P51587	BRCA2 DNA repair associated	-3,78	1,85	FAIL
ACACB	O00763	acetyl-CoA carboxylase beta	-4,84	1,49	FAIL

Table S2. Gene Ontology analysis of the 34 significantly and highly enriched proteins reveals association with MVB organization and regulation of TAG sequestering and exsosome secretion.

Term	-log10 (p value)	Genes
Multivesicular Body Organization (GO:0036258)	4,65	IST1;HGS;STAM
Multivesicular Body Organization (GO:0036257)	4,61	IST1;HGS;STAM
Positive Regulation Of Sequestering Of Triglyceride (GO:0010890)	4,01	PLIN3;LCP1
Regulation Of Sequestering Of Triglyceride (GO:0010889)	3,50	PLIN3;LCP1
Positive Regulation Of Exosomal Secretion (GO:1903543)	3,50	HGS;STAM
Regulation Of Exosomal Secretion (GO:1903541)	3,28	HGS;STAM
Positive Regulation Of Lipid Storage (GO:0010884)	3,18	PLIN3;LCP1
Late Endosome To Vacuole Transport Via Multivesicular Body Sorting Pathway (GO:0032511)	2,99	HGS;STAM
Establishment Of Protein Localization To Vacuole (GO:0072666)	2,92	HGS;STAM
Regulation Of Calcium-Mediated Signaling (GO:0050848)	2,83	JPT2;GBP1
Intracellular pH Reduction (GO:0051452)	2,77	SLC4A7;ATP6V0D1
Ubiquitin-Dependent Protein Catabolic Process Via The Multivesicular Body Sorting Pathway (GO:0043162)	2,75	HGS;STAM
Purine Nucleotide Metabolic Process (GO:0006163)	2,72	SLC4A7;AMPD2
Positive Regulation Of Exocytosis (GO:0045921)	2,65	HGS;STAM
Membrane Fission (GO:0090148)	2,63	HGS;STAM
Actin Filament Organization (GO:0007015)	2,59	CNN2;TAGLN2;LCP1
Cellular Response To Glucose Starvation (GO:0042149)	2,54	SESN2;PLIN3
Organelle Assembly (GO:0070925)	2,50	IST1;HGS;STAM;ATP6V0D1
Regulation Of Protein Modification Process (GO:0031399)	2,48	SESN2;CCDC88C
Purine Ribonucleotide Biosynthetic Process (GO:0009152)	2,31	IMPDH1;AMPD2
Positive Regulation Of Proteolysis (GO:0045862)	2,25	IST1;SNX9
Plasma Membrane Organization (GO:0007009)	2,08	ANXA6;SNX9
Phagosome Acidification (GO:0090383)	2,02	SLC4A7
Regulation Of Extracellular Exosome Assembly (GO:1903551)	2,02	STAM
Regulation Of Translation In Response To Endoplasmic Reticulum Stress (GO:0036490)	2,02	SESN2
Positive Regulation Of Podosome Assembly (GO:0071803)	2,02	LCP1
Zymogen Inhibition (GO:0097341)	2,02	CAST
Inner Ear Auditory Receptor Cell Differentiation (GO:0042491)	2,02	SLC4A7
Positive Regulation Of Cholesterol Storage (GO:0010886)	2,02	LCP1
Lipid Droplet Disassembly (GO:1905691)	2,02	PLIN3
Presynaptic Active Zone Organization (GO:1990709)	2,02	CAST
Regulation Of Organelle Assembly (GO:1902115)	1,99	STAM;TBC1D15
Cytoskeleton-Dependent Cytokinesis (GO:0061640)	1,98	IST1;SNX9
Regulation Of Phosphorylation (GO:0042325)	1,97	SESN2;CCDC88C
Membrane Invagination (GO:0010324)	1,95	HGS
L-methionine Salvage (GO:0071267)	1,95	APIP
Cleavage Furrow Formation (GO:0036089)	1,95	SNX9
Response To Increased Oxygen Levels (GO:0036296)	1,95	ATP6V0D1
Antigen Processing And Presentation Of Endogenous Peptide Antigen Via MHC Class I (GO:0019885)	1,95	ERAP1
Positive Regulation Of Chemokine (C-X-C Motif) Ligand 2 Production (GO:2000343)	1,95	LCP1
IMP Biosynthetic Process (GO:0006188)	1,88	AMPD2

Very-Low-Density Lipoprotein Particle Remodeling (GO:0034372)	1,88	LCP1
Inhibition Of Cysteine-Type Endopeptidase Activity (GO:0097340)	1,88	CAST
Positive Regulation Of Cellular Component Biogenesis (GO:0044089)	1,87	HGS;STAM
Regulation Of Macroautophagy (GO:0016241)	1,84	SESN2;ATP6V0D1
AMP Biosynthetic Process (GO:0006167)	1,82	APRT
Triglyceride-Rich Lipoprotein Particle Remodeling (GO:0034370)	1,82	LCP1
Actin Filament Network Formation (GO:0051639)	1,82	LCP1
Inner Ear Receptor Cell Development (GO:0060119)	1,82	SLC4A7
Regulation Of Podosome Assembly (GO:0071801)	1,82	LCP1
Macroautophagy (GO:0016236)	1,78	HGS;STAM
ESCRT III Complex Disassembly (GO:1904903)	1,77	IST1
ESCRT Complex Disassembly (GO:1904896)	1,77	IST1
Regulation Of Sequestering Of Calcium Ion (GO:0051282)	1,77	ANXA6
IMP Metabolic Process (GO:0046040)	1,77	AMPD2
Cellular Response To Oxygen Levels (GO:0071453)	1,77	ATP6V0D1
Actin-Mediated Cell Contraction (GO:0070252)	1,77	CCDC88C
Negative Regulation Of Cellular Process (GO:0048523)	1,73	SUZ12;HGS;SESN2;ANXA6
Protein Localization To Condensed Chromosome (GO:1903083)	1,72	CHAMP1
Positive Regulation Of Lipid Localization (GO:1905954)	1,72	LCP1
Cellular Response To Increased Oxygen Levels (GO:0036295)	1,72	ATP6V0D1
Cellular Response To Leucine (GO:0071233)	1,72	SESN2
Purine Ribonucleoside Triphosphate Biosynthetic Process (GO:0009206)	1,72	IMPDH1
Response To Leucine (GO:0043201)	1,72	SESN2
Negative Regulation Of Vascular Endothelial Growth Factor Receptor Signaling Pathway (GO:0030948)	1,72	HGS
Small GTPase Mediated Signal Transduction (GO:0007264)	1,72	CCDC88C;RRAS2
AMP Metabolic Process (GO:0046033)	1,68	AMPD2
Protein Localization To Kinetochore (GO:0034501)	1,68	CHAMP1
Purine Ribonucleoside Monophosphate Biosynthetic Process (GO:0009168)	1,68	AMPD2
Purine Ribonucleoside Monophosphate Metabolic Process (GO:0009167)	1,68	AMPD2
Negative Regulation Of Platelet-Derived Growth Factor Receptor Signaling Pathway (GO:0010642)	1,68	HGS
Heterocycle Biosynthetic Process (GO:0018130)	1,68	MOCS2
Actin Crosslink Formation (GO:0051764)	1,68	LCP1
Regulation Of Adipose Tissue Development (GO:1904177)	1,68	LCP1
Supramolecular Fiber Organization (GO:0097435)	1,66	CNN2;CCDC88C;TAGLN2
Receptor-Mediated Endocytosis (GO:0006898)	1,65	HGS;SNX9
Regulation Of Chemokine (C-X-C Motif) Ligand 2 Production (GO:2000341)	1,65	LCP1
Triglyceride Catabolic Process (GO:0019433)	1,65	LCP1
Cellular Response To Leucine Starvation (GO:1990253)	1,65	SESN2
Negative Regulation Of Substrate Adhesion-Dependent Cell Spreading (GO:1900025)	1,65	GBP1
Attachment Of Mitotic Spindle Microtubules To Kinetochore (GO:0051315)	1,65	CHAMP1
Regulation Of Platelet-Derived Growth Factor Receptor Signaling Pathway (GO:0010640)	1,65	HGS
Intracellular Protein Transport (GO:0006886)	1,63	HGS;SNX9;STAM
Cellular Response To Starvation (GO:0009267)	1,62	SESN2;PLIN3
GTP Metabolic Process (GO:0046039)	1,61	IMPDH1
Plasma Membrane Tubulation (GO:0097320)	1,61	SNX9
Regulation Of Cholesterol Storage (GO:0010885)	1,58	LCP1

Protein Localization To Vacuole (GO:0072665)	1,58	GBP1
Positive Regulation Of Membrane Protein Ectodomain Proteolysis (GO:0051044)	1,58	SNX9
Protein Transport To Vacuole Involved In Ubiquitin-Dependent Protein Catabolic Process Via The Multivesicular Body Sorting Pathway (GO:0043328)	1,58	STAM
Negative Regulation Of Receptor Signaling Pathway Via STAT (GO:1904893)	1,58	HGS
Epiboly Involved In Wound Healing (GO:0090505)	1,55	LCP1
Phagosome Maturation (GO:0090382)	1,55	SLC4A7
Defense Response To Protozoan (GO:0042832)	1,55	GBP1
Protein Localization (GO:0008104)	1,54	IST1;HGS;SNX9
Protein Kinase A Signaling (GO:0010737)	1,52	LCP1
DNA-templated Transcription Termination (GO:0006353)	1,52	SMN1
Cellular Response To Fatty Acid (GO:0071398)	1,52	LCP1
Negative Regulation Of Receptor Signaling Pathway Via JAK-STAT (GO:0046426)	1,52	HGS
Positive Regulation Of Macrophage Derived Foam Cell Differentiation (GO:0010744)	1,50	LCP1
Polyol Metabolic Process (GO:0019751)	1,50	INPP4A
Purine-Containing Compound Biosynthetic Process (GO:0072522)	1,50	SLC4A7
Protein Localization To Chromosome, Centromeric Region (GO:0071459)	1,47	CHAMP1
Negative Regulation Of T Cell Receptor Signaling Pathway (GO:0050860)	1,47	GBP1
Antigen Processing And Presentation Of Peptide Antigen Via MHC Class I (GO:0002474)	1,47	ERAP1
Cortical Actin Cytoskeleton Organization (GO:0030866)	1,45	LCP1
Response To Fatty Acid (GO:0070542)	1,45	LCP1
Bicarbonate Transport (GO:0015701)	1,45	SLC4A7
Nucleotide Biosynthetic Process (GO:0009165)	1,45	SLC4A7
Regulation Of Protein Localization To Cell Periphery (GO:1904375)	1,43	GBP1
Negative Regulation Of Protein Localization To Cell Periphery (GO:1904376)	1,43	GBP1
Negative Regulation Of Protein Localization To Plasma Membrane (GO:1903077)	1,43	GBP1
Intracellular Monoatomic Ion Homeostasis (GO:0006873)	1,43	CNNM4
Peptide Catabolic Process (GO:0043171)	1,41	ERAP1
Negative Regulation Of Interleukin-2 Production (GO:0032703)	1,41	GBP1
Mitochondrial Calcium Ion Homeostasis (GO:0051560)	1,41	ANXA6
Purine Nucleotide Biosynthetic Process (GO:0006164)	1,41	SLC4A7
Regulation Of Lipid Storage (GO:0010883)	1,41	LCP1
Regulation Of Membrane Protein Ectodomain Proteolysis (GO:0051043)	1,41	SNX9
Antigen Processing And Presentation Of Endogenous Peptide Antigen (GO:0002483)	1,41	ERAP1
Lipid Catabolic Process (GO:0016042)	1,41	LCP1
Membrane Organization (GO:0061024)	1,40	HGS;STAM
Membrane Protein Ectodomain Proteolysis (GO:0006509)	1,39	ERAP1
Cellular Response To Low-Density Lipoprotein Particle Stimulus (GO:0071404)	1,39	LCP1
Regulation Of Vascular Endothelial Growth Factor Receptor Signaling Pathway (GO:0030947)	1,39	HGS
Cytoskeleton-Dependent Intracellular Transport (GO:0030705)	1,39	CCDC88C
Inositol Phosphate Metabolic Process (GO:0043647)	1,39	INPP4A
Regulation Of Catabolic Process (GO:0009894)	1,39	HGS
Vesicle-Mediated Transport (GO:0016192)	1,37	HGS;PLIN3;SNX9
Microtubule Nucleation (GO:0007020)	1,37	NEDD1
Lymphocyte Activation Involved In Immune Response (GO:0002285)	1,37	LCP1
Acylglycerol Catabolic Process (GO:0046464)	1,35	LCP1
Acylglycerol Homeostasis (GO:0055090)	1,35	LCP1

Response To Hexose (GO:0009746)	1,35	LCP1
Negative Regulation Of Antigen Receptor-Mediated Signaling Pathway (GO:0050858)	1,35	GBP1
Endosomal Transport (GO:0016197)	1,34	HGS;SNX9
Negative Regulation Of Protein Localization To Membrane (GO:1905476)	1,33	GBP1
Wound Healing, Spreading Of Cells (GO:0044319)	1,33	LCP1
Negative Regulation Of TORC1 Signaling (GO:1904262)	1,33	SESN2
Phosphate-Containing Compound Metabolic Process (GO:0006796)	1,32	INPP4A;LCP1
Spindle Assembly Checkpoint Signaling (GO:0071173)	1,32	SPC25
Protein Targeting To Lysosome (GO:0006622)	1,32	HGS
Plasma Membrane Invagination (GO:0099024)	1,32	SNX9
Mitotic Spindle Assembly Checkpoint Signaling (GO:0007094)	1,32	SPC25
Mitotic Spindle Checkpoint Signaling (GO:0071174)	1,32	SPC25
Cellular Response To Acid Chemical (GO:0071229)	1,30	SESN2
Metallo-Sulfur Cluster Assembly (GO:0031163)	1,30	BOLA1
T Cell Activation Involved In Immune Response (GO:0002286)	1,30	LCP1
Positive Regulation Of TORC1 Signaling (GO:1904263)	1,30	SESN2
Cortical Cytoskeleton Organization (GO:0030865)	1,30	LCP1
Iron-Sulfur Cluster Assembly (GO:0016226)	1,30	BOLA1
Intracellular Monoatomic Cation Homeostasis (GO:0030003)	1,30	ATP6V0D1;CNNM4

Table S3: HPLC-MS/MS parameters for quantification of sphingolipids

Sphingolipid	Precursor ion (*m*/*z*)	Product ion (*m*/*z*) [a]	Retention time (min)	Calibration Reference Compound	Internal Standard
C17:0 Cer	534.5 $[M-H_2O+H]^+$	**264.3** / 282.3	14.9	-	-
C16:0 Cer	520.5 $[M-H_2O+H]^+$	**264.3** / 282.3	13.9	C16:0 Cer	C17:0 Cer
C18:0 Cer	548.5 $[M-H_2O+H]^+$	**264.3** / 282.3	15.9	C18:0 Cer	C17:0 Cer
C20:0 Cer	576.6 $[M-H_2O+H]^+$	**264.3** / 282.3	18.5	C20:0 Cer	C17:0 Cer
C22:0 Cer	604.6 $[M-H_2O+H]^+$	**264.3** / 282.3	21.6	C22:0 Cer	C17:0 Cer
C24:0 Cer	632.6 $[M-H_2O+H]^+$	**264.3** / 282.3	25.2	C24:0 Cer	C17:0 Cer
C24:1 Cer	630.6 $[M-H_2O+H]^+$	**264.3** / 282.3	21.8	C24:1 Cer	C17:0 Cer
d_{31}-C16:0 SM	734.6 $[M+H]^+$	86.1 / **184.0**	12.8	-	-
C16:0 SM	703.6 $[M+H]^+$	86.1 / **184.0**	13.0	C16:0 SM	d_{31}-C16:0 SM
C18:0 SM	731.6 $[M+H]^+$	86.1 / **184.0**	14.8	C18:0 SM	d_{31}-C16:0 SM
C20:0 SM	759.6 $[M+H]^+$	86.1 / **184.0**	17.1	C20:0 SM	d_{31}-C16:0 SM
C22:0 SM	787.7 $[M+H]^+$	86.1 / **184.0**	19.8	C22:0 SM	d_{31}-C16:0 SM
C24:0 SM	815.7 $[M+H]^+$	86.1 / **184.0**	23.2	C24:0 SM	d_{31}-C16:0 SM
C24:1 SM	813.7 $[M+H]^+$	**86.1** / 184.0	19.9	C24:1 SM	d_{31}-C16:0 SM

[a]Quantifiers are given in bold.

Table S4: HPLC-MS/MS parameters for quantification of diacylglycerols

Diacylglycerol	Precursor ion (*m*/*z*)	Product ion (*m*/*z*) [a]	Retention time (min)	Calibration Reference Compound	Internal Standard
d_5-C17:0 / C17:0	584.6 $[M-H_2O+H]^+$	**57.2** / 253.2	17.4	-	-
C16:0 / C16:0	551.5 $[M-H_2O+H]^+$	**57.2** / 239.2	14.6	C16:0 / C16:0	d_5-C17:0 / C17:0
C18:0 / C18:0	607.6 $[M-H_2O+H]^+$	**57.2** / 267.3	20.3	C18:0 / C18:0	d_5-C17:0 / C17:0
C16:0 / C18:0	579.5 $[M-H_2O+H]^+$	**239.2** / 267.3	17.5	C16:0 / C16:0	d_5-C17:0 / C17:0
C16:0 / C18:1	612.6 $[M+NH_4]^+$	**313.3** / 339.3	15.6	C16:0 / C18:1	d_5-C17:0 / C17:0
C16:0 / C18:2	610.5 $[M+NH_4]^+$	**313.3** / 337.3	13.8	C18:0 / C20:4	d_5-C17:0 / C17:0
C18:0 / C18:1	640.6 $[M+NH_4]^+$	339.3 / **341.3**	18.5	C16:0 / C18:1	d_5-C17:0 / C17:0
C18:0 / C18:2	638.6 $[M+NH_4]^+$	337.3 / **341.3**	16.6	C18:0 / C20:4	d_5-C17:0 / C17:0
C18:0 / C20:4	662.6 $[M+NH_4]^+$	**341.3** / 361.3	16.4	C18:0 / C20:4	d_5-C17:0 / C17:0
C18:1 / C18:1	638.6 $[M+NH_4]^+$	57.2 / **339.3**	16.5	C18:0 / C20:4	d_5-C17:0 / C17:0
C18:1 / C18:2	636.6 $[M+NH_4]^+$	337.3 / **339.3**	14.7	C18:0 / C20:4	d_5-C17:0 / C17:0
C18:2 / C18:2	634.6 $[M+NH_4]^+$	57.2 / **337.3**	12.9	C18:0 / C20:4	d_5-C17:0 / C17:0

[a]Quantifiers are given in bold.
