# Supplementary material for: The Role of Neutral Sphingomyelinase-2 (NSM2) in the Control of Neutral Lipid Storage in T Cells
Source: Int J Mol Sci. 2024 Mar 13;25(6):3247. doi: 10.3390/ijms25063247 (PMC10970209; doi:10.3390/ijms25063247)
Supplement: Supplementary file 1 [file ijms-25-03247-s001.zip › Schempp et al Supplementary Figures revised.pdf]

Supplementary Figures

The role of Neutral sphingomyelinase-2 (NSM2) in the control of neutral lipid storage in T cells

Rebekka Schempp [1], Janna Eilts [2], Marie Schöl [1], Maria Fernanda Grijalva Yépez [1], Agnes Fekete [3], Dominik Wigger [4], Fabian Schumacher [4], Burkhard Kleuser [4], Marco van Ham [5], Lothar Jänsch [5], Markus Sauer [2], Elita Avota [1*]

[1] Institute for Virology and Immunobiology, University of Würzburg, 97078 Würzburg, Germany
[2] Department of Biotechnology and Biophysics, Biocenter, University of Würzburg, 97074 Würzburg, Germany
[3] Pharmaceutical Biology, Julius-von-Sachs-Institute, Biocenter, University of Würzburg, 97082 Würzburg, Germany
[4] Institute of Pharmacy, Department of Pharmacology and Toxicology, Freie Universität Berlin, 14195 Berlin, Germany
[5] Cellular Proteome Research Group, Helmholtz Centre for Infection Research, 38124 Braunschweig, Germany
* Correspondence: elita.avota@uni-wuerzburg.de

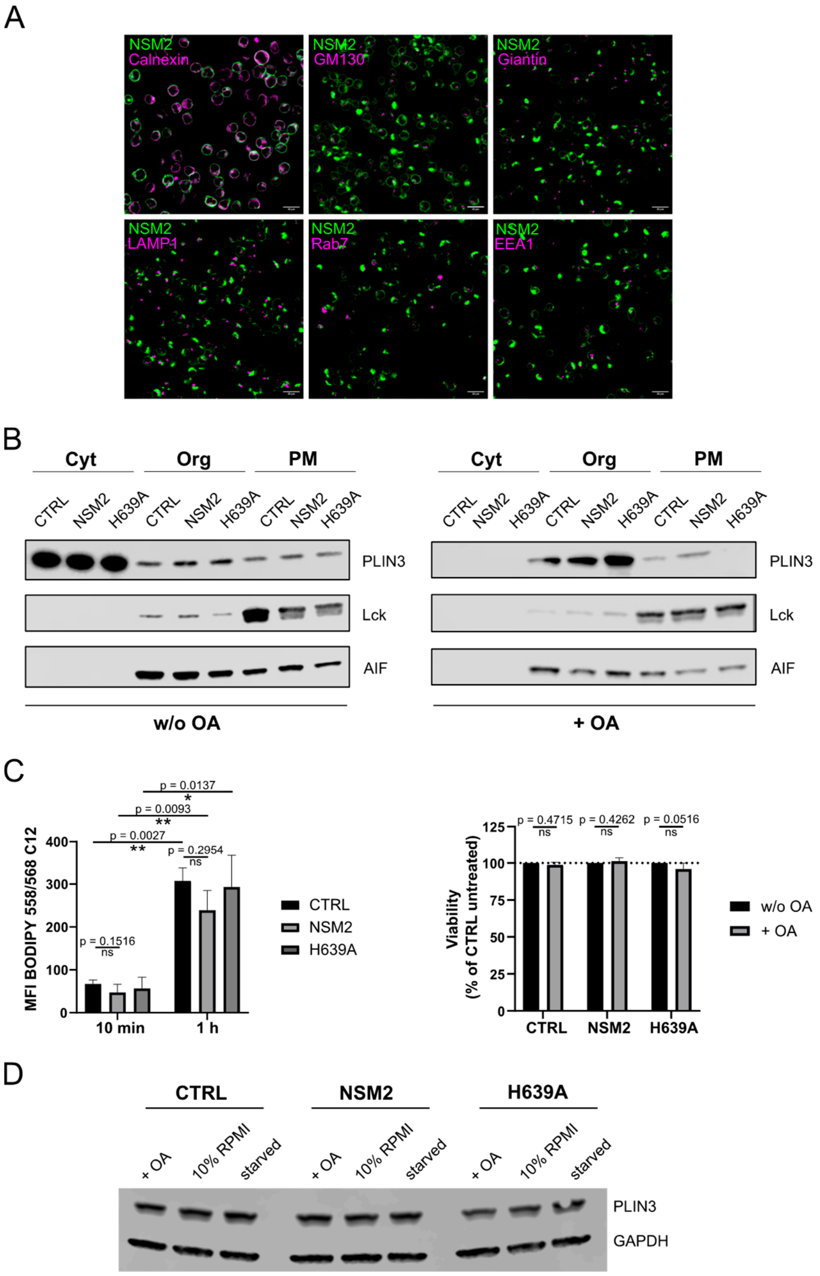

Figure S1. NSM2 overexpression does not affect lipid uptake or PLIN3 expression and subcellular localization. (**A**) Uncropped fluorescence images for Figure 2D of NSM2-GFP cells stained with different compartment markers (magenta) (scale bar = 20 µm). (**B**) Western blot analysis of PLIN3 distribution in subcellular fractions isolated from CTRL, NSM2 or H639A cells left untreated (left graph) or treated with 300 µM OA overnight. Cytoplasmic (Cyt), organelle (Org) and PM fractions are shown. (**C**) Flow cytometry analysis of lipid uptake in cells incubated with BODIPY 558/568 C12 fatty acid for 10 min or 1 h (left graph). Flow cytometry analysis of cell viability of Jurkat cells left untreated or treated with 300 µM OA overnight followed by Annexin V/PI staining (right graph). (**D**) Western blot analysis of PLIN3 protein levels in Jurkat cells either left untreated or treated with 300 µM OA or starved overnight. Mean values with standard deviations of three biological replicates are shown. *p*-values of two-way ANOVA with post hoc Sídák test are shown as * $p < 0.05$ and ** $p < 0.01$; ns, non-significant.

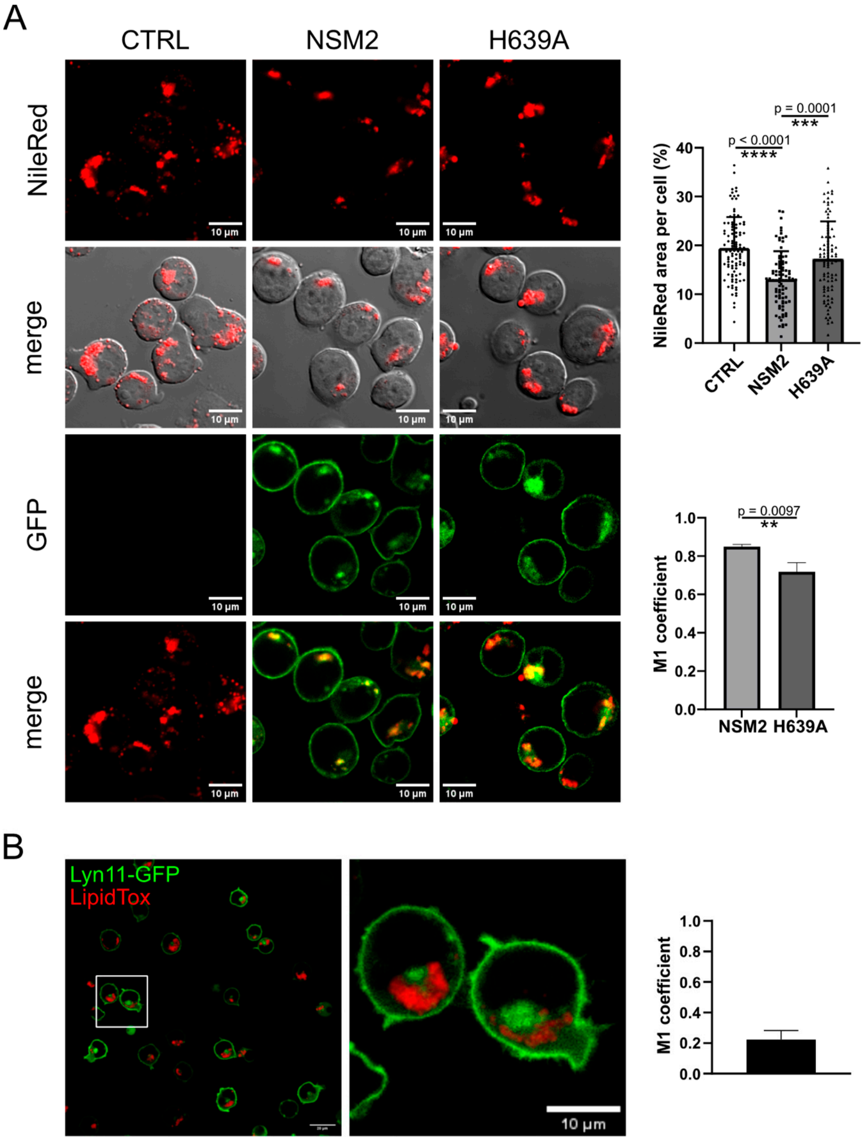

Figure S2. NSM2 but not plasma membrane associated Lyn kinase anchor co-localizes with lipid droplets (LDs). (**A**) Representative fluorescence images of CTRL, NSM2- and H639A-GFP (green) Jurkat cells loaded with 300 µM OA overnight and stained with neutral lipid dye NileRed (red) (scale bar: 10 µm). Quantification of NileRed area per cell in percent (upper graph; n = 88) and co-localization of NileRed and GFP (Mander's M1 coefficient) (bottom graph) are shown. *p*-values of one-way ANOVA with post hoc Turkey test are as ** $p < 0.01$, *** $p < 0.001$, and **** $p < 0.0001$. (**B**) Representative live cell fluorescence imaging images of Lyn11-GFP expressing Jurkat cells loaded with 300 µM OA overnight and labeled with HCS LipidTOX™ Deep Red (red) to visualize neutral lipids. Co-localization of LipidTOX and GFP was analyzed by using Mander's M1 coefficient. Scale bar: 20 µm. Rectangle indicates the position of cells shown in zoomed picture (right panel) (scale bars: 10 µm).

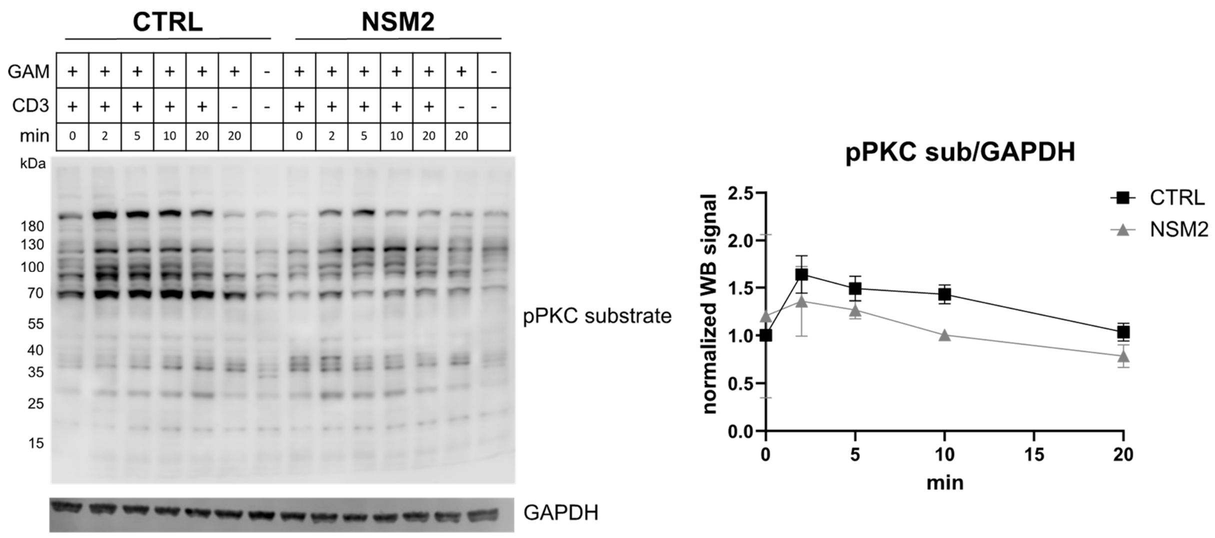

Figure S3. Overexpression of NSM2 impairs protein kinase C (PKC) activity. Western blot analysis of PKC substrate phosphorylation in total cell lysates of CTRL and NSM2 Jurkat cells stimulated with αCD3 for indicated time points. Representative Western blot is shown on the left. Right graph shows densiometric analysis of Western blot signals normalized against GAPDH levels. Mean values with standard deviations of three biological replicates are shown.

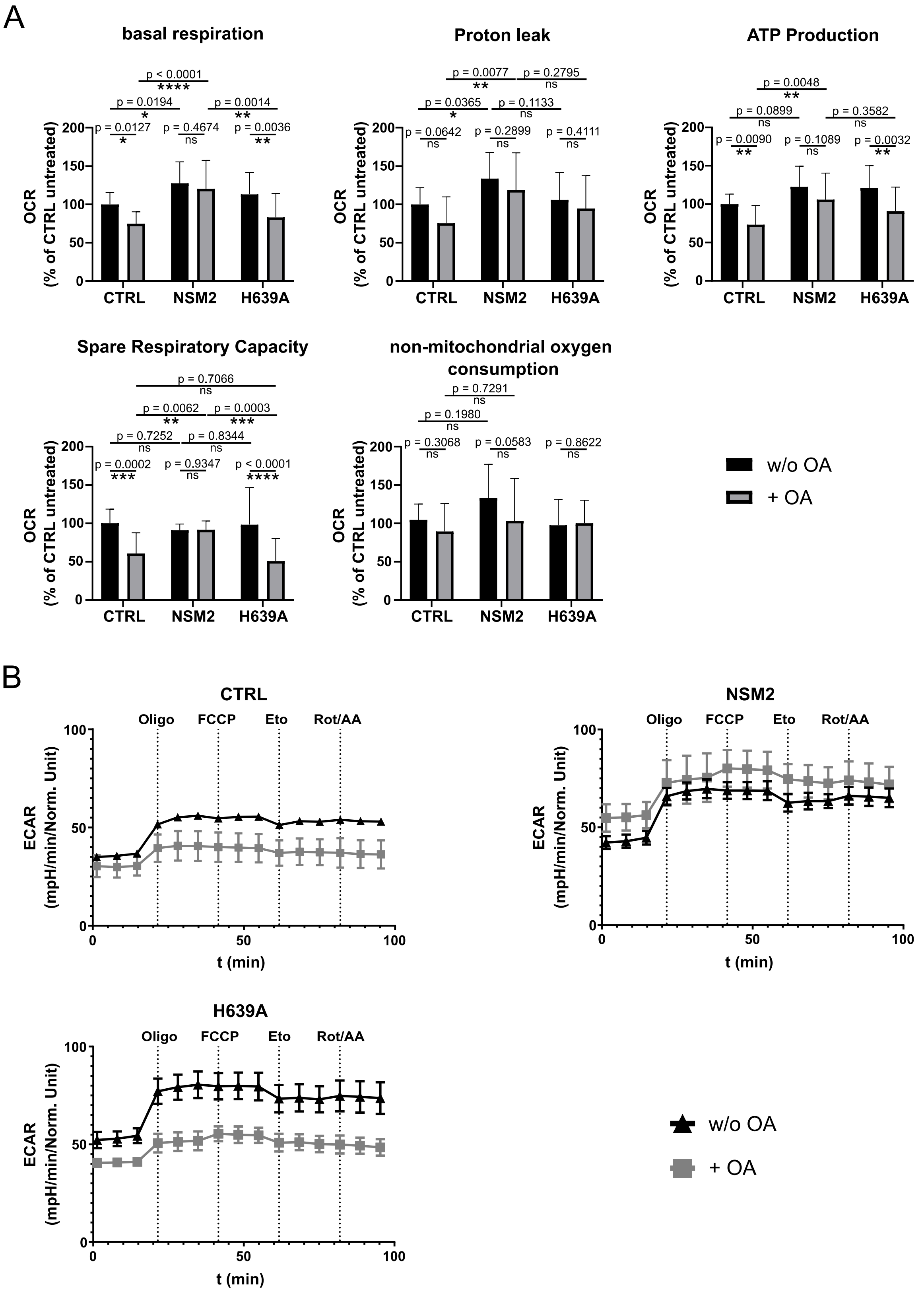

Figure S4. Mitochondrial respiration, ATP production and glycolytic activity are impaired in NSM2 overexpressing Jurkat cells. CTRL, NSM2 and H639A Jurkat cells were loaded with 300 µM OA overnight or left untreated before mitochondria stress test was performed (Figure 7B). (**A**) Analysis of basal respiration, proton leak, ATP production, spare respiratory capacity, and non-mitochondrial oxygen consumption from three independent experiments. p-values of two-way ANOVA with post hoc Sídák test are shown as * $p < 0.05$, ** $p < 0.01$, *** $p < 0.001$, and **** $p < 0.0001$; ns, non-significant. (**B**) Representative graphs of extracellular acidification rate (ECAR) measurements.

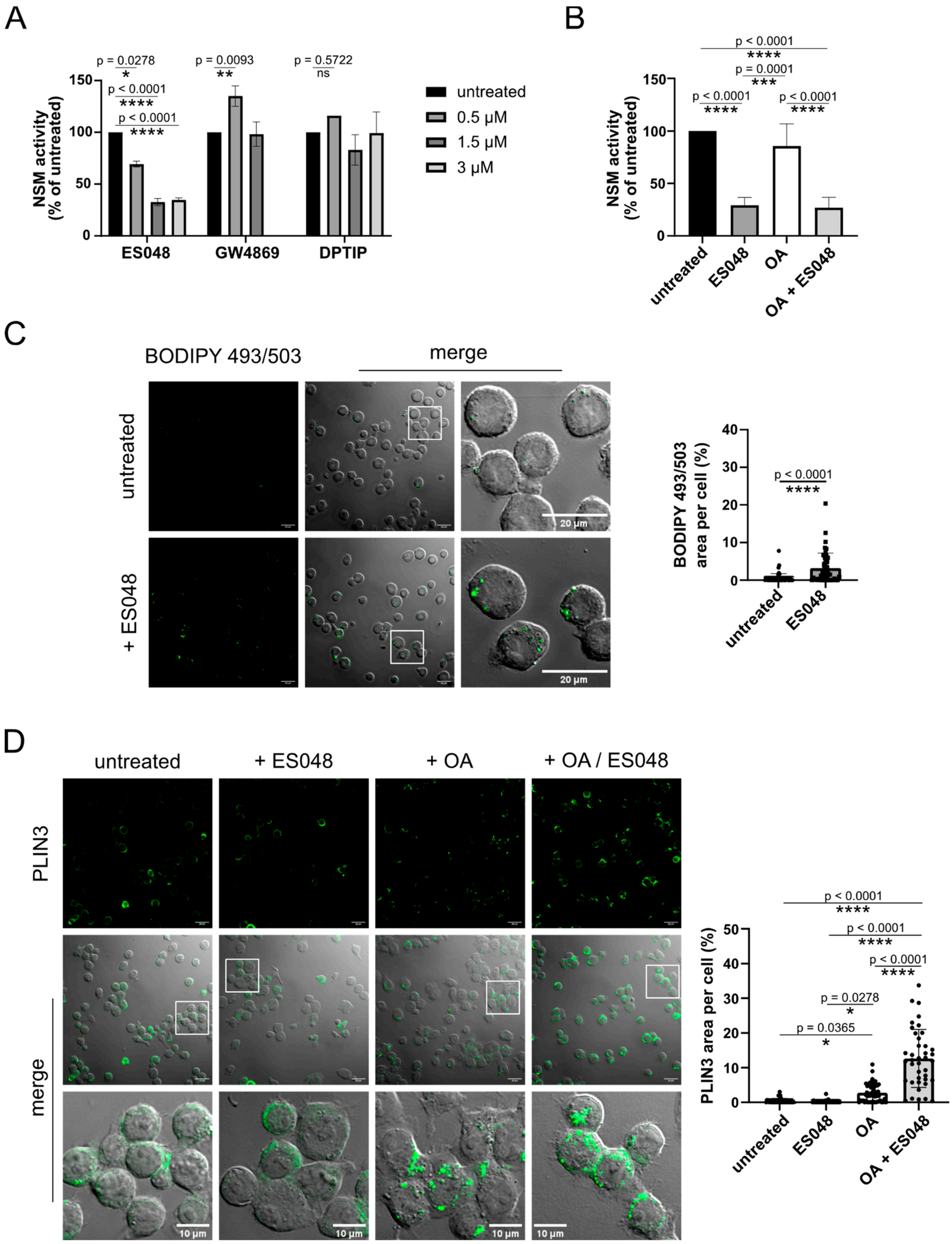

Figure S5. NSM2 inhibitor treatment promotes lipid droplet (LD) formation in human primary CD4+ T cells. (**A**) NSM activity of unstimulated CD4+ T cells treated with indicated concentrations of different NSM2 inhibitors (ES048, GW4869 or DPTIP) for 24 h (n = 3). (**B**) NSM activity of CD4+ T cells left untreated or treated with 1.5 μM ES048 and/or 50 μM OA and stimulated with αCD3/αCD28 for 24 h (n = 3). (**C**) Fluorescence images of CD4+ T cells left untreated or treated with 1.5 μM ES048 and stimulated with αCD3/αCD28 for 3 days. (**D**) CD4+ T cells were left untreated or treated with 1.5 μM ES048 and/or 50 μM OA and stimulated with αCD3/αCD28 for 3 days. Representative fluorescence images of cells stained with PLIN3 specific antibody (green) are shown (scale bar: 20 μm). Rectangles indicate the position of cells shown in bottom zoomed images (scale bar: 10 μm). Right graph shows quantification of PLIN3 fluorescence area per cell in percent (n = 33). Mean values with standard deviations of the measurements are shown in all graphs. p-values of one-way ANOVA with post hoc Turkey test are shown as * $p < 0.05$, ** $p < 0.01$, *** $p < 0.001$, and **** $p < 0.0001$; ns, non-significant.

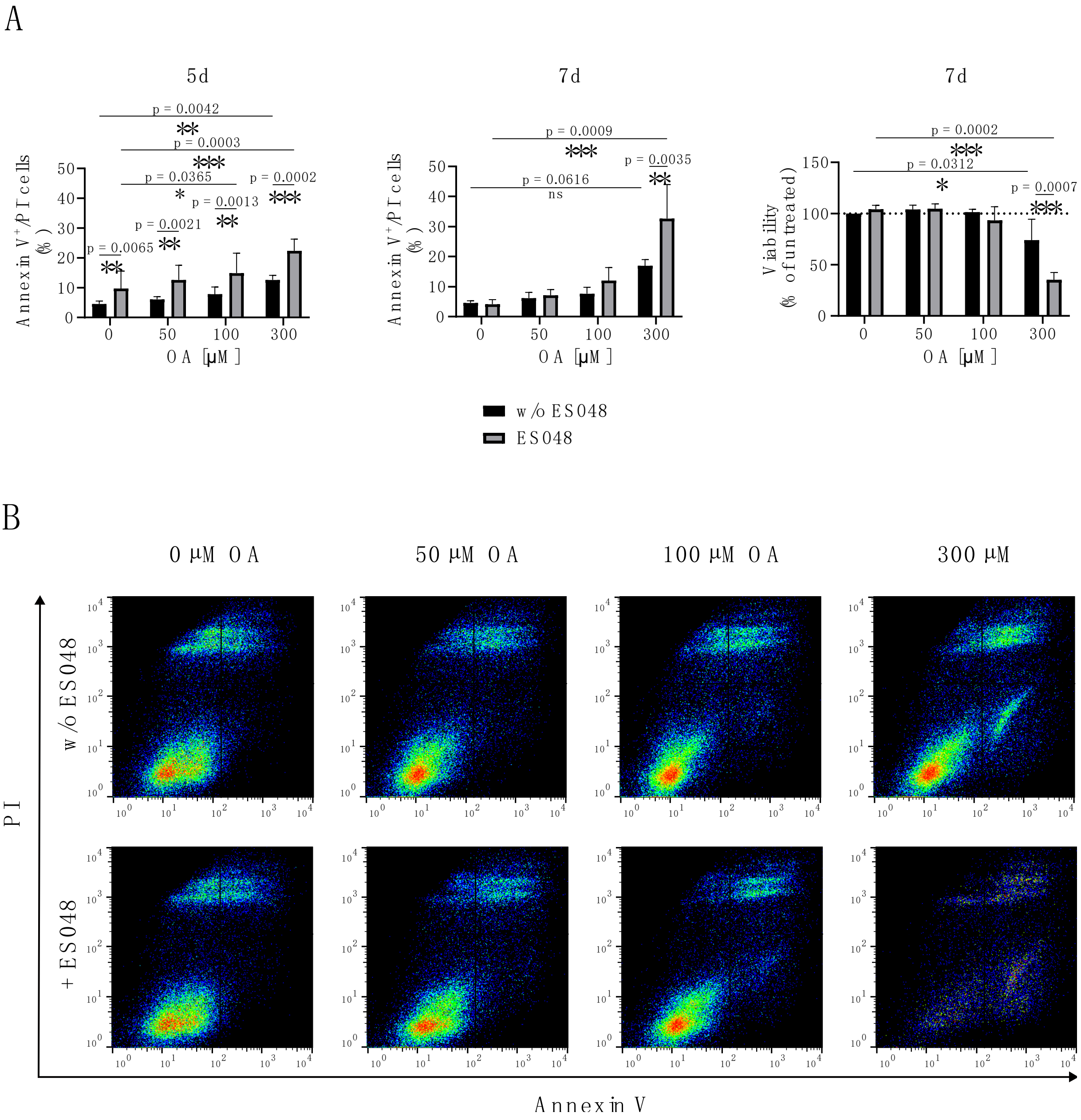

Figure S6. NSM2 inhibitor treatment enhances apoptosis in human primary CD4+ T cells induced by high doses of oleic acid (OA). (**A**) Apoptosis (left and middle graphs) and viability (right graph) were analyzed by flow cytometry of CD4+ T cells left untreated or treated with 1.5 μM ES048 and incubated with indicated concentrations of OA and stimulated with αCD3/αCD28 for 5 and 7 days (n = 3). Mean values with standard deviations of the measurements are shown in all graphs. p-values of two-way ANOVA with post hoc Sídák test are shown as * $p < 0.05$, ** $p < 0.01$, *** $p < 0.001$, and **** $p < 0.0001$; ns, non-significant. (**B**) Representative flow cytometry dot plots of CD4+ T cells after 7 days of stimulation.

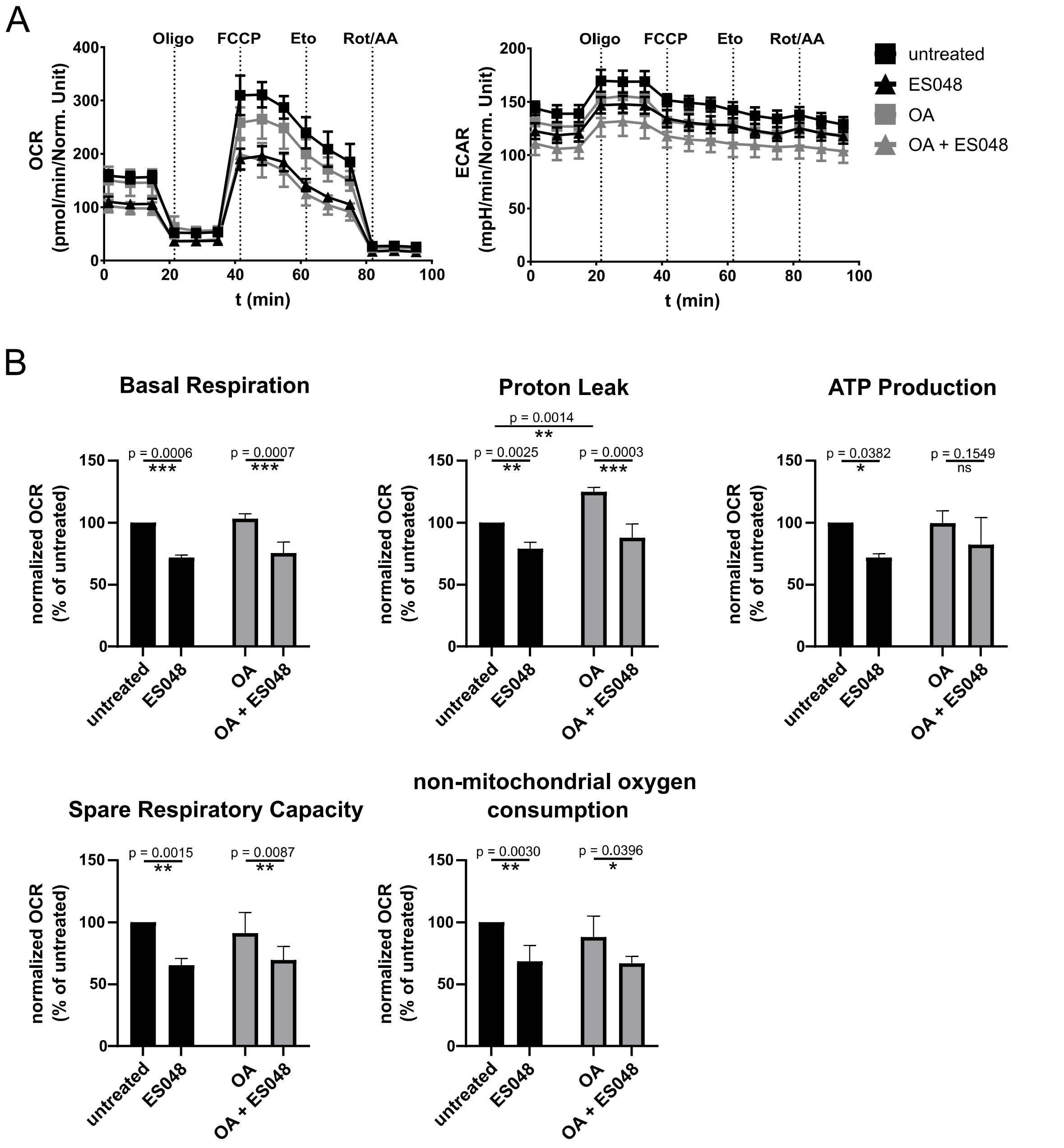

Figure S7. Endogenous NSM2 in human primary CD4+ T cells promotes mitochondria functionality. CD4+ T cells were left untreated or pretreated with 1.5 μM ES048 for 2h prior to co-stimulation with αCD3/αCD28 and loading with 50 μM OA for 3 days followed by Seahorse mitochondria stress test (Figure 8E). (**A**) Representative graphs of oxygen consumption rate (OCR) and extracellular acidification rate (ECAR) measurements. (**B**) Analysis of basal respiration, proton leak, ATP production, spare respiratory capacity and non-mitochondrial oxygen consumption from three independent experiments. p-values of two-way ANOVA with post hoc Sídák test are shown as * $p < 0.05$, ** $p < 0.01$, *** $p < 0.001$, and **** $p < 0.0001$; ns, non-significant.
